# Trends in the Intraindividual Double Burden of Overweight/Obesity and Anemia among Adult Women Living in 33 Low- and Middle-Income Countries: A Secondary Analysis of Demographic and Health Surveys from 2000-2019

**DOI:** 10.1016/j.tjnut.2023.02.012

**Published:** 2023-02-15

**Authors:** Ana Irache, Seun Stephen Anjorin, Rishi Caleyachetty, Paramjit Gill

**Affiliations:** Warwick Centre for Global Health, Warwick Medical School, University of Warwick, Medical School Building, Coventry, United Kingdom

**Keywords:** overweight, obesity, anemia, adult women, trends, double burden of malnutrition, Demographic and Health Surveys

## Abstract

**Background:**

Changes in overweight/obesity and anemia among women have been investigated in multiple studies, but the rate at which their coexistence at the individual level has evolved remains unknown.

**Objectives:**

We aimed to *1*) document trends in the magnitude and inequalities of the co-occurrence of overweight/obesity and anemia; and *2*) compare these with overall trends in overweight/obesity, anemia, and the co-occurrence of anemia with normal weight or underweight.

**Methods:**

For this cross-sectional series study, we used 96 Demographic and Health Surveys from 33 countries with available anthropometric and anemia data among nonpregnant adult women (20–49 y old; n = 1,648,308). The primary outcome was defined as the coexistence of overweight or obesity (BMI ≥25kg/m^2^) and anemia (hemoglobin concentrations <12.0 g/dL) within the same individual. We computed overall and regional trends through multilevel linear regression models and by sociodemographic characteristics (i.e., wealth, education, and residence). Estimates at the country level were calculated through ordinary least square regression models.

**Results:**

From 2000 to 2019, the co-occurrence of overweight/obesity and anemia increased modestly at an annual rate of 0.18 percentage points (95% CI: 0.08, 0.28 percentage points; *P* < 0.001), ranging from 0.73 percentage points in Jordan to -0.56 percentage points in Peru. This trend occurred in parallel with overall increases in overweight/obesity and reductions in anemia. The co-occurrence of anemia with normal weight or underweight was reducing in all countries, except Burundi, Sierra Leone, Jordan, Bolivia, and Timor-Leste. Stratified analyses yielded an upward trend in the co-occurrence of overweight/obesity and anemia across all subgroups but particularly in women from the 3 middle wealth groups, no education, and capital city or rural residents.

**Conclusions:**

The rising trend in the intraindividual double burden suggests that efforts to reduce anemia among women living with overweight/obesity may need to be revisited to accelerate progress toward the 2025 global nutrition target of halving anemia.

## Introduction

Low- and middle-income countries (LMICs) are experiencing high rates of undernutrition and micronutrient deficiencies alongside overweight/obesity at the individual, household, and population levels [1]. Women living in these countries bear a high burden of malnutrition, with anemia and overweight/obesity being 2 of the most common nutritional problems in this population [2]. The coexistence of both conditions at the 3 levels are well documented [[3], [4], [5], [6], [7], [8]]. At the individual level, the double burden of overweight/obesity and anemia varies widely by country, but the data points to particularly high rates among adult women (20–49 y old) when compared to adolescent girls (15–19 y old), reaching prevalence estimates as high as 30.0% in some LMICs [7].

The intraindividual double burden of overweight/obesity and anemia merits more investigation in view of the 2025 global nutrition targets and the rapidly rising trends in overweight/obesity, particularly among women of reproductive age. According to a recent study, little progress has been achieved over the past 2 decades in reducing anemia [9]. Therefore, accelerated progress is needed to achieve the global nutrition target of halving the prevalence of anemia among women of reproductive age. The latter calls for a better understanding of the causes of anemia and lack of progress.

Nutritional anemias (e.g., those resulting from deficiencies of different micronutrients, including iron) are one of the most common type of anemias worldwide; although it varies widely by geographical setting and infectious disease burden [10,11]. Accumulating evidence also indicates that inflammation plays an important role in the etiology of anemia (e.g., by hindering absorption of micronutrients from the diet) [10]. Overweight and obesity are characterized by chronic low-grade inflammation [12], and although the association between both forms of malnutrition (i.e., overweight/obesity and anemia) remains unclear, obesity-related inflammation has been linked with impaired iron absorption and deficiency [13,14]. Despite this, the rate at which the coexistence of overweight/obesity and anemia at the individual level has evolved remains unknown.

Therefore, in this study we aimed to: *1*) document trends in the co-occurrence of overweight/obesity and anemia among adult women living in LMICs, overall and by sociodemographic characteristics (i.e., wealth quintiles, education levels, and area of residence), and *2*) compare these with overall trends in overweight/obesity, anemia, and the co-occurrence of anemia with normal weight or underweight.

## Methods

### Study design and participants

For this trend analysis, all Demographic and Health Surveys (DHSs) from every LMIC were used for which at least 2 datasets were publicly available (https://dhsprogram.com). Datasets were individually checked, and only those with anthropometric and anemia values among women of reproductive age were used. We retrieved a total of 96 DHS datasets from 33 countries conducted between 2000 and 2019 (Supplemental Table 1). DHSs are nationally representative household surveys implemented approximately every 5 y in over 90 LMICs that provide information on different health and population indicators, including nutrition.

The study population encompassed adult women (20–49 y old) with available data on both anthropometry (height and weight) and anemia (hemoglobin concentrations). We excluded women who were younger than 20 y old and those who were pregnant or who had given birth 2 mo preceding data collection, due to weight gain [15].

Ethical approval was not sought for this analysis of secondary data. The DHSs were approved centrally by ICF International institutional review board and by individual boards within every participating country. All data used for this analysis was deidentified.

### Procedures

The primary outcome, intraindividual double burden of overweight/obesity and anemia, was defined as a woman being affected by both conditions at the time of data collection. Height and weight variables were used to compute the BMI. The DHS team collected anthropometric data of women using a ShorrBoard measuring board and a SECA 878 digital scale for height and weight, respectively, following the latest World Health Organization (WHO)–UNICEF guidelines for anthropometric data collection [16,17]. Overweight/obesity was defined as a BMI of 25 kg/m^2^ or higher [18]. The diagnosis of anemia was confirmed by measuring hemoglobin concentrations using HemoCue 201+ or the 301+ system through a stick capillary blood sample. Anemia was defined as hemoglobin concentrations (adjusted by altitude and smoking) lower than 12.0 g/dL [19].

Prevalence estimates with 95% CIs were first calculated for the primary outcome (i.e., co-occurrence of overweight/obesity and anemia), overweight/obesity, and anemia, for each country and each time point. Stratified estimates were then quantified in the co-occurrence of overweight/obesity and anemia by household wealth, education level, and area of residence. We used the variable household wealth as presented in DHS, computed by principal component analysis of household characteristics and assets, and divided into quintiles from the poorest (Q1 or first quintile) to the richest (Q5 or fifth quintile) [20]. Education level was assessed by self-report of the completed educational level and converted into 3 groups (E1: no education; E2: primary; E3: secondary or higher) by merging the 2 higher categories to avoid low sample sizes (<25 participants). Place of residence, available as urban or rural in the surveys according to country specific definitions, was further categorized as capital city, other urban, and rural. Capital city was defined differently across countries and referred to either the capital, largest, or economic city (Supplemental Table 2). Further, when the capital city was not isolated in a survey, we identified urban residents living in the region where the capital city is located instead.

### Statistical analysis

Trends in the magnitude of the intraindividual double burden of overweight/obesity and anemia among adult women were assessed for every country, through ordinary least square regression models (i.e., prevalence of the co-occurrence of overweight/obesity and anemia regressed on survey year) to estimate the average annual rate of change (AARC) in percentage points. To assess trends from the period 2000 to 2019 across all LMICs and by WHO regions, the AARC in intraindividual double burden of overweight/obesity and anemia prevalence was computed as the slope of the multilevel linear regressions of outcome prevalence on survey year, with survey time point as the first level and country as the second level. We repeated the same process to document overall and regional trends in the bivariate prevalence of overweight/obesity and anemia. Separate multilevel linear regressions of the primary outcome prevalence were also run for each household wealth, education level, and area of residence subgroup. A positive AARC value depicts an increase in percentage points in the co-occurrence of overweight/obesity and anemia over time; whereas a negative value means that the prevalence has been decreasing.

To further investigate within-country-level changes in the prevalence of co-occurrent overweight/obesity and anemia over time, trends in absolute inequalities in the primary outcome were also documented. The slope index of inequality (SII) was first calculated through logistic regression of co-occurrent overweight/obesity and anemia prevalence as the outcome and household wealth or education level as the independent variable, providing with the absolute difference in percentage points between the fitted values of the top and the bottom of the wealth and education distribution [21,22]. Positive SII values indicate that the prevalence of co-occurrent overweight/obesity and anemia at the top of the socioeconomic indicator is *n* percentage points higher than at the bottom, whereas negative values depict the opposite. For the area of residence, inequality gaps were computed as the absolute difference in percentage points between the intraindividual double burden prevalence in urban vs. rural. A positive gap value depicts a higher prevalence of co-occurrent overweight/obesity and anemia in adult women from urban areas, whereas a negative gap value depicts a higher prevalence among women living in rural areas. Annual changes in SII and gaps were derived from ordinary least square regression models in a similar manner to the estimation of trends in the prevalence of co-occurrent overweight/obesity and anemia for every country. Positive AARC values indicate that inequalities are widening over time, whereas negative values depict that inequalities are narrowing.

All analyses were conducted in Stata version V.16.0. (Statacorp). We used sampling weights and the Stata’s survey estimation procedures (‘svy’ command) throughout the analyses to account for the clustering and stratification in the sample design of the DHSs.

We reported the study according to the Strengthening the Reporting of Observational Studies in Epidemiology (STROBE) reporting guideline for cross-sectional studies [23].

## Results

The analytical sample comprised a total of 1,648,308 nonpregnant adult women (20–49 y old) with available anthropometric and anemia data living across 33 LMICs from 2000 to 2019 (Supplemental Figure 1). The total number of countries represented in this analysis per WHO region included 21 from the African region (*n* = 58 datasets; 238,801 women), 2 from the Eastern Mediterranean region (*n* = 7 datasets; 35,217 women), 2 from the European region (*n* = 5 datasets; 32,657 women), 4 from the Americas region (*n* = 14 datasets; 147,428 women), 3 from the Southeast Asian region (*n* = 8 datasets; 1,170,383 women) and 1 from the Western Pacific region (*n* = 4 datasets; 23,822 women).

**FIGURE 1:**
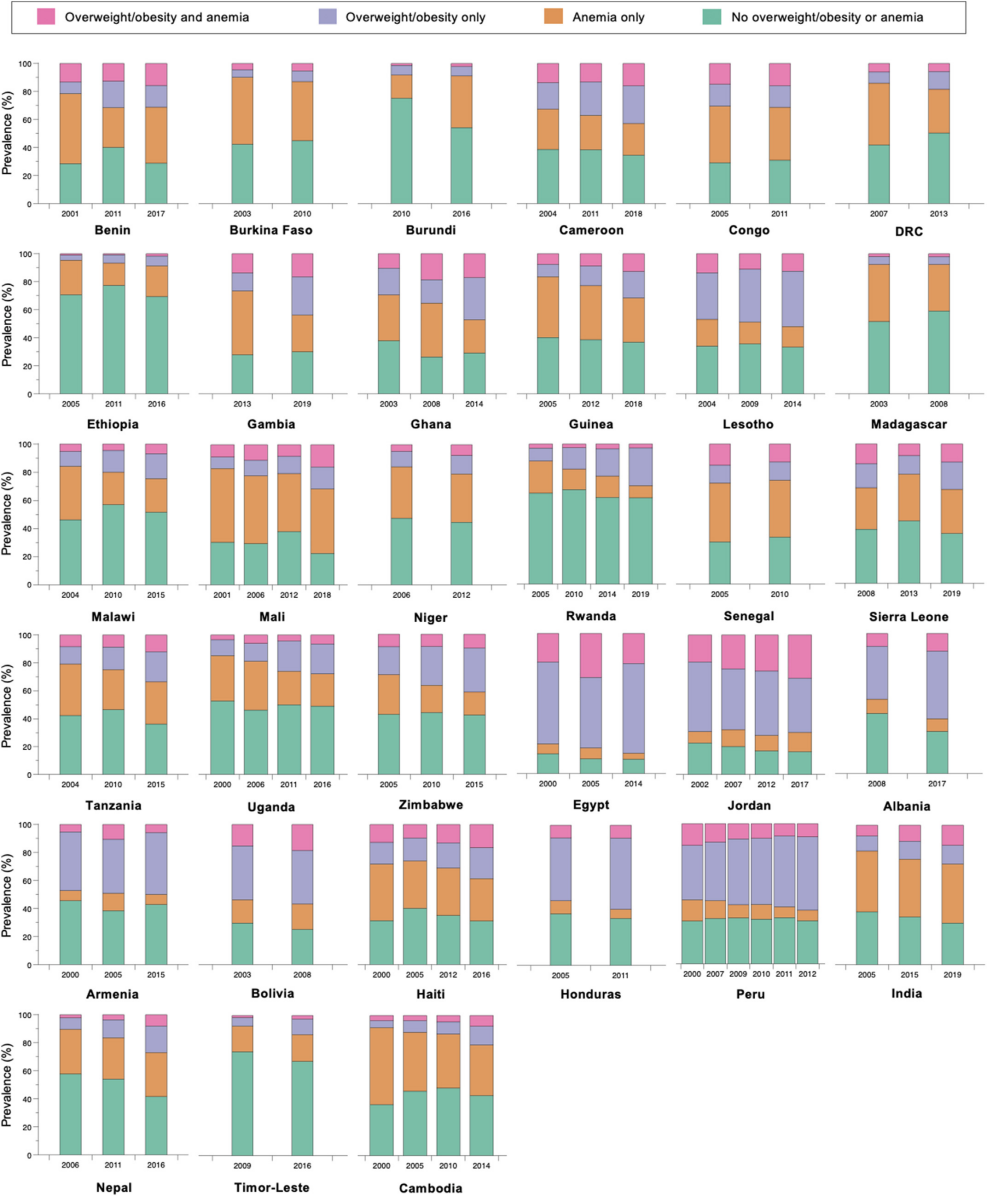
Prevalence of overweight/obesity and anemia among nonpregnant adult women (20–49 y old) living in 33 low- and middle-income countries.

Figure 1 shows the proportion of adult women living with overweight/obesity and/or anemia in the 96 datasets included in the analysis. The prevalence of intraindividual double burden of overweight/obesity and anemia varied greatly by geographical region, country, and year of survey, and national prevalence estimates masked important within-country inequalities by the 3 sociodemographic characteristics (full results are available in Supplemental Tables 3–7 and Supplemental Figures 2–4).

**FIGURE 2:**
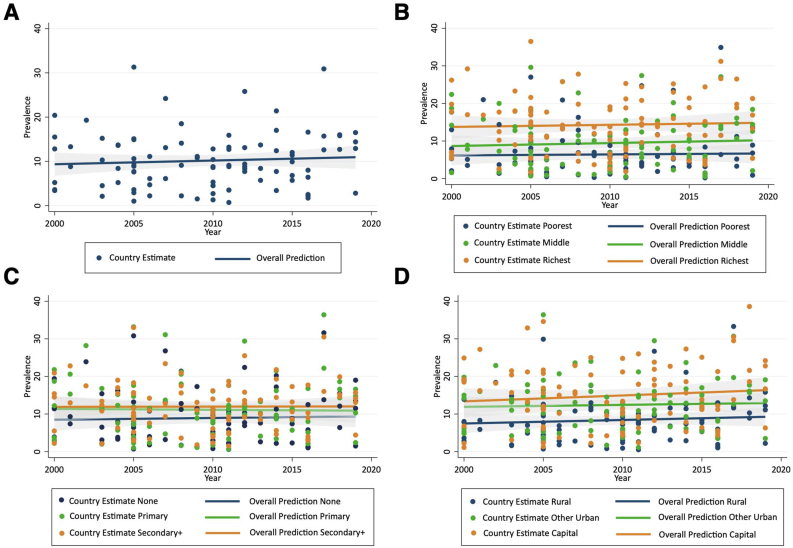
Trends in the prevalence of co-occurrent overweight/obesity and anemia overall (A) and by household wealth (B), education level (C), and area of residence (D) among adult women (20–49 y old) living in 33 low- and middle-income countries.

Overall, the prevalence of co-occurrent overweight/obesity and anemia among adult women living in the 33 LMICs included in the study increased modestly at an annual rate of 0.18 percentage points (95% CI: 0.08, 0.28 percentage points; *P* < 0.001) over the past 2 decades, whereas the overall prevalence of overweight/obesity increased rapidly (AARC = 0.73 percentage points, 95% CI: 0.60, 0.86 percentage points; *P* < 0.001), and the prevalence of anemia declined at a slower pace (AARC = −0.33 percentage points, 95% CI: −0.57, −0.09 percentage points; *P* < 0.05) (Table 1). Figure 2 illustrates the trend of co-occurrent overweight/obesity and anemia prevalence in the 33 countries, overall and by the 3 sociodemographic characteristics, where the slopes of the trend line represent the AARC in the primary outcome. These show increases in co-occurrent overweight/obesity and anemia across all subgroups, particularly those in the second wealth group (0.21 percentage points), women with no education (0.17 percentage points), and capital and rural residents (0.19 percentage points) (Table 1).

**Table 1:**
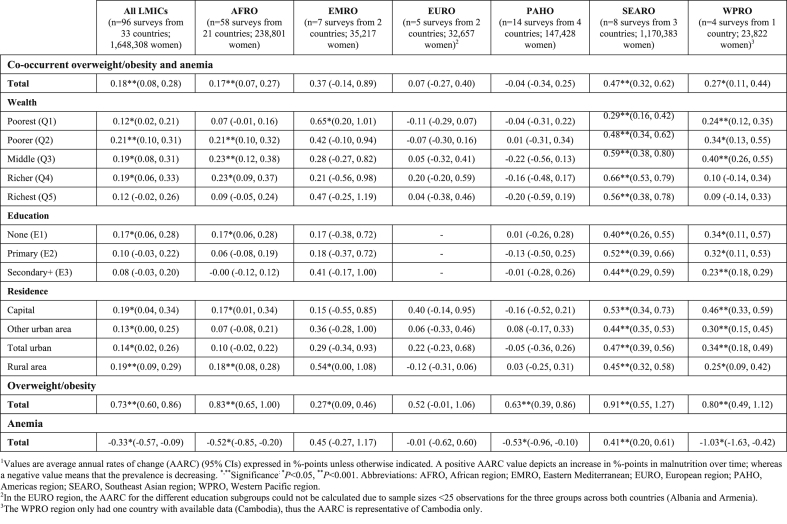
Average annual rate of change (AARC) in the prevalence of intra-individual double burden of overweight/obesity and anemia, overweight/obesity, and anemia among adult women (20–49 y old)^1^

At the regional level, the trend in co-occurrent overweight/obesity and anemia was upward in all WHO regions, with the exception of the Americas (Table 1; Supplemental Figures 5–10). The Southeast Asian region experienced the most rapid rise in the co-occurrence of overweight/obesity and anemia over the past 2 decades (AARC = 0.47 percentage points, 95% CI: 0.32, 0.62 percentage points; *P* < 0.001), with substantial increases in both overweight/obesity (AARC = 0.91 percentage points, 95% CI: 0.55, 1.27 percentage points; *P* < 0.001) and anemia (AARC = 0.41 percentage points, 95% CI: 0.20, 0.61 percentage points; *P* < 0.001). In the African region, countries in West and Central Africa had almost 3 times more rapid rise in co-occurrent overweight/obesity and anemia than countries located in the Eastern and Southern African subregion over the past 2 decades (0.24 percentage points vs. 0.09 percentage points) (Supplemental Table 8). Heterogeneity in trends across the different population subgroups in WHO regions are summarized in Table 1. Significant increases in co-occurrent overweight/obesity and anemia appear to be fastest among those in 3 middle wealth groups, women with no education, and rural and capital city residents in the African region; those in the poorest wealth group and women living in rural areas in the Eastern Mediterranean region; all population subgroups in the Southeast Asian region, but particularly among women in the 3 richest wealth groups, those with primary education, and capital city residents; and all population subgroups with the exception of women in the 2 richest wealth groups in the Western Pacific region, particularly, women in the middle wealth group, those with no education or primary education, and capital city residents. The European and Americas region do not show a clear pattern, which may be a reflection of the differences observed in trends for the different population subgroups across countries included (Supplemental Tables 5–7).

**FIGURE 3:**
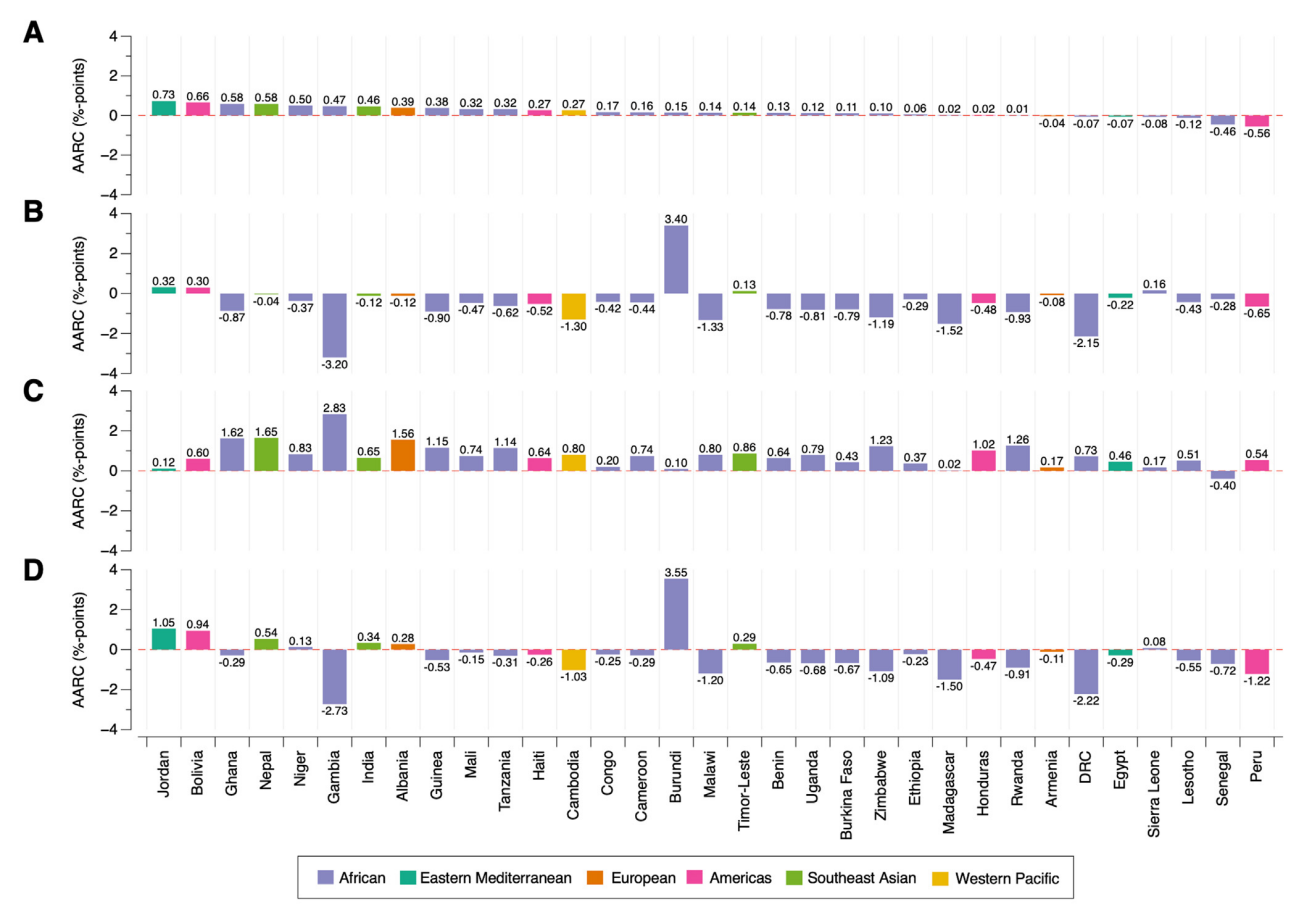
Country-level average annual rate of change (AARC) in the prevalence of co-occurrent overweight/obesity and anemia (A), co-occurrent anemia and normal weight or underweight (B), overweight/obesity (C), and anemia (D) among adult women (20–49 y old) living in 33 low- and middle-income countries.

At the national level, there were upward changes in the prevalence of co-occurrent overweight/obesity and anemia in 78.8% (26/33) of countries investigated, and downward changes in 21.2% (7/33) (Supplemental Table 4). Figure 3 displays the range of AARC in the prevalence of co-occurrent overweight/obesity and anemia in percentage points, from 0.73 in Jordan to -0.56 in Peru (African region: highest in Ghana [0.58 percentage points] and lowest in Senegal [-0.46 percentage points]; Eastern Mediterranean region: highest in Jordan [0.73 percentage points] and lowest in Egypt [-0.07 percentage points]; European region: highest in Albania [0.39 percentage points] and lowest in Armenia [-0.04 percentage points]; Americas region: highest in Bolivia [0.66 percentage points] and lowest in Peru [-0.56 percentage points]; Southeast Asian region: highest in Nepal [0.58 percentage points] and lowest in Timor-Leste [0.14 percentage points]; and Western Pacific: Cambodia [0.27 percentage points]). Of the 26 countries with an upward trend in the co-occurrence of overweight/obesity and anemia, 18 also experienced a decrease in overall anemia (Figure 3; Supplemental Table 4). The remaining 8 countries showed an increase in overall anemia, yet, the co-occurrence of anemia and normal weight or underweight decreased in Nepal, Niger, India, and Albania. Similarly, of the 7 countries with a downward change in the co-occurrence of overweight/obesity and anemia, only Sierra Leone experienced a modest increase in the co-occurrence of anemia and normal weight or underweight.

Trends in absolute inequality measures at the national level are provided in full in Supplemental Tables 5–7. Overall, results were mixed and with no clear pattern, with wealth, education, and residence-related inequalities widening in 20, 17, and 17 countries while narrowing in 13, 14, and 16, respectively (Supplemental Figures 2–4).

## Discussion

Using DHS data from 33 LMICs that had at least 2 surveys with available anemia and anthropometric measures among adult women (20–49 y old), we found that the prevalence of the intraindividual double burden of overweight/obesity and anemia increased modestly at an annual rate of 0.18 percentage points from 2000 to 2019. Simultaneously, overweight/obesity increased at an annual rate of 0.73 percentage points and anemia declined at -0.36 percentage points annually. Of the 33 countries included in the analysis, the co-occurrence of overweight/obesity and anemia increased in 26 countries and decreased in 7. Jordan experienced the largest upward trend in co-occurrent overweight/obesity and anemia (AARC = 0.73 percentage points), whereas Peru experienced the largest decline (AARC = −0.56 percentage points).

**FIGURE 4:**
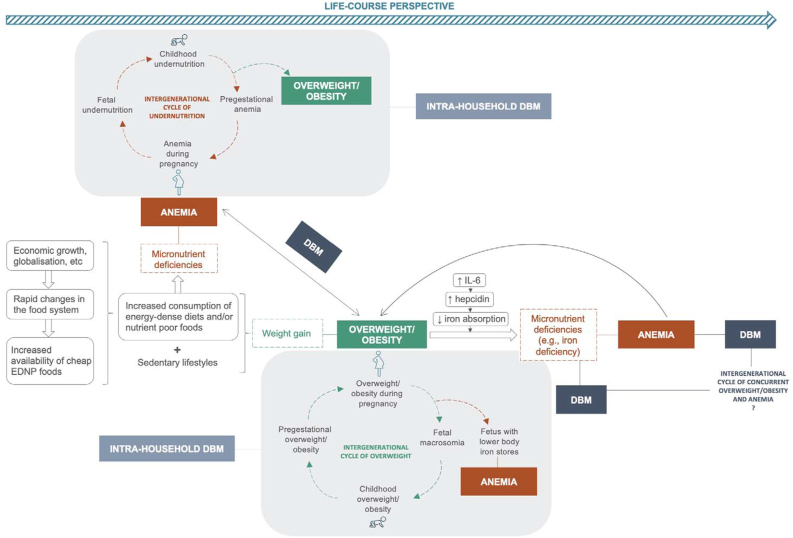
Interconnections between overweight/obesity and anemia across the life course and intergenerational manifestation. DBM, double burden of malnutrition defined as the co-occurrence of overweight/obesity and anemia.

To our knowledge, this is the first study documenting trends in the intraindividual double burden of overweight/obesity and anemia among adult women. Overall upward trends in the prevalence of co-occurrent overweight/obesity and anemia, in a context of declining or stagnant overall anemia trends, suggest that the nutritional needs of women living with overweight/obesity are not necessarily being met. We highlight 2 main interconnected pathways by which overweight/obesity and nutritional anemias may coexist within individuals in Figure 4: poor diets and overweight/obesity-mediated inflammation.

First, it is widely acknowledged that the nutrition transition that LMICs are undergoing is a key driver of the rapid rise in overweight/obesity prevalence [1,24]. This shift from traditional foods to diets characterized by energy-dense and nutrient-poor foods are likely a key contributor not only of the obesity epidemic but also micronutrient deficiencies. Recent data from the Global Diet Quality Project showed that in many countries, a high proportion of women are not getting the diverse diets that they need, which in turn increases their risk of vitamin and mineral deficiencies [25]. Empirical evidence from LMICs and high-income countries also supports an association between the consumption of ultraprocessed foods and risk of overweight/obesity and nutrition-related noncommunicable diseases (NCDs) [[26], [27], [28]] and inadequate intake of micronutrients [29,30].

Second, overweight/obesity has been previously linked to iron deficiency [[31], [32], [33]], which can co-occur within individuals regardless of dietary iron intake [34]. Different hypotheses for the association between overweight/obesity and iron deficiency have been investigated and are summarized elsewhere [35]. In brief, obesity, characterized by chronic low-grade inflammation (elevated IL-6), increases hepcidin (an iron-regulating peptide hormone), reducing intestinal iron absorption, and eventually, leading to systemic iron deficiency and/or iron restricted erythropoiesis [32,36,37]. The link between overweight/obesity and anemia or iron-deficiency anemia is less clear, although iron deficiency can develop into anemia over time [38]. Multiple studies have consistently found an association between overweight/obesity and the features of anemia of inflammation (e.g., elevated serum ferritin and decreased serum iron); yet, hemoglobin levels do not seem to be significantly different in individuals living with overweight/obesity when compared with those with normal weight [13]. Nevertheless, the vast body of evidence on the association between overweight/obesity and anemia is based on cross-sectional studies, which do not take into account “time” as a factor (i.e., disease progression), nor any changes in diets or nutritional status over time within individuals.

In the present study, we showed reductions in the co-occurrence of anemia and normal weight or underweight in all countries except from Burundi, Sierra Leone, Jordan, Bolivia, and Timor-Leste alongside increases in co-occurrent overweight/obesity and anemia over the same time period. Therefore, another plausible explanation for the observed rise in co-occurrent overweight/obesity and anemia could be that current initiatives to reduce anemia are not as effective among this population when compared to women in other BMI categories. Main global public health nutrition initiatives to address nutritional anemias include supplementation (e.g., iron and folic acid or multiple micronutrients) or fortification of staple foods or condiments, among others [39]. There are, however, some studies suggesting significant lower efficacy of these interventions when used among individuals with overweight/obesity and who are under chronic inflammation [40,41]. The evidence on the latter is scarce, and thus, this merits more exploration. Women living with overweight/obesity may benefit from weight reduction programs to either prevent or help restore micronutrient homeostasis [[42], [43], [44]].

The trend analysis by sociodemographic characteristics presented in this study yielded increases in the co-occurrence of overweight/obesity and anemia across all subgroups, but particularly among the 3 middle wealth groups, women with no education, and those living in capital cities or rural areas; however, findings were not consistent across countries and WHO regions. The observed stratified trends seem to emulate those observed for overweight/obesity in LMICs, whereby the burden has been rising more rapidly among capital city residents and women with no education [45,46] but also appear to be shifting toward rural areas in certain regions [47]. Despite these trends, the magnitude of the co-occurrence of overweight/obesity and anemia among adult women remains concentrated largely among the highest wealth quintiles, higher education levels, and urban areas, emulating the current burden of overweight/obesity [48]. Only for a few countries mostly located within the Eastern Mediterranean and European regions, changes in this distribution occurred over the past 2 decades by the 3 sociodemographic characteristics. Differences observed in the burden of co-occurrent overweight/obesity and anemia across regions and within countries might be related to the different nutritional and nonnutritional factors involved in the etiology of anemia [10].

This study represents the first comprehensive trends analysis of the magnitude and inequalities in the intraindividual double burden of overweight/obesity and anemia among adult women (20–49 y old) living in 33 LMICs across different WHO regions over the past 2 decades. This is a strength of the study, filling a gap in research and contributing to the growing literature on the double burden of overweight/obesity and anemia, which had historically focused primarily on the double burden of malnutrition at the population level or that examined nutritional anemias as a form of undernutrition rather than as a consequence of (or alongside) overweight/obesity or energy excess. However, the current analysis should be cautiously interpreted for different reasons. First, anemia is multifactorial. Nonnutritional causes of anemia include disease/infection and genetic disorders [10]. To date, population data on micronutrient deficiencies among women are sparse and insufficient to document trends in these [49]. Similarly, isolating the contributions of changes in nutritional and nonnutritional causes of anemia, which could help us better understand the coexistence of overweight/obesity and anemia within individuals, is not possible with the available data [9]. Second, BMI does not accurately reflect body fat composition or the changes that occur with age, which can lead to misclassification errors [50]. Third, the lack of comprehensive data remains an important limitation. We only found 33 countries with at least 2 surveys with data on the outcomes of interest, and therefore, included in this study, and only 9 countries located in the African (*n* for West and Central Africa = 6; *n* for Eastern and Southern Africa = 1), Eastern Mediterranean (*n* = 1), and Southeast Asian (*n* = 1) regions had a survey in the last 5 years. Moreover, data was mainly from the African region, and thus, other regions included in this study are underrepresented, which may have influenced our estimates at the regional or global level. Fourth, for some countries, stratified trend estimates by education level could not be calculated because sample sizes were below 25 participants [15]. Fifth, we compared trends in the co-occurrence of overweight/obesity and anemia and the co-occurrence of anemia and normal weight or underweight. Further stratifying normal weight and underweight into 2 separate groups would have better elucidated how anemia has progressed over time for the 3 different BMI categories. Sixth, our results do not take into account the COVID-19 pandemic, which has likely worsened all forms of malnutrition due to disruptions in essential nutrition interventions and global food systems.

Despite the above-mentioned limitations, investigating the double burden of overweight/obesity and anemia is particularly relevant among women due to the detrimental consequences for themselves, as well as intergenerational (Figure 4). Nutritional anemias can increase the risk and severity of NCDs for which overweight/obesity is already a risk factor, through different pathways [51]. Moreover, reducing anemia in women living with overweight/obesity might prove particularly challenging due to obesity-mediated inflammation, which might impede iron absorption (or other micronutrients) from the diet and reduce country-level efforts to control iron deficiency in these groups [31,36,37,40,52]. Likewise, anemia, as well as overweight/obesity, could lead to poor exercise capacity, contributing further to the development of both conditions. Maternal pregestational overweight/obesity has been hypothesized to increase the risk of childhood anemia [[53], [54], [55]]. A recent prospective study that followed up pregnant women and their infants to 6 mo of age from 3 different countries (Switzerland, Mexico, and Thailand) found that, when compared to pregnant women with normal weight, those with overweight/obesity showed impaired iron absorption in late pregnancy, transferred less iron to their fetuses, and their infants had lower body iron stores [56]. Likewise, maternal obesity increases the risk of fetal macrosomia, which in turn, may lead to inflammation, a rise in hepcidin levels, and over time, result in anemia of inflammation [55,57]. The intergenerational consequences of women with co-occurrent overweight/obesity and anemia during pregnancy are likely to be similar (or perhaps more severe) than those observed among women living with overweight/obesity only [58]. Alternatively, undernutrition during pregnancy (including anemia or micronutrient deficiencies) can result in low birth weight [[59], [60], [61]]. Children who experience undernutrition early on are at a higher risk of developing overweight/obesity later in life, particularly in the context of obesogenic environments and the rapid nutrition transition [[62], [63], [64]].

In conclusion, the analysis of trends and inequalities among adult women suggests a modest increase in the co-occurrence of overweight/obesity and anemia, in parallel with overall reductions in anemia and increases in overweight/obesity. A positive finding is that rates of anemia among women living with underweight and normal weight seem to have dropped between 2000 and 2019 for most countries included in the analysis. In this context, prevention and management early on of overweight/obesity in affected populations might be crucial to address the double burden and anemia altogether. Thus, a shift toward double-duty actions (i.e., interventions, programs, and policies that have the potential to simultaneously reduce the risk or burden of undernutrition and overweight/obesity) is warranted [65]. These could include: *1*) promotion of and support for healthy diets through the life course and from an early age (e.g., promotion of exclusive breastfeeding and appropriate complementary feeding), so that girls become healthy adult women free of malnutrition; *2*) scaling up antenatal care recommendations that include counseling about healthy eating and keeping physically active during pregnancy to stay healthy and prevent excessive weight gain, alongside micronutrient supplementation as recommended; *3*) use of social safety nets (e.g., food transfers, subsidies, and vouchers) as needed per context that exclude energy-dense nutrient-poor foods and reward consumption of nutritious foods; and *4*) implementation of policies to transform food systems that are conducive of healthier food environments and supportive of healthier diets and food choices for all.

## Funding

AI and PG were supported by the National Institute for Health Research (NIHR) Global Health Research Unit on Improving Health in Slums at the University of Warwick (16/136/87).The funding source had no role in the design and conduct of the study; collection, management, analysis, and interpretation of the data; preparation, review, or approval of the manuscript; and decision to submit the manuscript for publication.

## Author disclosures

PG is NIHR Senior Investigator. The views expressed in this publication are those of the authors and not necessarily those of the NIHR or the UK Department of Health and Social Care. All other authors declare no competing interests.

## Data Availability

Data from the Demographic and Health Surveys are publicly available and can be accessed from: https://dhsprogram.com
