# Supplementary material for: Trends in the Intraindividual Double Burden of Overweight/Obesity and Anemia among Adult Women Living in 33 Low- and Middle-Income Countries: A Secondary Analysis of Demographic and Health Surveys from 2000-2019
Source: J Nutr. 2023 Feb 15;153(4):1111–21. doi: 10.1016/j.tjnut.2023.02.012 (PMC10196605; doi:10.1016/j.tjnut.2023.02.012)

**Trends in the intra-individual double burden of overweight/obesity and anemia among adult women living in 33 low- and middle-income countries: a secondary analysis of Demographic and Health Surveys from 2000-2019**

Ana Irache et al.

**Online Supplementary Material**

**(Supplemental Figures 1-10)**

**Supplemental Figure 1.** Flowchart showing participants included in the analysis.


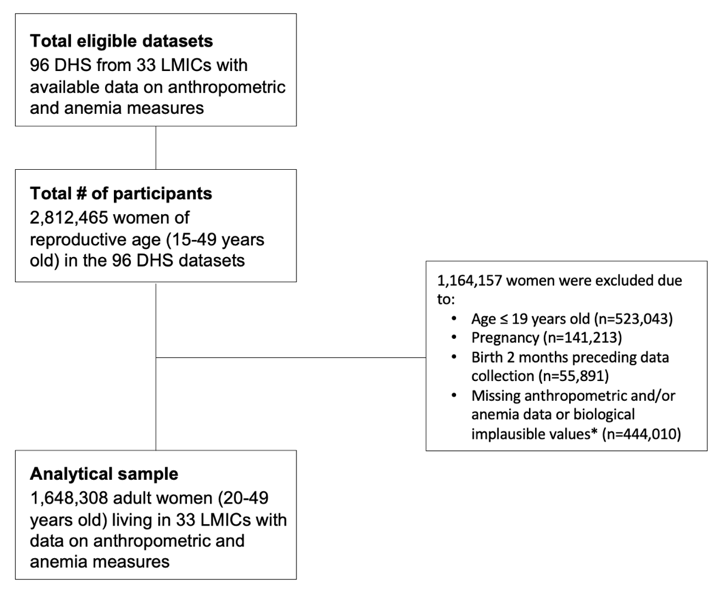


*Note: Height and weight values outside of the ranges 100-220 cm and 20-220kg, respectively, were set as missing values, as were haemoglobin concentrations outside of the 4.0-18.0 g/dL range.

**Supplemental Figure 2.** Equiplots showing changes over time in co-occurrent overweight/obesity and anemia among adult women (20-49 years old) by wealth quintile.


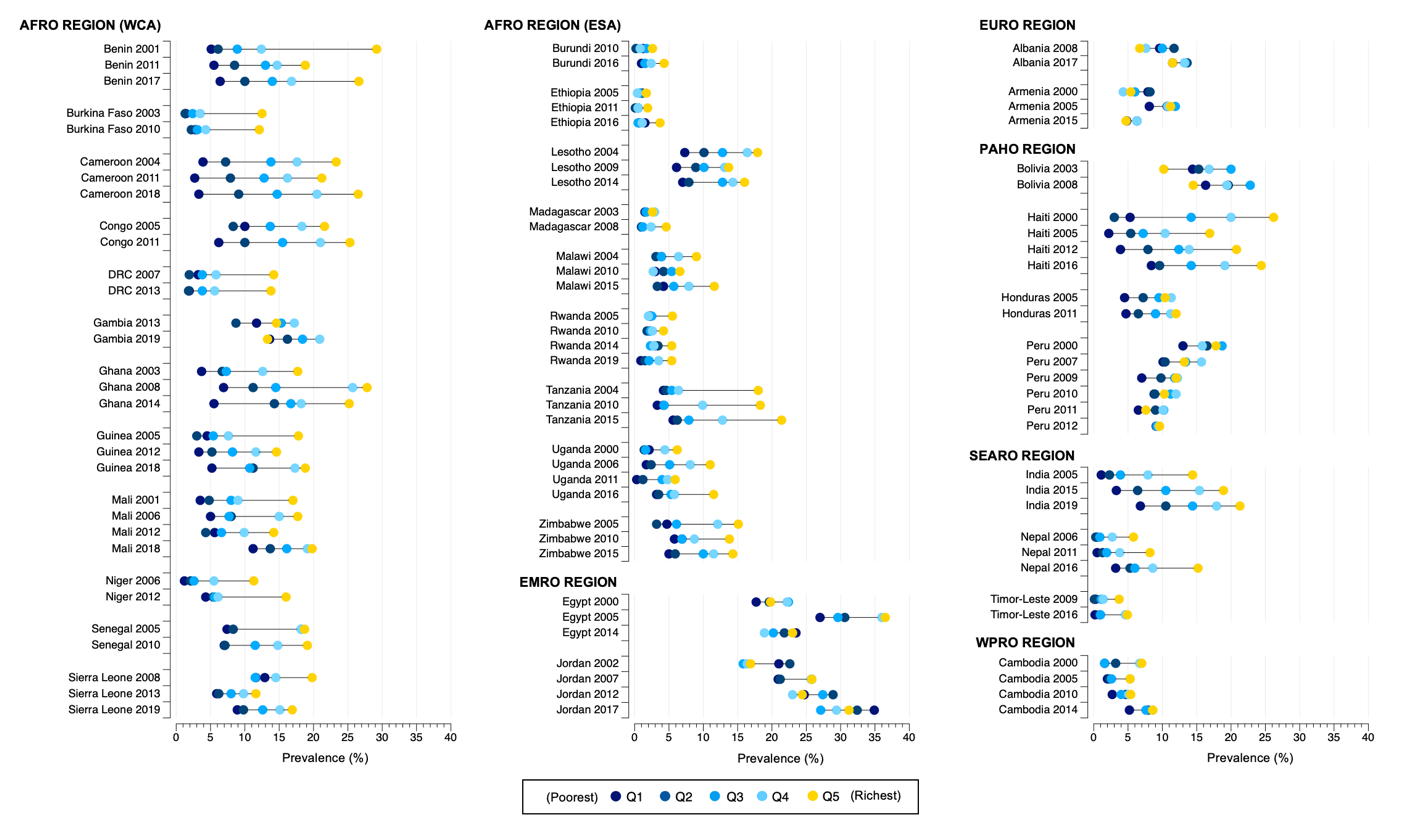


**Supplemental Figure 3.** Equiplots showing changes over time in co-occurrent overweight/obesity and anemia among adult women (20-49 years old) by education level.


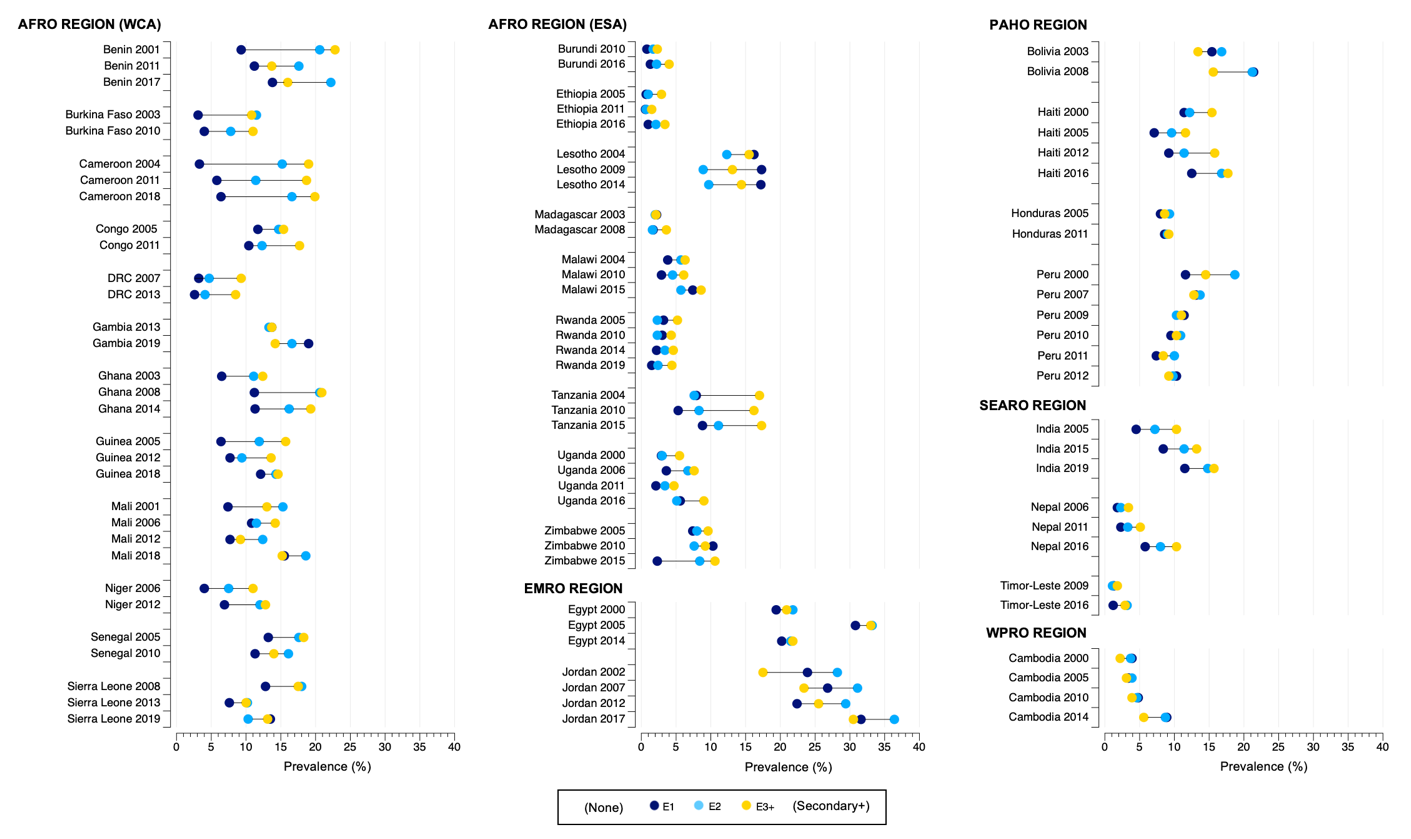


**Supplemental Figure 4.** Equiplots showing changes over time in co-occurrent overweight/obesity and anemia among adult women (20-49 years old) by area of residence.


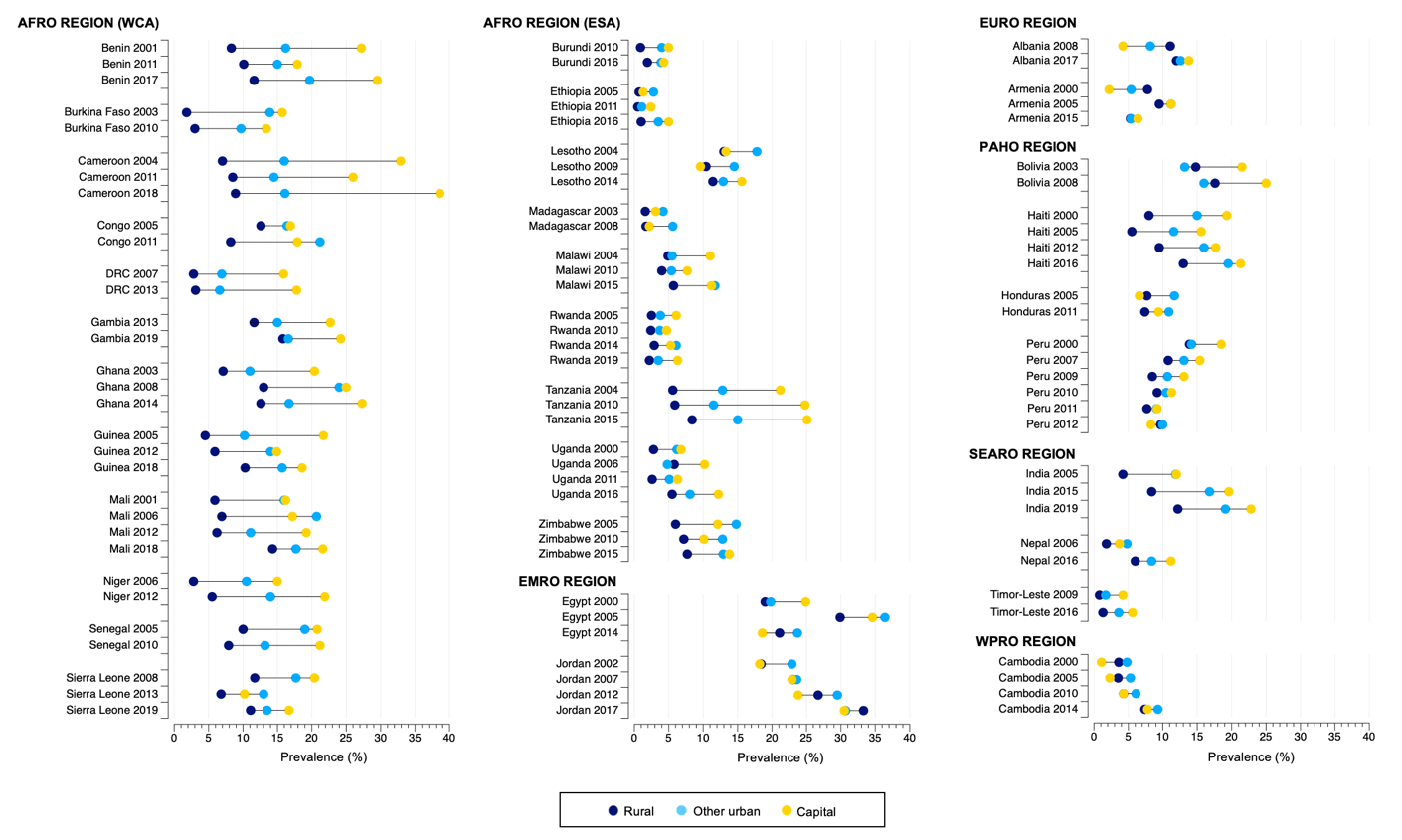


**Supplemental Figure 5.** Trends in the prevalence of co-occurrent overweight/obesity and anemia overall (A) and by household wealth (B), education level (C) and area of residence (D) among adult women (20-49 years old) living in the African region.


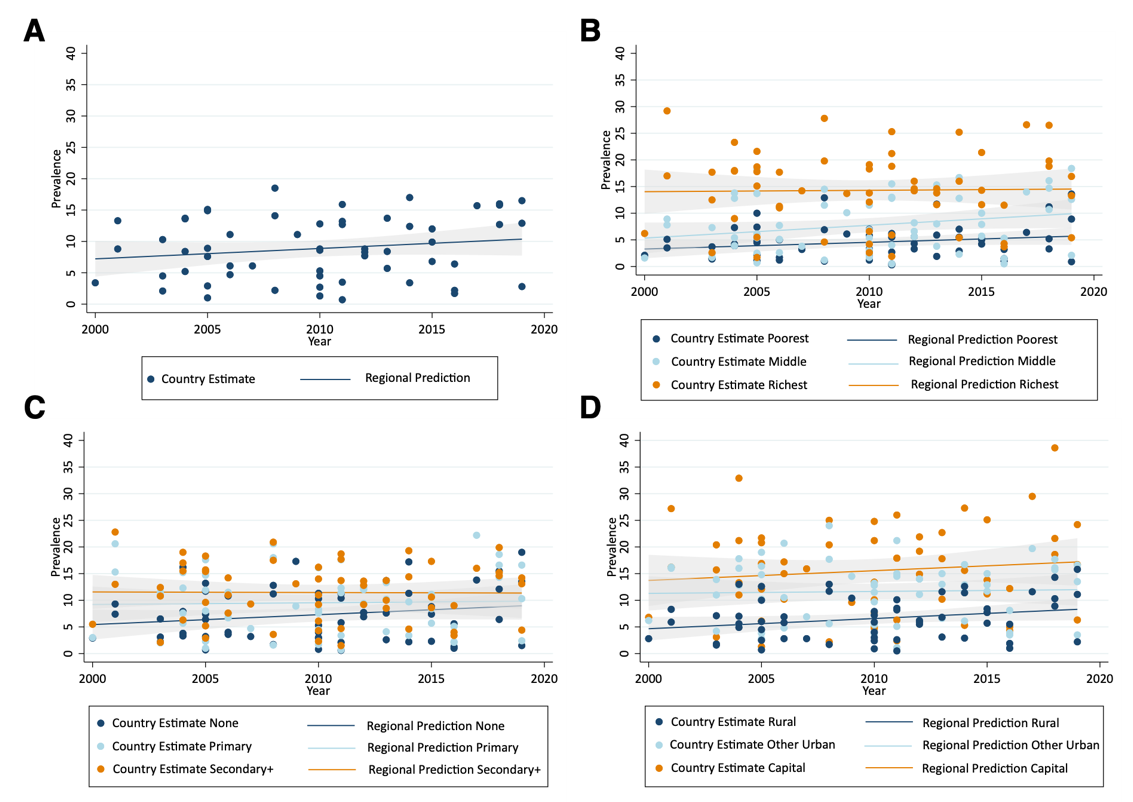


**Supplemental Figure 6.** Trends in the prevalence of co-occurrent overweight/obesity and anemia overall (A) and by household wealth (B), education level (C) and area of residence (D) among adult women (20-49 years old) living in the Eastern Mediterranean region.


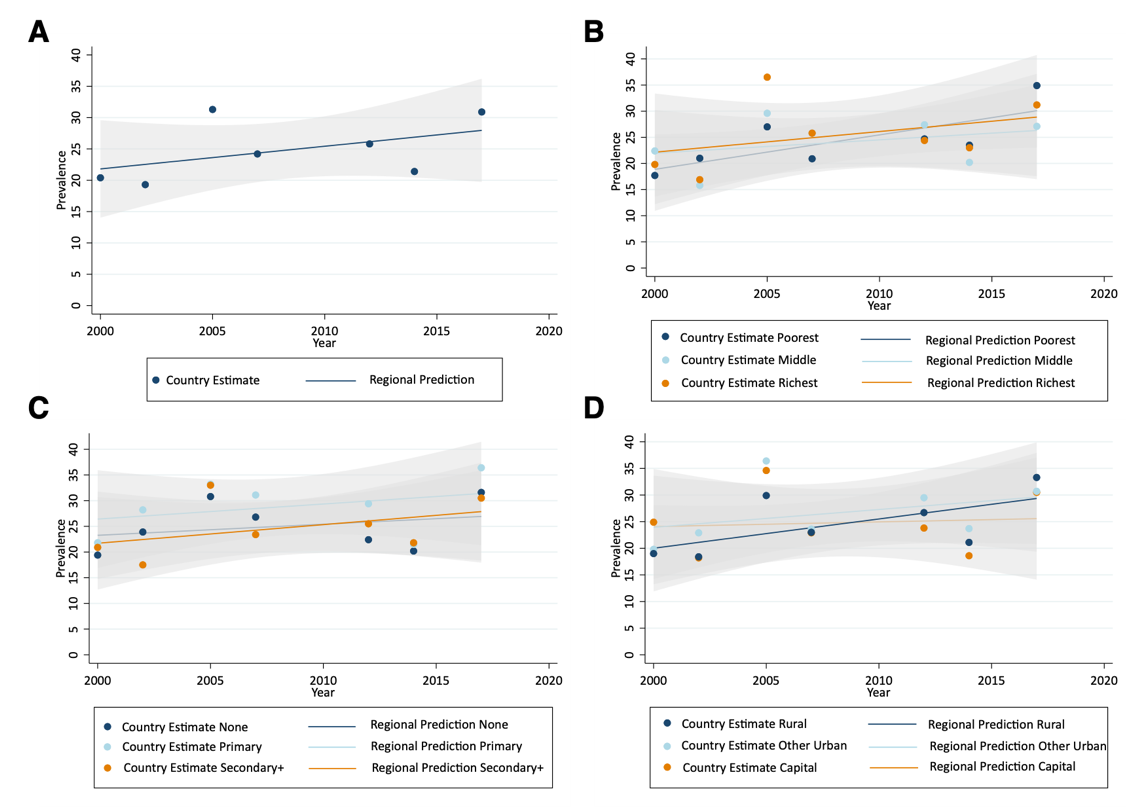


**Supplemental Figure 7.** Trends in the prevalence of co-occurrent overweight/obesity and anemia overall (A) and by household wealth (B) and area of residence (C) among adult women (20-49 years old) living in the European region.


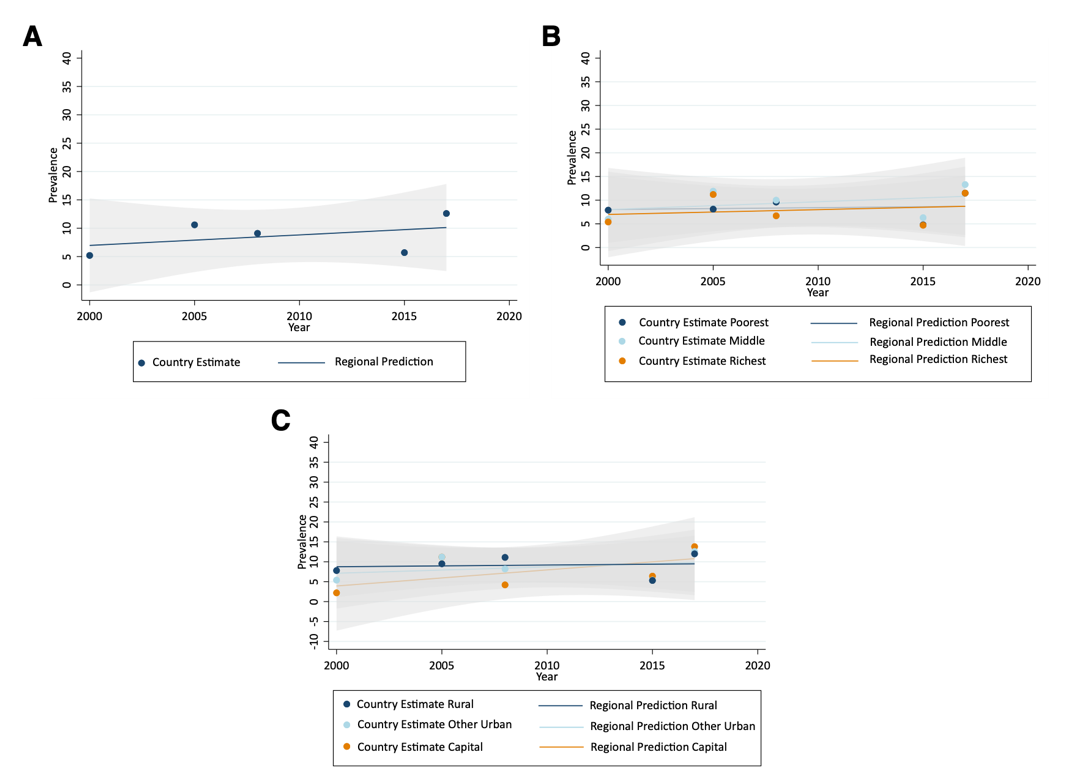


**Supplemental Figure 8.** Trends in the prevalence of co-occurrent overweight/obesity and anemia overall (A) and by household wealth (B), education level (C) and area of residence (D) among adult women (20-49 years old) living in the Americas region.


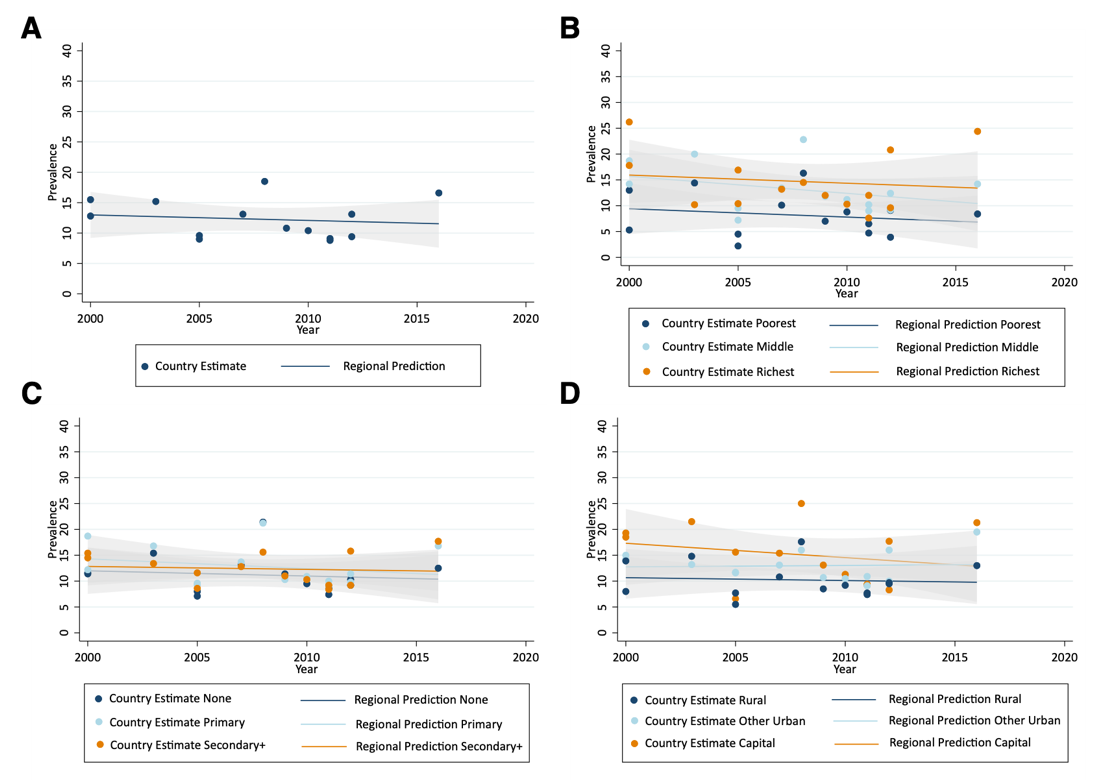


**Supplemental Figure 9.** Trends in the prevalence of co-occurrent overweight/obesity and anemia overall (A) and by household wealth (B), education level (C) and area of residence (D) among adult women (20-49 years old) living in the Southeast Asian region.


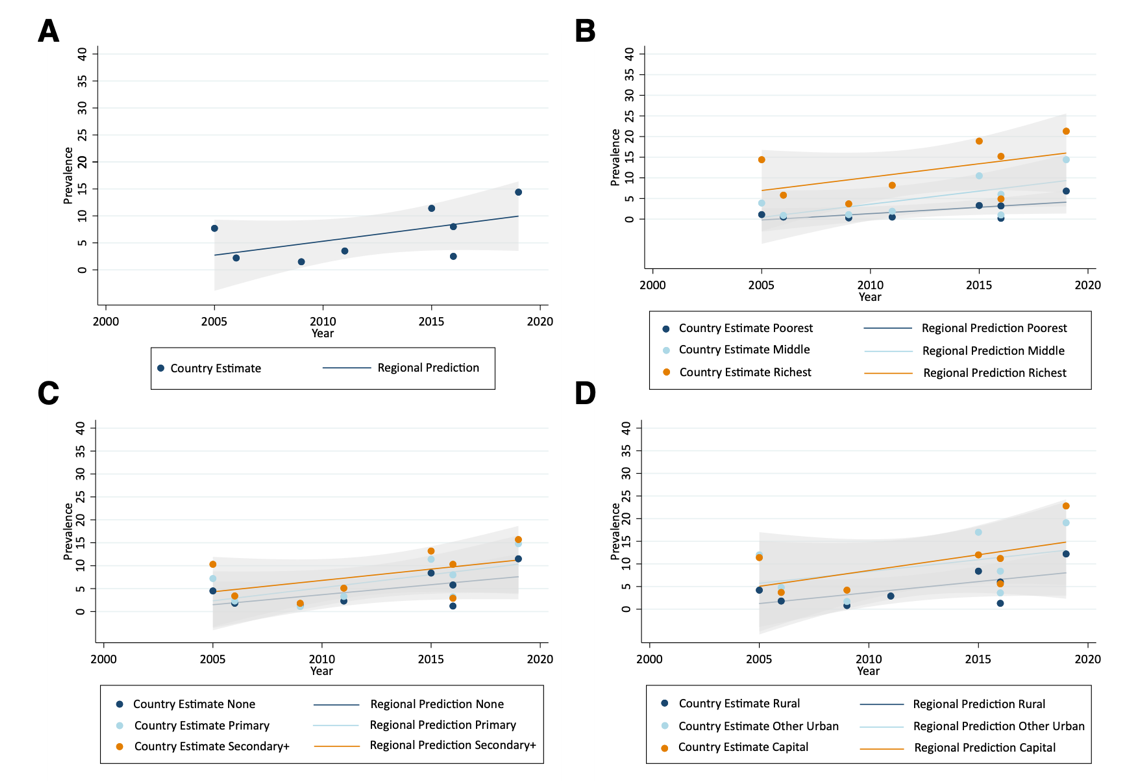


**Supplemental Figure 10.** Trends in the prevalence of co-occurrent overweight/obesity and anemia overall (A) and by household wealth (B), education level (C) and area of residence (D) among adult women (20-49 years old) living in the Western Pacific region.


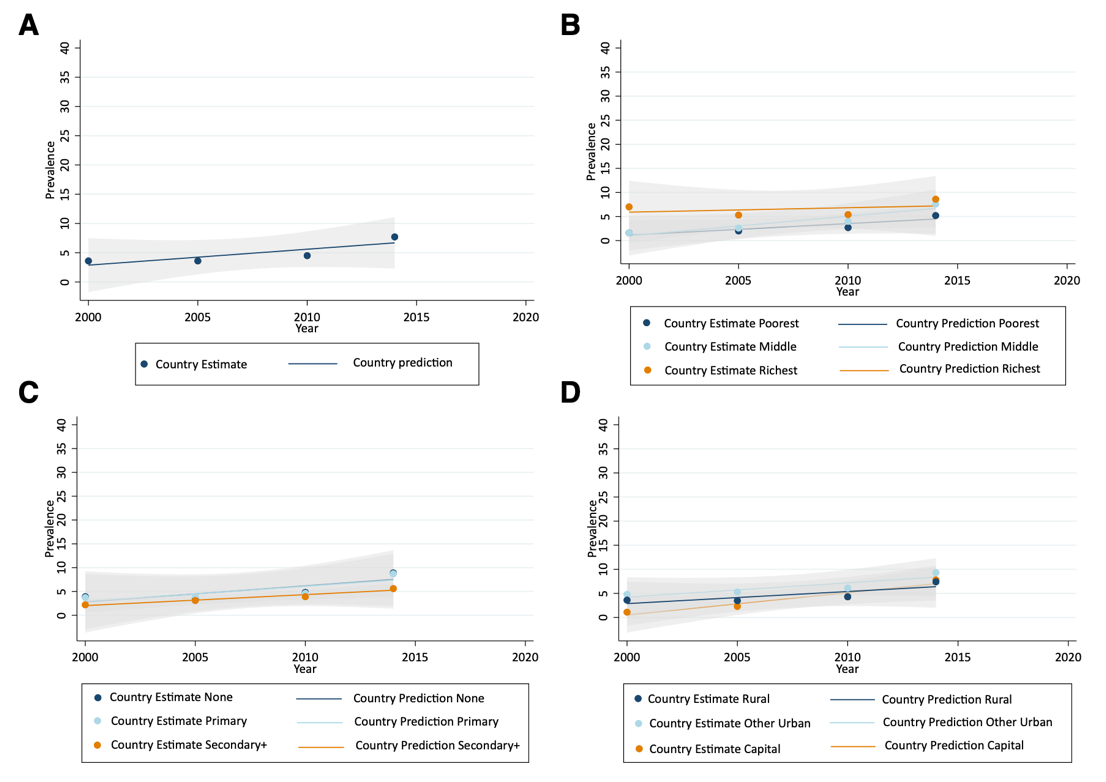

Supplement: Multimedia component 1 [file mmc1.docx]
