# Supplementary material for: Trends in the Intraindividual Double Burden of Overweight/Obesity and Anemia among Adult Women Living in 33 Low- and Middle-Income Countries: A Secondary Analysis of Demographic and Health Surveys from 2000-2019
Source: J Nutr. 2023 Feb 15;153(4):1111–21. doi: 10.1016/j.tjnut.2023.02.012 (PMC10196605; doi:10.1016/j.tjnut.2023.02.012)
Supplement: Multimedia component 2 [file mmc2.docx]

**Trends in the intra-individual double burden of overweight/obesity and anemia among adult women living in 33 low- and middle-income countries: a secondary analysis of Demographic and Health Surveys from 2000-2019**

Ana Irache et al.

**Online Supplementary Material**

**(Supplemental Tables 1-8)**

**Supplemental Table 1.** Databases publicly available in the Demographic and Health Surveys webpage with anthropometric and anemia data among adult women (20-49 years old).

| **Countries in DHS**  (n=83 countries; 397 databases) | **At least 1 DHS available meeting the inclusion criteria**  (n=51 countries; 114 databases) | **>1 DHS available meeting the inclusion criteria; thus, included in this study** (n=33 countries; 96 databases) |
| --- | --- | --- |
| **AFRICAN REGION** | | |
| Angola (n=3) | - | - |
| Benin (n=5) | (n=3; 2001, 2011, 2017) | (n=3; 2001, 2011, 2017) |
| Burkina Faso (n=6) | (n=2; 2003, 2010) | (n=2; 2003, 2010) |
| Burundi (n=4) | (n=2; 2010, 2016) | (n=2; 2010, 2016) |
| Cameroon (n=5) | (n=3; 2004, 2011, 2018) | (n=3; 2004, 2011, 2018) |
| Central African Republic (n=1) | - | - |
| Chad (n=3) | - | - |
| Comoros (n=2) | - | - |
| Congo (n=3) | (n=2; 2005, 2011) | (n=2; 2005, 2011) |
| Cote d’Ivoire (n=4) | (n=1; 2011) | - |
| Democratic Republic of the Congo (n=3) | (n=2; 2007, 2013) | (n=2; 2007, 2013) |
| Eswatini (n=1) | (n=1; 2006) | - |
| Ethiopia (n=5) | (n=3; 2005, 2011, 2016) | (n=3; 2005, 2011, 2016) |
| Gabon (n=2) | (n=1; 2012) | - |
| Gambia (n=2) | (n=2; 2013, 2019) | (n=2; 2013, 2019) |
| Ghana (n=11) | (n=3; 2003, 2008, 2014) | (n=3; 2003, 2008, 2014) |
| Guinea (n=5) | (n=3; 2005, 2012, 2018) | (n=3; 2005, 2012, 2018) |
| Kenya (n=11) | - | - |
| Lesotho (n=3) | (n=3; 2004, 2009, 2014) | (n=3; 2004, 2009, 2014) |
| Liberia (n=7) | - | - |
| Madagascar (n=8) | (n=2; 2003, 2008) | (n=2; 2003, 2008) |
| Malawi (n=10) | (n=3; 2004, 2010, 2015) | (n=3; 2004, 2010, 2015) |
| Mali (n=9) | (n=4; 2001, 2006, 2012, 2018) | (n=4; 2001, 2006, 2012, 2018) |
| Mauritania (n=1) | - | - |
| Mozambique (n=6) | (n=1; 2011) | - |
| Namibia (n=5) | (n=1; 2013) | - |
| Niger (n=4) | (n=2; 2006, 2012) | (n=2; 2006, 2012) |
| Nigeria (n=8) | (n=1; 2018) | - |
| Rwanda (n=12) | (n=4; 2005, 2010, 2014, 2019) | (n=4; 2005, 2010, 2014, 2019) |
| Sao Tome and Principe (n=1) | (n=1; 2008) | - |
| Senegal (n=23) | (n=2; 2005, 2010) | (n=2; 2005, 2010) |
| Sierra Leone (n=4) | (n=3; 2008, 2013, 2019) | (n=3; 2008, 2013, 2019) |
| South Africa (n=2) | (n=1; 2016) | - |
| Tanzania (n=13) | (n=3; 2004, 2010, 2015) | (n=3; 2004, 2010, 2015) |

**Supplemental Table 1.** (continued)

| **Countries in DHS**  (n=83 countries; 397 databases) | **At least 1 DHS available meeting the inclusion criteria**  (n=51 countries; 114 databases) | **>1 DHS available meeting the inclusion criteria; thus, included in this study** (n=33 countries; 96 databases) |
| --- | --- | --- |
| **AFRICAN REGION** | | |
| Togo (n=4) | (n=1; 2013) | - |
| Uganda (n=12) | (n=4; 2000, 2006, 2011, 2016) | (n=4; 2000, 2006, 2011, 2016) |
| Zambia (n=7) | - | - |
| Zimbabwe (n=6) | (n=3; 2005, 2010, 2015) | (n=3; 2005, 2010, 2015) |
| **EASTERN MEDITERRANEAN REGION** | | |
| Afghanistan (n=3) | - | - |
| Egypt (n=12) | (n=3; 2000, 2005, 2014) | (n=3; 2000, 2005, 2014) |
| Jordan (n=7) | (n=4; 2002, 2007, 2012, 2017) | (n=4; 2002, 2007, 2012, 2017) |
| Morocco (n=4) | - | - |
| Pakistan (n=5) | - | - |
| Sudan (n=1) | - | - |
| Tunisia (n=1) | - | - |
| Yemen (n=2) | (n=1; 2013) | - |
| **EUROPEAN REGION** | | |
| Albania (n=2) | (n=2; 2008, 2017) | (n=2; 2008, 2017) |
| Armenia (n=4) | (n=3; 2000, 2005, 2015) | (n=3; 2000, 2005, 2015) |
| Azerbaijan (n=1) | (n=1; 2006) | - |
| Kazakhstan (n=2) | - | - |
| Kyrgyz Republic (n=2) | (n=1; 2012) | - |
| Moldova (n=1) | (n=1; 2005) | - |
| Tajikistan (n=2) | (n=1; 2017) | - |
| Turkiye (n=5) | - | - |
| Ukraine (n=1) | - | - |
| Uzbekistan (n=2) | - | - |
| **AMERICAS REGION** | | |
| Bolivia (n=5) | (n=2; 2003, 2008) | (n=2; 2003, 2008) |
| Brazil (n=3) | - | - |
| Colombia (n=7) | - | - |
| Dominican Republic (n=10) | - | - |
| Ecuador (n=1) | - | - |
| El Salvador (n=1) | - | - |
| Guatemala (n=5) | (n=1; 2014) | - |
| Guyana (n=3) | (n=1; 2009) | - |
| Haiti (n=7) | (n=4; 2000, 2005, 2012, 2016) | (n=4; 2000, 2005, 2012, 2016) |
| Honduras (n=2) | (n=2; 2005, 2011) | (n=2; 2005, 2011) |
| Mexico (n=1) | - | - |

**Supplemental Table 1.** (continued)

| **Countries in DHS**  (n=83 countries; 397 databases) | **At least 1 DHS available meeting the inclusion criteria**  (n=51 countries; 114 databases) | **>1 DHS available meeting the inclusion criteria; thus, included in this study** (n=33 countries; 96 databases) |
| --- | --- | --- |
| **AMERICAS REGION** | | |
| Nicaragua (n=2) | - | - |
| Paraguay (n=1) | - | - |
| Peru (n=11) | (n=6; 2000, 2007, 2009, 2010, 2011, 2012) | (n=6; 2000, 2007, 2009, 2010, 2011, 2012) |
| Trinidad and Tobago (n=1) | - | - |
| **SOUTHEAST ASIAN REGION** | | |
| Bangladesh (n=12) | - | - |
| India (n=5) | (n=3; 2005, 2015, 2019) | (n=3; 2005, 2015, 2019) |
| Indonesia (n=12) | - | - |
| Maldives (n=2) | (n=1; 2016) | - |
| Myanmar (n=1) | (n=1; 2015) | - |
| Nepal (n=8) | (n=3; 2006, 2011, 2016) | (n=3; 2006, 2011, 2016) |
| Sri Lanka (n=1) | - | - |
| Thailand (n=1) | - | - |
| Timor-Leste (n=2) | (n=2; 2009, 2016) | (n=2; 2009, 2016) |
| **WESTERN PACIFIC REGION** | | |
| Cambodia (n=5) | (n=4; 2000, 2005, 2010, 2014) | (n=4; 2000, 2005, 2010, 2014) |
| Philippines (n=7) | - | - |
| Vietnam (n=3) | - | - |

**Supplemental Table 2.** Classification of countries by WHO region and definition of “capital city” for every LMIC included in the analysis.

| **Country** | **Capital** | **Type** |
| --- | --- | --- |
| **AFRICAN REGION** | | |
| Benin | Cotonou | largest/economic |
| Burkina Faso | Ouagadougou | largest/capital |
| Burundi | Bujumbura | largest/economic |
| Cameroon | Douala | largest/economic |
| Congo | Brazzaville | largest/capital |
| Democratic Republic of the Congo (DRC) | Kinshasa | largest/capital |
| Ethiopia | Addis Ababa | largest/capital |
| Gambia | Banjul | largest/capital |
| Ghana | Accra | largest/capital |
| Guinea | Conakry | largest/capital |
| Lesotho | Maseru | largest/capital |
| Madagascar | Antananarivo | largest/capital |
| Malawi | Lilongwe | largest/capital |
| Mali | Bamako | largest/capital |
| Niger | Niamey | largest/capital |
| Rwanda | Kigali | largest/capital |
| Senegal | Dakar | largest/capital |
| Sierra Leone | Freetown | largest/capital |
| Tanzania | Dar es Salaam | largest/economic |
| Uganda | Kampala | largest/capital |
| Zimbabwe | Harare | largest/capital |
| **EASTERN MEDITERRANEAN REGION** | | |
| Egypt | Cairo | largest/capital |
| Jordan | Amman | largest/capital |
| **EUROPEAN REGION** | | |
| Albania | Tirana | largest/capital |
| Armenia | Yerevan | largest/capital |
| **AMERICAS REGION** | | |
| Bolivia | La Paz | capital |
| Haiti | Port-au-Prince | largest/capital |
| Honduras | Tegucigalpa | largest/capital |
| Peru | Lima | largest/capital |
| **SOUTHEAST ASIAN REGION** | | |
| India | New Delhi | capital |
| Nepal | Kathmandu | largest/capital |
| Timor-Leste | Dili | largest/capital |
| **WESTERN PACIFIC REGION** | | |
| Cambodia | Phnom Penh | largest/capital |

| **Supplemental Table 3.** Data used to compute figure 1: prevalence estimates and AARC values in DBM, overweight/obesity only, anemia only and no overweight/obesity or anemia^1^ | **No OWOB or anemia % (95% CIs)** | **AFRICAN REGION** | 28.5 [26.5, 30.7] | 40.1 [38.3, 42.0] | 28.8 [27.3, 30.4] | 0.14 | 42.4 [40.5, 44.3] | 44.9 [43.2, 46.6] | 0.34 | 75.3 [73.2, 77.2] | 54.3 [52.6, 55.9] | -3.50 | 38.7 [37.0, 40.4] | 38.4 [36.6, 40.3] | 34.6 [32.6, 36.7] | -0.29 | 29.2 [26.9, 31.7] | 31.0 [28.7, 33.4] | 0.30 | 41.9 [40.1, 43.7] | 50.4 [48.2, 52.6] | 1.42 | 70.8 [68.5, 72.9] | 77.4 [75.8, 79.0] | 69.4 [67.5, 71.2] | -0.09 | 27.9 [25.3, 30.6] | 30.0 [27.8, 32.2] | 0.35 |
| --- | --- | --- | --- | --- | --- | --- | --- | --- | --- | --- | --- | --- | --- | --- | --- | --- | --- | --- | --- | --- | --- | --- | --- | --- | --- | --- | --- | --- | --- |
|  | **Anemia only %**  **(95% CIs)** |  | 50.0 [47.3, 52.7] | 28.4 [26.7, 30.1] | 40.0 [38.3, 41.7] | -0.78 | 47.7 [45.8, 49.6] | 42.2 [40.4, 44.0] | -0.79 | 16.5 [14.7, 18.5] | 36.9 [35.2, 38.6] | 3.40 | 28.8 [27.1, 30.6] | 24.5 [22.9, 26.1] | 22.6 [20.9, 24.3] | -0.44 | 40.3 [37.2, 43.5] | 37.8 [35.6, 40.1] | -0.42 | 44.1 [42.3, 45.9] | 31.2 [28.8, 33.6] | -2.15 | 24.7 [22.8, 26.7] | 15.9 [14.5, 17.4] | 21.9 [20.2, 23.8] | -0.29 | 45.5 [49.2, 48.2] | 26.3 [24.2, 28.5] | -3.20 |
|  | **OWOB only %**  **(95% CIs)** |  | 8.2 [6.8, 9.8] | 18.8 [17.3, 20.4] | 15.5 [14.3, 16.8] | 0.52 | 5.4 [4.6, 6.3] | 7.6 [6.7, 8.6] | 0.31 | 7.0 [6.0, 8.0] | 6.7 [5.9, 7.6] | -0.05 | 18.8 [17.1, 20.5] | 23.8 [22.4, 25.3] | 26.9 [25.2, 28.9] | 0.58 | 15.6 [14.8, 17.5] | 15.4 [13.5, 17.5] | -0.03 | 7.9 [0.5, 6.9] | 12.7 [10.8, 14.9] | 0.80 | 3.6 [2.7, 4.7] | 6.0 [5.1, 6.9] | 7.0 [6.0, 8.3] | 0.31 | 12.9 [11.4, 14.5] | 27.3 [25.0, 29.6] | 2.40 |
|  | **DBM %**  **(95% CIs)** |  | 13.3 [11.8, 14.9] | 12.7 [11.4, 14.1] | 15.7 [14.6, 16.9] | 0.13 | 4.5 [3.8, 5.4] | 5.3 [4.7, 6.0] | 0.11 | 1.3 [0.9, 1.8] | 2.2 [1.8, 2.6] | 0.15 | 13.7 [12.6, 15.0] | 13.2 [12.3, 14.4] | 16.0 [14.5, 17.5] | 0.16 | 14.9 [12.9, 17.1] | 15.9 [13.8, 18.1] | 0.17 | 6.1 [5.3, 7.0] | 5.7 [4.9, 6.6] | -0.07 | 1.0 [0.7, 1.4] | 0.7 [0.5, 1.0] | 1.7 [1.3, 2.1] | 0.06 | 13.7 [12.2, 15.3] | 16.5 [14.9, 18.2] | 0.47 |
|  | **N** |  | 2,070 | 3,514 | 5,274 |  | 2,707 | 5,671 |  | 2,794 | 5,675 |  | 3,253 | 5,148 | 4,430 |  | 2,167 | 3,779 |  | 3,021 | 5,930 |  | 3,921 | 10,317 | 9,878 |  | 2,923 | 3,914 |  |
|  | **Survey year** |  | 2001 | 2011 | 2017 |  | 2003 | 2010 |  | 2010 | 2016 |  | 2004 | 2011 | 2018 |  | 2005 | 2011 |  | 2007 | 2013 |  | 2005 | 2011 | 2016 |  | 2013 | 2019 |  |
|  | **Income^2^** |  | LMI |  |  |  | LI |  |  | LI |  |  | LMI |  |  |  | LMI |  |  | LI |  |  | Li |  |  |  | LI |  |  |
|  | **Country** |  | Benin |  |  | AARC (%-points): | Burkina Faso |  | AARC (%-points): | Burundi |  | AARC (%-points): | Cameroon |  |  | AARC (%-points): | Congo |  | AARC (%-points): | DRC |  | AARC (%-points): | Ethiopia |  |  | AARC (%-points): | Gambia |  | AARC (%-points): |

| **Supplemental Table 3.** (continued)^1^ | **No OWOB or anemia % (95% CIs)** | **AFRICAN REGION** | 37.9 [36.1, 39.8] | 26.4 [24.5, 28.5] | 29.2 [27.3, 31.1] | -0.75 | 40.3 [38.2, 42.4] | 38.7 [36.4, 40.9] | 37.0 [35.0, 39.0] | -0.25 | 34.1 [31.8, 36.6] | 35.7 [33.3, 38.2] | 33.3 [31.0, 35.8] | -0.08 | 51.6 [48.2, 55.0] | 59.2 [57.4, 60.9] | 1.52 | 46.2 [43.7, 48.6] | 56.9 [55.1, 58.6] | 51.7 [49.9, 53.4] | 0.54 | 30.4 [28.2, 32.7] | 29.4 [27.3, 31.6] | 38.1 [36.0, 40.2] | 22.5 [20.6, 24.5] | -0.27 | 47.6 [45.3, 49.9] | 44.8 [42.3, 47.2] | -0.47 |
| --- | --- | --- | --- | --- | --- | --- | --- | --- | --- | --- | --- | --- | --- | --- | --- | --- | --- | --- | --- | --- | --- | --- | --- | --- | --- | --- | --- | --- | --- |
|  | **Anemia only %**  **(95% CIs)** |  | 32.7 [30.7, 34.7] | 38.4 [36.4, 40.6] | 23.7 [21.8, 25.7] | -0.87 | 43.3 [41.3, 45.4] | 38.5 [36.0, 41.1] | 31.5 [29.5, 33.5] | -0.90 | 19.1 [17.0, 21.3] | 15.6 [14.0, 17.3] | 14.8 [13.2, 16.6] | -0.43 | 40.7 [37.3, 44.3] | 33.1 [31.4, 34.9] | -1.52 | 38.0 [35.5, 40.6] | 23.1 [21.5, 24.7] | 23.8 [22.4, 25.2] | -1.33 | 52.7 [50.1, 55.3] | 48.4 [44.7, 52.1] | 41.3 [39.3, 43.4] | 46.0 [43.4, 48.6] | -0.47 | 36.7 [34.3, 39.2] | 34.5 [32.1, 37.0] | -0.37 |
|  | **OWOB only %**  **(95% CIs)** |  | 19.1 [17.5, 20.8] | 16.7 [15.1, 18.4] | 30.1 [28.1, 32.2] | 1.04 | 8.8 [7.6, 10.1] | 14.0 [12.2, 15.9] | 18.9 [17.3, 20.5] | 0.78 | 33.2 [30.6, 36.0] | 37.7 [35.4, 40.1] | 39.5 [17.4, 41.7] | 0.63 | 5.6 [4.5, 6.9] | 5.5 [4.7, 6.5] | -0.02 | 10.6 [9.1, 12.3] | 15.5 [14.2, 17.0] | 17.8 [16.6, 19.2] | 0.66 | 8.1 [6.8, 9.7] | 11.1 [9.8, 12.6] | 12.3 [11.0, 13.8] | 15.7 [13.9, 17.6] | 0.42 | 11.0 [9.7, 12.4] | 13.1 [11.7, 14.6] | 0.35 |
|  | **DBM %**  **(95% CIs)** |  | 10.3 [9.2, 11.6] | 18.5 [17.0, 20.1] | 17.0 [15.2, 19.0] | 0.58 | 7.6 [6.5, 9.0] | 8.8 [7.8, 10.1] | 12.7 [11.4, 14.1] | 0.38 | 13.6 [11.9, 15.4] | 11.1 [9.6, 12.8] | 12.4 [10.9, 14.1] | -0.12 | 2.1 [1.6, 2.8] | 2.2 [1.8, 2.7] | 0.02 | 5.2 [4.1, 6.6] | 4.5 [3.9, 5.3] | 6.8 [6.0, 7.6] | 0.14 | 8.8 [7.2, 10.6] | 11.1 [8.6, 14.4] | 8.3 [7.3, 9.3] | 15.8 [14.3, 17.4] | 0.32 | 4.7 [3.9, 5.8] | 7.7 [6.6, 8.9] | 0.50 |
|  | **N** |  | 3,620 | 3,276 | 3,363 |  | 2,577 | 3,065 | 3,439 |  | 2,048 | 2,732 | 2,383 |  | 1,802 | 5,585 |  | 1,779 | 4,817 | 5,553 |  | 2,275 | 2,976 | 3,505 | 3,375 |  | 2,758 | 3,303 |  |
|  | **Survey year** |  | 2003 | 2008 | 2014 |  | 2005 | 2012 | 2018 |  | 2004 | 2009 | 2014 |  | 2003 | 2008 |  | 2004 | 2010 | 2015 |  | 2001 | 2006 | 2012 | 2018 |  | 2006 | 2012 |  |
|  | **Income^2^** |  | LMI |  |  |  | LI |  |  |  | LMI |  |  |  | LI |  |  | LI |  |  |  | LI |  |  |  |  | LI |  |  |
|  | **Country** |  | Ghana |  |  | AARC (%-points): | Guinea |  |  | AARC (%-points): | Lesotho |  |  | AARC (%-points): | Madagascar |  | AARC (%-points): | Malawi |  |  | AARC (%-points): | Mali |  |  |  | AARC (%-points): | Niger |  | AARC (%-points): |

| **Supplemental Table 3.** (continued)^1^ | **No OWOB or anemia % (95% CIs)** | **AFRICAN REGION** | 65.0 [63.2, 66.9] | 67.5 [66.2, 68.8] | 61.9 [60.4, 63.5] | 61.6 [59.9, 63.2] | -0.33 | 30.2 [28.2, 32.3] | 33.6 [31.6, 35.7] | 0.68 | 38.9 [36.4, 41.6] | 44.9 [42.5, 47.3] | 35.8 [34.2, 37.5] | -0.32 | 42.3 [40.4, 44.2] | 46.5 [44.7, 48.3] | 36.2 [34.8, 37.7] | -0.51 | 52.7 [50.9, 54.6] | 46.2 [43.5, 49.0] | 59.1 [56.7, 61.5] | 49.0 [47.1, 51.0] | 0.03 | 42.9 [41.3, 44.5] | 44.2 [42.7, 45.7] | 42.4 [40.9, 44.0] | -0.05 |
| --- | --- | --- | --- | --- | --- | --- | --- | --- | --- | --- | --- | --- | --- | --- | --- | --- | --- | --- | --- | --- | --- | --- | --- | --- | --- | --- | --- |
|  | **Anemia only %**  **(95% CIs)** |  | 22.9 [21.3, 24.5] | 14.6 [13.6, 15.7] | 15.2 [13.9, 16.5] | 8.7 [7.8, 9.7] | -0.93 | 42.0 [39.2, 44.9] | 40.6 [17.7, 43.6] | -0.28 | 29.7 [27.3, 32.3] | 33.5 [31.1, 36.0] | 31.6 [29.6, 33.7] | 0.16 | 36.9 [35.3, 38.5] | 28.8 [27.3, 30.2] | 30.3 [28.8, 31.8] | -0.62 | 32.5 [30.6, 34.5] | 35.0 [32.2, 37.8] | 19.6 [17.7, 21.6] | 23.1 [21.4, 24.9] | -0.81 | 28.3 [26.2, 30.4] | 19.4 [18.2, 20.8] | 16.4 [15.3, 17.6] | -1.19 |
|  | **OWOB only %**  **(95% CIs)** |  | 9.3 [8.2, 10.4] | 15.2 [14.2, 16.3] | 19.5 [18.3, 20.8] | 26.9 [25.3, 28.5] | 1.24 | 12.8 [11.0, 14.7] | 13.0 [11.5, 14.6] | 0.04 | 17.3 [15.0, 19.8] | 13.2 [11.7, 14.8] | 19.6 [18.2, 21.2] | 0.24 | 12.4 [11.4, 13.4] | 16.2 [14.9, 17.5] | 21.6 [20.2, 23.0] | 0.83 | 11.4 [10.2, 12.6] | 12.7 [10.7, 15.0] | 17.7 [15.9, 19.6] | 21.5 [19.8, 23.1] | 0.66 | 20.0 [18.3, 21.7] | 27.6 [26.2, 29.0] | 31.3 [29.8, 32.9] | 1.13 |
|  | **DBM %**  **(95% CIs)** |  | 2.9 [2.3, 3.5] | 2.7 [2.2, 3.3] | 3.4 [3.0, 4.0] | 2.8 [2.3, 3.4] | 0.01 | 15.1 [13.3, 17.0] | 12.8 [11.1, 14.7] | -0.46 | 14.1 [12.2, 16.2] | 8.4 [7.4, 9.6] | 12.9 [11.9, 14.1] | -0.08 | 8.4 [7.5, 9.5] | 8.6 [7.7, 9.6] | 12.0 [11.1, 13.0] | 0.32 | 3.4 [2.7, 4.1] | 6.1 [4.9, 7.6] | 3.5 [2.7, 4.5] | 6.4 [5.5, 7.5] | 0.12 | 8.9 [8.1, 9.8] | 8.8 [7.9, 9.7] | 9.9 [9.0, 10.9] | 0.10 |
|  | **N** |  | 3,667 | 4,790 | 4,685 | 5,057 |  | 2,752 | 3,614 |  | 2,418 | 5,271 | 5,052 |  | 6,612 | 6,593 | 8,791 |  | 4,096 | 1,765 | 1,693 | 3,868 |  | 5,309 | 5,636 | 6,515 |  |
|  | **Survey year** |  | 2005 | 2010 | 2014 | 2019 |  | 2005 | 2010 |  | 2008 | 2013 | 2019 |  | 2004 | 2010 | 2015 |  | 2000 | 2006 | 2011 | 2016 |  | 2005 | 2010 | 2015 |  |
|  | **Income^2^** |  | LI |  |  |  |  | LMI |  |  | LI |  |  |  | LMI |  |  |  | LI |  |  |  |  | LMI |  |  |  |
|  | **Country** |  | Rwanda |  |  |  | AARC (%-points): | Senegal |  | AARC (%-points): | Sierra Leone |  |  | AARC (%-points): | Tanzania |  |  | AARC (%-points): | Uganda |  |  |  | AARC (%-points): | Zimbabwe |  |  | AARC (%-points): |

| **Supplemental Table 3.** (continued)^1^ | **No OWOB or anemia % (95% CIs)** | **EASTERN MEDITERRANEAN REGION** | 14.2 [13.2, 15.2] | 10.5 [10.4, 10.5] | 10.3 [9.5, 11.3] | -0.25 | 22.6 [19.9, 25.6] | 20.0 [19.9, 20.2] | 16.9 [15.4, 18.6] | 16.3 [14.9, 17.8] | -0.44 | **EUROPEAN REGION** | 43.0 [41.2, 44.9] | 30.1 [28.6, 31.6] | -1.43 | 45.7 [44.1, 47.2] | 38.5 [36.3, 40.7] | 42.9 [41.0, 44.8] | -0.10 | **AMERICAS REGION** | 29.7 [27.9, 31.5] | 25.2 [23.5, 27.0] | -0.90 | 31.4 [28.3, 24.8] | 40.2 [38.1, 42.3] | 35.1 [33.5, 36.7] | 31.1 [29.5, 32.6] | -0.11 |
| --- | --- | --- | --- | --- | --- | --- | --- | --- | --- | --- | --- | --- | --- | --- | --- | --- | --- | --- | --- | --- | --- | --- | --- | --- | --- | --- | --- | --- |
|  | **Anemia only %**  **(95% CIs)** |  | 7.0 [6.3, 7.8] | 7.8 [7.8, 7.8] | 4.3 [3.7, 4.9] | -0.22 | 8.1 [6.5, 10.1] | 12.2 [12.1, 12.2] | 11.1 [9.8, 12.5] | 13.8 [12.5, 15.3] | 0.32 |  | 10.2 [9.2, 11.3] | 9.1 [8.3, 9.9] | -0.12 | 7.2 [6.3, 8.1] | 12.5 [11.1, 14.0] | 7.2 [6.3, 8.2] | -0.08 |  | 16.6 [15.1, 18.3] | 18.1 [16.6, 19.6] | 0.30 | 40.3 [38.2, 42.5] | 33.8 [32.0, 35.6] | 33.8 [32.2, 35.5] | 30.4 [28.9, 31.9] | -0.52 |
|  | **OWOB only %**  **(95% CIs)** |  | 58.4 [56.9, 60.0] | 50.4 [50.4, 50.5] | 64.0 [62.4, 65.6] | 0.53 | 50.0 [47.4, 52.7] | 43.6 [43.6, 43.7] | 46.2 [44.3, 48.2] | 39.0 [36.8, 41.2] | -0.61 |  | 37.8 [8.1, 10.1] | 48.2 [46.8, 49.6] | 1.16 | 42.1 [40.6, 43.5] | 38.5 [36.5, 40.5] | 44.2 [42.5, 46.0] | 0.20 |  | 38.5 [36.5, 40.6] | 38.3 [36.5, 40.1] | -0.04 | 15.5 [13.4, 17.9] | 16.4 [14.9, 18.1] | 18.0 [16.8, 19.4] | 22.0 [20.8, 23.2] | 0.37 |
|  | **DBM %**  **(95% CIs)** |  | 20.4 [19.0, 21.8] | 31.3 [31.3, 31.4] | 21.4 [20.0, 22.8] | -0.07 | 19.3 [16.8, 22.0] | 24.2 [24.1, 24.4] | 25.8 [23.9, 27.7] | 30.9 [29.1, 32.8] | 0.73 |  | 9.1 [8.1, 10.1] | 12.6 [11.8, 13.5] | 0.39 | 5.2 [4.5, 5.9] | 10.6 [9.5, 11.8] | 5.7 [4.8, 6.7] | -0.04 |  | 15.2 [13.7, 16.9] | 18.5 [17.0, 20.1] | 0.66 | 12.8 [10.3, 15.8] | 9.6 [8.3, 11.1] | 13.1 [11.9, 14.4] | 16.6 [15.3, 17.9] | 0.27 |
|  | **N** |  | 6,422 | 5,322 | 6,077 |  | 1,468 | 4,147 | 5,902 | 5,879 |  |  | 5,796 | 12,360 |  | 4,795 | 4,838 | 4,868 |  |  | 4,147 | 4,063 |  | 3,136 | 3,483 | 6,400 | 6,587 |  |
|  | **Survey year** |  | 2000 | 2005 | 2014 |  | 2002 | 2007 | 2012 | 2017 |  |  | 2008 | 2017 |  | 2000 | 2005 | 2015 |  |  | 2003 | 2008 |  | 2000 | 2005 | 2012 | 2016 |  |
|  | **Income^2^** |  | LMI |  |  |  | UMI |  |  |  |  |  | UMI |  |  | UMI |  |  |  |  | LMI |  |  | LMI |  |  |  |  |
|  | **Country** |  | Egypt |  |  | AARC (%-points): | Jordan |  |  |  | AARC (%-points): |  | Albania |  | AARC (%-points): | Armenia |  |  | AARC (%-points): |  | Bolivia |  | AARC (%-points): | Haiti |  |  |  | AARC (%-points): |

| **Supplemental Table 3.** (continued)^1^ | **No OWOB or anemia % (95% CIs)** | **AMERICAS REGION** | 36.8 [35.7, 37.9] | 33.6 [32.7, 34.6] | -0.53 | 30.5 [28.9, 32.1] | 32.5 [31.4, 33.6] | 32.7 [31.7, 33.8] | 31.9 [30.9, 33.0] | 33.0 [32.0, 34.0] | 30.8 [29.8, 31.8] | 0.11 | **SOUTHEAST ASIAN REGION**  **SOUTHEAST ASIAN REGION**  **SOUTHEAST ASIAN REGION** | 37.9 [37.6, 38.3] | 34.3 [34.0, 34.5] | 29.8 [29.8, 30.2] | -0.54 | 57.8 [54.1, 61.3] | 54.2 [52.0, 56.3] | 41.6 [39.4, 43.9] | -1.62 | 74.3 [72.2, 76.2] | 67.3 [65.0, 69.5] | -1.00 | **WESTERN PACIFIC REGION**  **WESTERN PACIFIC REGION**  **WESTERN PACIFIC REGION** | 36.5 [34.4, 38.6] | 45.9 [44.4, 47.5] | 48.3 [46.7, 49.9] | 42.9 [41.5, 44.2] | 0.49 | ^1^Values are prevalence estimates and 95% CIs, unless otherwise indicated. A positive AARC value depicts an increase in %-points in malnutrition over time; whereas a negative value means that the prevalence is decreasing. Abbreviations: AARC,  average annual rate of change; Anemia only, anemia with no overweight/obesity (i.e., anemia with underweight or normal weight); DRC, Democratic Republic of the Congo; DBM, co-occurrent overweight/obesity and anemia; OWOB only,  overweight/obesity (no anemia).  ^2^Latest World Bank Income classification: LI, low-income; LMI, lower-middle-income; UMI, upper-middle-income. |
| --- | --- | --- | --- | --- | --- | --- | --- | --- | --- | --- | --- | --- | --- | --- | --- | --- | --- | --- | --- | --- | --- | --- | --- | --- | --- | --- | --- | --- | --- | --- | --- |
|  | **Anemia only %**  **(95% CIs)** |  | 9.3 [8.6, 10.0] | 6.4 [5.9, 6.9] | -0.48 | 15.2 [13.9, 16.6] | 12.7 [11.9, 13.5] | 9.4 [8.9, 10.1] | 10.5 [9.8, 11.2] | 7.7 [7.1, 8.2] | 7.5 [6.9, 8.0] | -0.65 |  | 43.6 [43.2, 43.9] | 41.5 [41.2, 41.7] | 42.2 [42.0, 42.5] | -0.12 | 31.9 [28.2, 35.7] | 29.5 [27.2, 32.0] | 31.5 [29.4, 33.8] | -0.04 | 18.3 [16.5, 20.2] | 19.2 [17.4, 21.1] | 0.13 |  | 55.0 [52.7, 57.2] | 42.0 [40.6, 43.9] | 38.5 [36.9, 40.1] | 36.1 [34.8, 37.4] | -1.30 |  |
|  | **OWOB only %**  **(95% CIs)** |  | 44.9 [43.8, 46.1] | 50.9 [49.8, 52.0] | 1.00 | 38.8 [36.9, 40.6] | 41.8 [40.6, 43.0] | 47.0 [45.9, 48.1] | 47.2 [46.1, 48.3] | 50.5 [49.5, 51.6] | 52.4 [51.2, 53.5] | 1.10 |  | 10.8 [10.6, 11.0] | 12.9 [12.7, 13.0] | 13.3 [13.2, 13.5] | 0.18 | 8.2 [6.9, 9.7] | 12.9 [11.2, 14.8] | 18.9 [17.0, 20.9] | 1.07 | 6.1 [5.0, 7.3] | 11.1 [9.7, 12.6] | 0.71 |  | 5.0 [4.1, 6.2] | 8.3 [7.2, 9.5] | 8.8 [7.9, 9.8] | 13.4 [12.5, 14.3] | 0.54 |  |
|  | **DBM %**  **(95% CIs)** |  | 9.0 [8.3, 9.7] | 9.1 [8.5, 9.7] | 0.02 | 15.5 [14.3, 16.9] | 13.1 [12.3, 13.8] | 10.8 [10.2, 11.6] | 10.4 [9.8, 11.1] | 8.8 [8.2, 9.4] | 9.4 [8.8, 10.0] | -0.56 |  | 7.7 [7.5, 7.9] | 11.4 [11.2, 11.6] | 14.4 [14.3, 14.6] | 0.46 | 2.2 [1.8, 2.8] | 3.5 [2.8, 4.2] | 8.0 [7.0, 9.2] | 0.58 | 1.5 [0.9, 2.3] | 2.5 [1.9, 3.3] | 0.14 |  | 3.6 [2.8, 4.5] | 3.6 [3.1, 4.2] | 4.5 [3.8, 5.2] | 7.7 [6.9, 8.4] | 0.27 |  |
|  | **N** |  | 12,941 | 15,323 |  | 4,559 | 18,728 | 16,798 | 16,649 | 16,716 | 17,898 |  |  | 84,976 | 524,796 | 538,221 |  | 7,598 | 4,439 | 4,787 |  | 2,696 | 2,870 |  |  | 2,500 | 5,875 | 6,744 | 8,703 |  |  |
|  | **Survey year** |  | 2005 | 2011 |  | 2000 | 2007 | 2009 | 2010 | 2011 | 2012 |  |  | 2005 | 2015 | 2019 |  | 2006 | 2011 | 2016 |  | 2009 | 2016 |  |  | 2000 | 2005 | 2010 | 2014 |  |  |
|  | **Income^2^** |  | LMI |  |  | UMI |  |  |  |  |  |  |  | LMI |  |  |  | LMI |  |  |  | LMI |  |  |  | LMI |  |  |  |  |  |
|  | **Country** |  | Honduras |  | AARC (%-points): | Peru |  |  |  |  |  | AARC (%-points): |  | India |  |  | AARC (%-points): | Nepal |  |  | AARC (%-points): | Timor-Leste |  | AARC (%-points): |  | Cambodia |  |  |  | AARC (%-points): |  |

| **Supplemental Table 4.** National prevalence of co-occurrent overweight/obesity and anemia, overweight/obesity, and anemia among adult women (20-49 years old) in the 96 DHS surveys included in the study and AARC values^1^ | **Anemia % (95% CIs)** | **AFRICAN REGION** | 63.3 [60.8, 65.7] | 41.1 [39.1, 43.1] | 55.7 [54.0, 57.4] | -0.65 | 52.2 [50.3, 54.1] | 47.5 [45.7, 49.3] | -0.67 | 17.8 [15.9, 19.9] | 39.1 [37.4, 40.8] | 3.55 | 42.6 [40.7, 44.5] | 37.8 [36.1, 39.5] | 38.5 [36.5, 40.6] | -0.29 | 55.2 [52.2, 58.2] | 53.7 [51.1, 56.2] | -0.25 | 50.2 [48.4, 52.0] | 36.9 [34.5, 39.4] | -2.22 | 25.7 [23.7, 27.8] | 16.6 [15.2, 18.1] | 23.6 [21.8, 25.4] | -0.23 | 59.2 [56.3, 62.1] | 42.8 [40.0, 45.6] | -2.73 |
| --- | --- | --- | --- | --- | --- | --- | --- | --- | --- | --- | --- | --- | --- | --- | --- | --- | --- | --- | --- | --- | --- | --- | --- | --- | --- | --- | --- | --- | --- |
|  | **OWOB % (95% CIs)** |  | 21.5 [19.3, 23.8] | 31.5 [29.7, 33.3] | 31.2 [29.6, 32.9] | 0.64 | 9.9 [8.8, 11.1] | 12.9 [11.7, 14.1] | 0.43 | 8.2 [7.3, 9.3] | 8.8 [7.9, 9.9] | 0.10 | 32.5 [30.6, 34.4] | 37.1 [35.4, 38.9] | 42.8 [40.7, 45.0] | 0.74 | 30.0 [28.1, 32.0] | 31.2 [28.7, 33.8] | 0.20 | 14.0 [12.8, 15.3] | 18.4 [16.3, 20.8] | 0.73 | 4.6 [3.6, 5.7] | 6.7 [5.8, 7.8] | 8.7 [7.5, 21.9] | 0.37 | 26.7 [24.6, 28.6] | 43.7 [41.4, 46.1] | 2.83 |
|  | **DBM % (95% CIs)** |  | 13.3 [11.8, 14.9] | 12.7 [11.4, 14.1] | 15.7 [14.6, 16.9] | 0.13 | 4.5 [3.8, 5.4] | 5.3 [4.7, 6.0] | 0.11 | 1.3 [0.9, 1.8] | 2.2 [1.8, 2.6] | 0.15 | 13.7 [12.6, 15.0] | 13.2 [12.3, 14.4] | 16.0 [14.5, 17.5] | 0.16 | 14.9 [12.9, 17.1] | 15.9 [13.8, 18.1] | 0.17 | 6.1 [5.3, 7.0] | 5.7 [4.9, 6.6] | -0.07 | 1.0 [0.7, 1.4] | 0.7 [0.5, 1.0] | 1.7 [1.3, 2.1] | 0.06 | 13.7 [12.2, 15.3] | 16.5 [14.9, 18.2] | 0.47 |
|  | **N** |  | 2,070 | 3,514 | 5,274 |  | 2,707 | 5,671 |  | 2,794 | 5,675 |  | 3,253 | 5,148 | 4,430 |  | 2,167 | 3,779 |  | 3,021 | 5,930 |  | 3,921 | 10,317 | 9,878 |  | 2,923 | 3,914 |  |
|  | **Survey year** |  | 2001 | 2011 | 2017 |  | 2003 | 2010 |  | 2010 | 2016 |  | 2004 | 2011 | 2018 |  | 2005 | 2011 |  | 2007 | 2013 |  | 2005 | 2011 | 2016 |  | 2013 | 2019 |  |
|  | **Income^2^** |  | LMI |  |  |  | LI |  |  | LI |  |  | LMI |  |  |  | LMI |  |  | LI |  |  | Li |  |  |  | LI |  |  |
|  | **Country** |  | Benin |  |  | AARC (%-points): | Burkina Faso |  | AARC (%-points): | Burundi |  | AARC (%-points): | Cameroon |  |  | AARC (%-points): | Congo |  | AARC (%-points): | DRC |  | AARC (%-points): | Ethiopia |  |  | AARC (%-points): | Gambia |  | AARC (%-points): |

| **Supplemental Table 4.** (continued)^1^ | **Anemia % (95% CIs)** | **AFRICAN REGION** | 43.0 [41.0, 45.0] | 56.9 [54.6, 59.3] | 40.7 [38.5, 43.0] | -0.29 | 51.0 [48.8, 53.1] | 47.4 [44.8, 50.0] | 44.1 [42.1, 46.2] | -0.53 | 32.7 [30.1, 35.3] | 26.6 [24.6, 28.8] | 27.2 [25.2, 29.3] | -0.55 | 42.8 [39.4, 46.3] | 35.3 [33.6, 37.1] | -0.47 | 43.2 [40.6, 45.9] | 27.6 [25.8, 29.4] | 30.5 [28.9, 32.1] | -1.20 | 61.5 [59.1, 63.8] | 59.5 [57.1, 61.8] | 49.6 [47.6, 51.6] | 61.8 [59.4, 64.2] | -0.15 | 41.4 [39.0, 43.9] | 42.2 [39.7, 44.7] | 0.13 |
| --- | --- | --- | --- | --- | --- | --- | --- | --- | --- | --- | --- | --- | --- | --- | --- | --- | --- | --- | --- | --- | --- | --- | --- | --- | --- | --- | --- | --- | --- |
|  | **OWOB % (95% CIs)** |  | 29.4 [27.5, 31.5] | 35.2 [33.2, 37.2] | 47.1 [44.7, 49.6] | 1.62 | 16.4 [14.8, 18.1] | 22.8 [20.8, 25.0] | 31.5 [29.4, 33.8] | 1.15 | 46.8 [44.2, 49.4] | 48.8 [46.3, 51.2] | 51.9 [49.5, 54.3] | 0.51 | 7.6 [6.4, 9.1] | 7.7 [6.8, 8.8] | 1.02 | 15.8 [13.9, 17.9] | 20.1 [18.6, 21.6] | 24.6 [23.2, 26.0] | 0.80 | 16.9 [14.8, 19.2] | 22.3 [19.0, 25.9] | 20.6 [18.8, 22.5] | 31.5 [29.0, 34.0] | 0.74 | 15.7 [14.2, 17.4] | 20.7 [18.9, 22.7] | 0.83 |
|  | **DBM % (95% CIs)** |  | 10.3 [9.2, 11.6] | 18.5 [17.0, 20.1] | 17.0 [15.2, 19.0] | 0.58 | 7.6 [6.5, 9.0] | 8.8 [7.8, 10.1] | 12.7 [11.4, 14.1] | 0.38 | 13.6 [11.9, 15.4] | 11.1 [9.6, 12.8] | 12.4 [10.9, 14.1] | -0.12 | 2.1 [1.6, 2.8] | 2.2 [1.8, 2.7] | 0.02 | 5.2 [4.1, 6.6] | 4.5 [3.9, 5.3] | 6.8 [6.0, 7.6] | 0.14 | 8.8 [7.2, 10.6] | 11.1 [8.6, 14.4] | 8.3 [7.3, 9.3] | 15.8 [14.3, 17.4] | 0.32 | 4.7 [3.9, 5.8] | 7.7 [6.6, 8.9] | 0.50 |
|  | **N** |  | 3,620 | 3,276 | 3,363 |  | 2,577 | 3,065 | 3,439 |  | 2,048 | 2,732 | 2,383 |  | 1,802 | 5,585 |  | 1,779 | 4,817 | 5,553 |  | 2,275 | 2,976 | 3,505 | 3,375 |  | 2,758 | 3,303 |  |
|  | **Survey year** |  | 2003 | 2008 | 2014 |  | 2005 | 2012 | 2018 |  | 2004 | 2009 | 2014 |  | 2003 | 2008 |  | 2004 | 2010 | 2015 |  | 2001 | 2006 | 2012 | 2018 |  | 2006 | 2012 |  |
|  | **Income^2^** |  | LMI |  |  |  | LI |  |  |  | LMI |  |  |  | LI |  |  | LI |  |  |  | LI |  |  |  |  | LI |  |  |
|  | **Country** |  | Ghana |  |  | AARC (%-points): | Guinea |  |  | AARC (%-points): | Lesotho |  |  | AARC (%-points): | Madagascar |  | AARC (%-points): | Malawi |  |  | AARC (%-points): | Mali |  |  |  | AARC (%-points): | Niger |  | AARC (%-points): |

| **Supplemental Table 4.** (continued)^1^ | **Anemia % (95% CIs)** | **AFRICAN REGION** | 25.7 [24.0, 27.5] | 17.3 [16.2, 18.5] | 18.6 [17.2, 20.0] | 11.5 [10.5, 12.6] | -0.91 | 57.0 [54.5, 59.6] | 53.4 [50.9, 55.9] | -0.72 | 43.8 [41.3, 46.3] | 42.0 [39.3, 44.7] | 44.5 [42.5, 46.6] | 0.08 | 45.3 [43.4, 47.2] | 37.2 [35.7, 39.0] | 42.3 [40.6, 43.9] | -0.31 | 35.9 [34.0, 37.9] | 41.1 [38.2, 44.1] | 23.2 [21.2, 25.2] | 29.5 [27.7, 31.4] | -0.68 | 37.2 [35.1, 39.4] | 28.2 [26.8, 29.7] | 26.3 [24.9, 27.7] | -1.09 |
| --- | --- | --- | --- | --- | --- | --- | --- | --- | --- | --- | --- | --- | --- | --- | --- | --- | --- | --- | --- | --- | --- | --- | --- | --- | --- | --- | --- |
|  | **OWOB % (95% CIs)** |  | 12.1 [10.9, 13.4] | 17.9 [16.8, 19.1] | 22.9 [21.6, 24.3] | 29.7 [28.1, 31.4] | 1.26 | 27.8 [25.2, 30.7] | 25.8 [23.3, 28.4] | -0.40 | 31.4 [28.3, 34.8] | 21.6 [19.8, 23.5] | 32.6 [30.8, 34.4] | 0.17 | 20.8 [19.4, 22.4] | 24.8 [23.1, 26.5] | 33.5 [31.9, 35.2] | 1.14 | 14.7 [13.3, 16.3] | 18.8 [16.5, 21.5] | 21.3 [19.3, 23.3] | 27.9 [25.9, 29.9] | 0.79 | 28.9 [26.9, 30.8] | 36.4 [34.9, 37.9] | 41.2 [39.5, 42.9] | 1.23 |
|  | **DBM % (95% CIs)** |  | 2.9 [2.3, 3.5] | 2.7 [2.2, 3.3] | 3.4 [3.0, 4.0] | 2.8 [2.3, 3.4] | 0.01 | 15.1 [13.3, 17.0] | 12.8 [11.1, 14.7] | -0.46 | 14.1 [12.2, 16.2] | 8.4 [7.4, 9.6] | 12.9 [11.9, 14.1] | -0.08 | 8.4 [7.5, 9.5] | 8.6 [7.7, 9.6] | 12.0 [11.1, 13.0] | 0.32 | 3.4 [2.7, 4.1] | 6.1 [4.9, 7.6] | 3.5 [2.7, 4.5] | 6.4 [5.5, 7.5] | 0.12 | 8.9 [8.1, 9.8] | 8.8 [7.9, 9.7] | 9.9 [9.0, 10.9] | 0.10 |
|  | **N** |  | 3,667 | 4,790 | 4,685 | 5,057 |  | 2,752 | 3,614 |  | 2,418 | 5,271 | 5,052 |  | 6,612 | 6,593 | 8,791 |  | 4,096 | 1,765 | 1,693 | 3,868 |  | 5,309 | 5,636 | 6,515 |  |
|  | **Survey year** |  | 2005 | 2010 | 2014 | 2019 |  | 2005 | 2010 |  | 2008 | 2013 | 2019 |  | 2004 | 2010 | 2015 |  | 2000 | 2006 | 2011 | 2016 |  | 2005 | 2010 | 2015 |  |
|  | **Income^2^** |  | LI |  |  |  |  | LMI |  |  | LI |  |  |  | LMI |  |  |  | LI |  |  |  |  | LMI |  |  |  |
|  | **Country** |  | Rwanda |  |  |  | AARC (%-points): | Senegal |  | AARC (%-points): | Sierra Leone |  |  | AARC (%-points): | Tanzania |  |  | AARC (%-points): | Uganda |  |  |  | AARC (%-points): | Zimbabwe |  |  | AARC (%-points): |

| **Supplemental Table 4.** (continued)^1^ | **Anemia % (95% CIs)** | **EASTERN MEDITERRANEAN REGION** | 27.4 [25.9, 28.9] | 39.1 [39.1, 39.1] | 25.6 [24.1, 27.2] | -0.29 | 27.4 [24.3, 30.6] | 36.4 [36.2, 36.5] | 36.9 [34.8, 38.9] | 44.7 [42.4, 47.0] | 1.05 | **EUROPEAN REGION** | 19.2 [17.8, 20.8] | 21.7 [20.5, 22.9] | 0.28 | 12.3 [11.2, 13.5] | 23.1 [21.2, 25.0] | 12.9 [11.5, 14.5] | -0.11 | **AMERICAS REGION** | 31.8 [30.0, 33.8] | 36.5 [34.6, 38.5] | 0.94 | 53.1 [49.8, 56.3] | 43.4 [41.1, 45.7] | 46.9 [45.1, 48.7] | 47.0 [45.3, 48.7] | -0.26 |
| --- | --- | --- | --- | --- | --- | --- | --- | --- | --- | --- | --- | --- | --- | --- | --- | --- | --- | --- | --- | --- | --- | --- | --- | --- | --- | --- | --- | --- |
|  | **OWOB % (95% CIs)** |  | 78.8 [77.5, 80.1] | 81.7 [81.7, 81.8] | 85.4 [84.3, 86.5] | 0.46 | 69.3 [66.1, 72.3] | 67.8 [67.7, 68.0] | 72.0 [70.0, 73.9] | 69.9 [67.9, 71.8] | 0.12 |  | 46.8 [45.1, 48.5] | 60.8 [59.4, 62.2] | 1.56 | 47.2 [45.7, 48.7] | 49.1 [47.0, 51.1] | 49.9 [48.2, 51.6] | 0.17 |  | 53.7 [51.7, 55.8] | 56.7 [54.7, 58.7] | 0.60 | 28.2 [24.7, 32.1] | 26.0 [24.2, 28.0] | 31.1 [29.3, 33.0] | 38.5 [37.0, 40.2] | 0.64 |
|  | **DBM % (95% CIs)** |  | 20.4 [19.0, 21.8] | 31.3 [31.3, 31.4] | 21.4 [20.0, 22.8] | -0.07 | 19.3 [16.8, 22.0] | 24.2 [24.1, 24.4] | 25.8 [23.9, 27.7] | 30.9 [29.1, 32.8] | 0.73 |  | 9.1 [8.1, 10.1] | 12.6 [11.8, 13.5] | 0.39 | 5.2 [4.5, 5.9] | 10.6 [9.5, 11.8] | 5.7 [4.8, 6.7] | -0.04 |  | 15.2 [13.7, 16.9] | 18.5 [17.0, 20.1] | 0.66 | 12.8 [10.3, 15.8] | 9.6 [8.3, 11.1] | 13.1 [11.9, 14.4] | 16.6 [15.3, 17.9] | 0.27 |
|  | **N** |  | 6,422 | 5,322 | 6,077 |  | 1,468 | 4,147 | 5,902 | 5,879 |  |  | 5,796 | 12,360 |  | 4,795 | 4,838 | 4,868 |  |  | 4,147 | 4,063 |  | 3,136 | 3,483 | 6,400 | 6,587 |  |
|  | **Survey year** |  | 2000 | 2005 | 2014 |  | 2002 | 2007 | 2012 | 2017 |  |  | 2008 | 2017 |  | 2000 | 2005 | 2015 |  |  | 2003 | 2008 |  | 2000 | 2005 | 2012 | 2016 |  |
|  | **Income^2^** |  | LMI |  |  |  | UMI |  |  |  |  |  | UMI |  |  | UMI |  |  |  |  | LMI |  |  | LMI |  |  |  |  |
|  | **Country** |  | Egypt |  |  | AARC (%-points): | Jordan |  |  |  | AARC (%-points): |  | Albania |  | AARC (%-points): | Armenia |  |  | AARC (%-points): |  | Bolivia |  | AARC (%-points): | Haiti |  |  |  | AARC (%-points): |

| **Supplemental Table 4.** (continued)^1^ | **Anemia % (95% CIs)** | **AMERICAS REGION** | 18.3 [17.2, 19.4] | 15.5 [14.7, 16.2] | -0.47 | 30.8 [29.0, 32.5] | 25.8 [24.7, 26.8] | 20.3 [19.3, 21.3] | 20.9 [20.0, 21.8] | 16.5 [15.7, 17.3] | 16.8 [16.0, 17.7] | -1.22 | **SOUTHEAST ASIAN REGION** | 51.3 [50.9, 51.6] | 52.9 [52.6, 53.1] | 56.7 [56.4, 56.9] | 0.34 | 34.1 [30.5, 37.9] | 33.0 [30.6, 35.4] | 39.5 [37.4, 41.8] | 0.54 | 19.7 [17.9, 21.6] | 21.7 [19.8, 23.7] | 0.29 | **WESTERN PACIFIC REGION** | 58.5 [56.3, 60.7] | 45.8 [44.2, 47.4] | 42.9 [41.4, 44.5] | 43.8 [42.4, 45.1] | -1.03 | ^1^Values are prevalence estimates and 95% CIs, unless otherwise indicated. A positive AARC value depicts an increase in %-points in malnutrition over time; whereas a negative value means that the prevalence is decreasing. Abbreviations: AARC,  average annual rate of change; DBM, co-occurrent overweight/obesity and anemia; DRC, Democratic Republic of the Congo; OWOB, overweight/obesity (no anemia).  ^2^Latest World Bank Income classification: LI, low-income; LMI, lower-middle-income; UMI, upper-middle-income. |
| --- | --- | --- | --- | --- | --- | --- | --- | --- | --- | --- | --- | --- | --- | --- | --- | --- | --- | --- | --- | --- | --- | --- | --- | --- | --- | --- | --- | --- | --- | --- | --- |
|  | **OWOB % (95% CIs)** |  | 53.9 [52.9, 55.0] | 60.0 [58.9, 61.0] | 1.02 | 54.3 [52.4, 56.1] | 54.8 [53.6, 56.0] | 57.8 [56.7, 59.0] | 57.6 [56.5, 58.7] | 59.3 [58.2, 60.5] | 61.8 [60.7, 62.8] | 0.54 |  | 18.5 [18.2, 18.7] | 24.3 [24.0, 24.5] | 27.8 [27.6, 28.0] | 0.65 | 10.4 [8.9, 12.2] | 16.3 [14.5, 18.4] | 26.9 [24.7, 29.1] | 1.65 | 7.5 [6.2, 9.0] | 13.5 [12.1, 15.1] | 0.86 |  | 8.6 [7.4, 10.0] | 11.9 [10.7, 13.1] | 13.2 [12.0, 14.6] | 21.0 [19.9, 12.4] | 0.80 |  |
|  | **DBM % (95% CIs)** |  | 9.0 [8.3, 9.7] | 9.1 [8.5, 9.7] | 0.02 | 15.5 [14.3, 16.9] | 13.1 [12.3, 13.8] | 10.8 [10.2, 11.6] | 10.4 [9.8, 11.1] | 8.8 [8.2, 9.4] | 9.4 [8.8, 10.0] | -0.56 |  | 7.7 [7.5, 7.9] | 11.4 [11.2, 11.6] | 14.4 [14.3, 14.6] | 0.46 | 2.2 [1.8, 2.8] | 3.5 [2.8, 4.2] | 8.0 [7.0, 9.2] | 0.58 | 1.5 [0.9, 2.3] | 2.5 [1.9, 3.3] | 0.14 |  | 3.6 [2.8, 4.5] | 3.6 [3.1, 4.2] | 4.5 [3.8, 5.2] | 7.7 [6.9, 8.4] | 0.27 |  |
|  | **N** |  | 12,941 | 15,323 |  | 4,559 | 18,728 | 16,798 | 16,649 | 16,716 | 17,898 |  |  | 84,976 | 524,796 | 538,221 |  | 7,598 | 4,439 | 4,787 |  | 2,696 | 2,870 |  |  | 2,500 | 5,875 | 6,744 | 8,703 |  |  |
|  | **Survey year** |  | 2005 | 2011 |  | 2000 | 2007 | 2009 | 2010 | 2011 | 2012 |  |  | 2005 | 2015 | 2019 |  | 2006 | 2011 | 2016 |  | 2009 | 2016 |  |  | 2000 | 2005 | 2010 | 2014 |  |  |
|  | **Income^2^** |  | LMI |  |  | UMI |  |  |  |  |  |  |  | LMI |  |  |  | LMI |  |  |  | LMI |  |  |  | LMI |  |  |  |  |  |
|  | **Country** |  | Honduras |  | AARC (%-points): | Peru |  |  |  |  |  | AARC (%-points): |  | India |  |  | AARC (%-points): | Nepal |  |  | AARC (%-points): | Timor-Leste |  | AARC (%-points): |  | Cambodia |  |  |  | AARC (%-points): |  |

| **Supplemental Table 5.** Trends in the prevalence of co-occurrent overweight/obesity and anemia among adult women (20-49 years old) by household wealth^1^ | **Household wealth quintiles** | **p-value** | **AFRICAN REGION** | 0.0000 | 0.0000 | 0.0000 |  | 0.0000 | 0.0000 |  | 0.0030 | 0.0000 |  | 0.0000 | 0.0000 | 0.0000 |  | 0.0000 | 0.0000 |  | 0.0000 | 0.0000 |  | 0.0350 | 0.0000 | 0.0000 |  | 0.0000 | 0.0950 |  |
| --- | --- | --- | --- | --- | --- | --- | --- | --- | --- | --- | --- | --- | --- | --- | --- | --- | --- | --- | --- | --- | --- | --- | --- | --- | --- | --- | --- | --- | --- | --- |
|  |  | **SII^2^** |  | 31.1 | 16.9 | 24.7 | -1.13 | 15.5 | 12.3 | -0.21 | 3.4 | 4.1 | 0.42 | 23.6 | 23.0 | 22.1 | 0.22 | 16.7 | 25.4 | 1.42 | 15.1 | 16.6 | -0.35 | 1.7 | 3.9 | 3.5 | 0.26 | 9.1 | 3.4 | -1.07 |
|  |  | **Richest (Q5)** |  | 29.2 [25.7, 32.9] | 18.8 [15.8, 22.3] | 26.6 [23.9, 29.5] | -0.25 | 12.5 [10.0, 15.3] | 12.1 [10.5, 13.7] | -0.06 | 2.6 [1.7, 4.0] | 4.3 [3.0, 6.2] | 0.28 | 23.3 [20.8, 26.0] | 21.2 [18.9, 23.6] | 26.5 [23.1, 30.3] | 0.23 | 21.6 [17.5, 26.3] | 25.3 [20.0, 31.4] | 0.61 | 14.2 [11.7, 17.0] | 13.8 [11.6, 16.5] | -0.07 | 1.7 [1.2, 2.5] | 1.9 [1.3, 2.9] | 3.7 [2.8, 4.9] | 0.18 | 14.6 [11.4, 18.6] | 13.3 [10.7, 16.5] | -0.22 |
|  |  | **Richer (Q4)** |  | 12.4 [10.0, 15.3] | 14.7 [12.2, 17.6] | 16.8 [14.3, 19.6] | 0.27 | 3.5 [2.1, 5.6] | 4.3 [2.9, 6.2] | 0.11 | 0.8 [0.3, 2.2] | 2.4 [1.6, 3.7] | 0.27 | 17.6 [14.5, 21.3] | 16.2 [13.8, 18.8] | 20.5 [17.8, 23.4] | 0.21 | 18.3 [15.2, 22.0] | 21.0 [16.0, 27.2] | 0.45 | 5.8 [4.1, 8.0] | 5.6 [3.6, 8.6] | -0.03 | 0.4 [0.3, 0.4] | 0.5 [0.3, 1.1] | 1.1 [0.7, 2.0] | 0.06 | 17.2 [13.7, 21.4] | 20.9 [17.1, 25.4] | 0.62 |
|  |  | **Middle (Q3)** |  | 8.9 [6.7, 11.7] | 13.0 [10.6, 15.8] | 14.0 [11.8, 16.5] | 0.33 | 2.4 [1.3,3.9] | 3.1 [2.2, 4.5] | 0.10 | 1.7 [0.9, 3.2] | 1.5 [0.9, 2.6] | -0.03 | 13.8 [11.4, 16.5] | 12.8 [10.6, 15.4] | 14.7 [12.3, 17.4] | 0.06 | 13.7 [11.8, 16.0] | 15.5 [11.6, 20.4] | 0.30 | 3.8 [2.4, 5.7] | 3.8 [2.7, 5.5] | 0.00 | 0.7 [0.3, 1.9] | 0.5 [0.0, 0.2] | 0.5 [0.2, 1.0] | -0.02 | 15.3 [11.7, 19.7] | 18.4 [14.8, 22.6] | 0.52 |
|  |  | **Poorer (Q2)** |  | 6.1 [4.2, 8.7] | 8.5 [6.5, 11.0] | 10.0 [8.1, 12.3] | 0.24 | 1.3 [5.1, 2.6] | 2.2 [1.4, 3.5] | 0.13 | 0.2 [0.0, 1.5] | 1.5 [0.9, 2.4] | 0.22 | 7.2 [5.6, 9.1] | 7.9 [6.3, 9.9] | 9.1 [7.2, 11.4] | 0.14 | 8.3 [5.9, 11.7] | 10.0 [7.5, 13.0] | 0.28 | 1.9 [1.0, 3.5] | 1.8 [1.1, 3.0] | -0.02 | 1.0 [0.6, 1.5] | 0.1 [0.0, 0.6] | 0.7 [0.3, 1.5] | -0.03 | 8.7 [6.5, 11.5] | 16.2 [13.1, 19.8] | 1.25 |
|  |  | **Poorest (Q1)** |  | 5.1 [3.3, 8.0] | 5.5 [4.1, 7.3] | 6.4 [4.8, 8.5] | 0.08 | 1.4 [0.5, 3.0] | 2.8 [1.8, 4.2] | 0.20 | 1.2 [0.5, 3.1] | 1.0 [0.5, 2.0] | -0.03 | 3.9 [2.9, 5.2] | 2.7 [1.6, 4.4] | 3.3 [2.0, 5.4] | -0.04 | 10.0 [6.7, 14.6] | 6.2 [5.0, 7.8] | -0.63 | 3.2 [2.0, 5.0] | 1.9 [1.3, 2.9] | -0.22 | 1.1 [0.4, 2.7] | 1.1 [0.4, 2.7] | 1.5 [0.9, 2.4] | 0.03 | 11.7 [8.9, 15.3] | 13.6 [11.5, 16.1] | 0.32 |
|  |  | **Survey year** |  | 2001 | 2011 | 2017 |  | 2003 | 2010 |  | 2010 | 2016 |  | 2004 | 2011 | 2018 |  | 2005 | 2011 |  | 2007 | 2013 |  | 2005 | 2011 | 2016 |  | 2013 | 2019 |  |
|  |  | **Country** |  | Benin |  |  | AARC (%-points): | Burkina Faso |  | AARC (%-points): | Burundi |  | AARC (%-points): | Cameroon |  |  | AARC (%-points): | Congo |  | AARC (%-points): | DRC |  | AARC (%-points): | Ethiopia |  |  | AARC (%-points): | Gambia |  | AARC (%-points): |

| **Supplemental Table 5.** (continued)^1^ | **Household wealth quintiles** | **p-value** | **AFRICAN REGION** | 0.0000 | 0.0000 | 0.0000 |  | 0.0000 | 0.0000 | 0.0000 |  | 0.0000 | 0.0000 | 0.0000 |  | 0.0250 | 0.0000 |  | 0.0060 | 0.0030 | 0.0000 |  | 0.0000 | 0.0000 | 0.0000 | 0.0000 |  | 0.0000 | 0.0000 |  |
| --- | --- | --- | --- | --- | --- | --- | --- | --- | --- | --- | --- | --- | --- | --- | --- | --- | --- | --- | --- | --- | --- | --- | --- | --- | --- | --- | --- | --- | --- | --- |
|  |  | **SII^2^** |  | 18.4 | 29.8 | 21.1 | 0.18 | 14.1 | 14.6 | 17.0 | 0.06 | 16.6 | 10.5 | 12.2 | -0.14 | 3.9 | 5.1 | 0.66 | 5.5 | 3.2 | 10.7 | 0.31 | 15.2 | 15.1 | 14.5 | 11.5 | -0.51 | 17.9 | 13.1 | -0.15 |
|  |  | **Richest (Q5)** |  | 17.7 [14.9, 20.9] | 27.8 [23.8, 32.1] | 25.2 [20.8, 30.2] | 0.65 | 17.8 [14.4, 21.8] | 14.6 [12.3, 17.2] | 18.8 [15.8, 22.2] | 0.06 | 17.9 [14.5, 21.8] | 13.7 [11.2, 16.6] | 16.0 [12.7, 19.9] | -0.19 | 2.6 [1.9, 3.6] | 4.6 [3.4, 6.3] | 0.40 | 9.0 [6.2, 12.9] | 6.6 [5.1, 8.7] | 11.6 [9.5, 13.9] | 0.22 | 17.0 [12.8, 22.2] | 17.7 [14.7, 21.2] | 14.2 [12.1, 16.7] | 19.8 [16.4, 23.7] | 0.09 | 11.3 [9.4, 13.6] | 16.0 [13.4, 19.1] | 0.78 |
|  |  | **Richer (Q4)** |  | 12.6 [10.3, 15.3] | 25.7 [22.2, 29.5] | 18.2 [14.5, 22.5] | 0.45 | 7.6 [5.4, 10.6] | 11.6 [9.0, 14.7] | 17.3 [14.1, 21.0] | 0.74 | 16.4 [13.2, 20.1] | 13.1 [9.7, 17.3] | 14.3 [11.0, 18.5] | -0.21 | 2.9 [2.0, 4.0] | 2.4 [1.6, 3.6] | -0.10 | 6.4 [4.5, 8.9] | 2.7 [1.7, 4.2] | 7.9 [6.1, 10.0] | 0.11 | 9.0 [7.0, 11.5] | 15.0 [8.5, 25.1] | 9.9 [7.4, 12.9] | 19.1 [15.9, 22.7] | 0.44 | 5.5 [3.8, 7.8] | 6.1 [4.4, 8.5] | 0.10 |
|  |  | **Middle (Q3)** |  | 7.3 [5.6, 9.4] | 14.5 [12.0, 17.4] | 16.7 [13.6, 20.4] | 0.84 | 5.4 [4.0, 7.2] | 8.2 [5.9, 11.3] | 10.7 [8.2, 13.8] | 0.41 | 12.8 [10.2, 15.8] | 10.1 [7.2, 14.0] | 12.8 [9.6, 16.9] | -0.00 | 1.7 [0.7, 4.0] | 1.2 [0.6, 2.7] | -0.10 | 3.9 [2.4, 6.3] | 5.4 [3.7, 7.8] | 5.7 [4.3, 7.4] | 0.17 | 7.8 [5.8, 10.5] | 7.7 [5.8, 10.3] | 6.6 [4.7, 9.2] | 16.1 [11.4, 17.3] | 0.43 | 2.6 [1.5, 4.3] | 5.6 [3.9, 8.2] | 0.50 |
|  |  | **Poorer (Q2)** |  | 6.7 [5.0, 8.9] | 11.2 [8.7, 14.3] | 14.3 [11.4, 17.7] | 0.69 | 3.0 [1.8, 5.0] | 5.2 [3.5, 7.5] | 11.2 [8.7, 14.5] | 0.62 | 10.1 [7.5, 13.6] | 8.9 [6.5, 12.0] | 7.9 [5.4, 11.2] | -0.22 | 1.6 [1.3, 1.9] | 1.0 [0.5, 2.0] | -0.12 | 3.1 [1.8, 5.2] | 4.2 [2.9, 5.9] | 3.3 [2.3, 4.8] | 0.02 | 4.8 [3.2, 7.1] | 8.0 [6.2, 10.3] | 4.3 [2.8, 6.7] | 13.7 [11.1, 16.9] | 0.41 | 2.1 [1.1, 4.1] | 5.4 [3.6, 8.0] | 0.55 |
|  |  | **Poorest (Q1)** |  | 3.7 [2.6, 5.2] | 6.9 [4.9, 9.8] | 5.5 [4.0, 7.6] | 0.15 | 4.5 [2.8, 7.0] | 3.3 [2.0, 5.4] | 5.2 [3.8, 7.1] | 0.05 | 7.3 [6.0, 8.9] | 6.1 [4.2, 8.8] | 7.0 [4.6, 10.7] | -0.03 | 1.5 [0.6, 4.0] | 1.0 [0.5, 2.0] | -0.10 | 3.9 [2.5, 6.2] | 3.0 [1.8, 4.9] | 4.2 [2.9, 5.9] | 0.02 | 3.5 [2.0, 6.2] | 5.0 [3.5, 7.1] | 5.6 [4.0, 7.6] | 11.2 [8.6, 14.5] | 0.42 | 1.2 [0.6, 2.3] | 4.3 [2.4, 7.6] | 0.52 |
|  |  | **Survey year** |  | 2003 | 2008 | 2014 |  | 2005 | 2012 | 2018 |  | 2004 | 2009 | 2014 |  | 2003 | 2008 |  | 2004 | 2010 | 2015 |  | 2001 | 2006 | 2012 | 2018 |  | 2006 | 2012 |  |
|  |  | **Country** |  | Ghana |  |  | AARC (%-points): | Guinea |  |  | AARC (%-points): | Lesotho |  |  | AARC (%-points): | Madagascar |  | AARC (%-points): | Malawi |  |  | AARC (%-points): | Mali |  |  |  | AARC (%-points): | Niger |  | AARC (%-points): |

| **Supplemental Table 5.** (continued)^1^ | **Household wealth quintiles** | **p-value** | **AFRICAN REGION** | 0.0040 | 0.0020 | 0.0180 | 0.0000 |  | 0.0000 | 0.0000 |  | 0.0850 | 0.0000 | 0.0000 |  | 0.0000 | 0.0000 | 0.0000 |  | 0.0000 | 0.0000 | 0.0000 | 0.0000 |  | 0.0000 | 0.0000 | 0.0000 |  |
| --- | --- | --- | --- | --- | --- | --- | --- | --- | --- | --- | --- | --- | --- | --- | --- | --- | --- | --- | --- | --- | --- | --- | --- | --- | --- | --- | --- | --- |
|  |  | **SII^2^** |  | 3.4 | 2.9 | 2.5 | 6.7 | 0.20 | 15.8 | 16.4 | 0.20 | 4.6 | 7.7 | 11.3 | 0.23 | 19.9 | 22.7 | 21.7 | 0.37 | 9.5 | 12.9 | 8.6 | 11.2 | 0.21 | 15.3 | 10.0 | 12.6 | -0.38 |
|  |  | **Richest (Q5)** |  | 5.5 [4.2, 7.3] | 4.2 [3.3, 5.5] | 5.4 [4.2, 6.8] | 5.4 [3.9, 7.5] | -0.55 | 18.7 [14.8, 23.5] | 19.1 [14.9, 24.2] | 0.08 | 19.8 [15.6, 24.8] | 11.6 [8.9, 15.0] | 16.9 [14.4, 19.6] | -0.23 | 18.0 [15.8, 20.6] | 18.3 [15.7, 21.1] | 21.4 [19.5, 23.5] | 0.30 | 6.2 [4.6, 8.3] | 11.0 [7.7, 15.4] | 5.9 [4.0, 8.3] | 11.5 [9.3, 14.1] | 0.21 | 15.1 [13.4, 16.9] | 13.8 [11.5, 16.4] | 14.3 [12.5, 16.2] | -0.08 |
|  |  | **Richer (Q4)** |  | 2.0 [1.3, 3.3] | 2.6 [1.6, 4.3] | 2.8 [1.9, 4.2] | 3.5 [2.4, 5.0] | -0.55 | 18.2 [14.3, 22.9] | 14.8 [11.5, 18.8] | -0.68 | 14.5 [11.6, 18.1] | 9.8 [7.9, 12.1] | 15.1 [12.8, 17.8] | 0.08 | 6.4 [5.2, 7.8] | 9.9 [8.2, 11.9] | 12.8 [10.9, 14.9] | 0.58 | 4.4 [2.7, 6.9] | 8.1 [5.5, 11.7] | 4.8 [2.7, 7.9] | 5.8 [4.1, 8.3] | 0.03 | 12.1 [10.1, 14.5] | 8.7 [7.1, 10.5] | 11.5 [9.6, 13.6] | -0.06 |
|  |  | **Middle (Q3)** |  | 2.5 [1.6, 3.9] | 2.3 [1.5, 3.4] | 2.3 [1.5, 3.6] | 2.1 [1.3, 3.4] | -0.65 | 18.2 [14.7, 22.3] | 11.5 [9.0, 14.5] | -1.34 | 11.5 [8.0, 16.1] | 8.0 [6.3, 10.2] | 12.6 [10.3, 15.4] | 0.12 | 5.4 [4.3, 7.0] | 4.3 [3.0, 6.3] | 7.9 [6.5, 9.6] | 0.21 | 1.6 [0.7, 3.6] | 5.1 [3.0, 8.7] | 4.0 [2.0, 7.0] | 5.3 [3.9, 7.3] | 0.19 | 6.1 [4.7, 7.8] | 6.9 [5.5, 8.7] | 10.0 [8.0, 12.3] | 0.39 |
|  |  | **Poorer (Q2)** |  | 2.3 [1.4, 3.6] | 1.8 [1.2, 2.7] | 3.4 [2.3, 4.9] | 1.5 [0.8, 2.6] | -0.26 | 8.3 [6.2, 11.1] | 7.1 [5.4, 9.3] | -0.24 | 11.6 [8.5, 15.6] | 6.2 [4.8, 8.1] | 9.8 [7.8, 12.2] | -0.14 | 4.6 [3.5, 6.0] | 4.2 [3.0, 5.9] | 6.2 [5.0, 7.7] | 0.14 | 1.4 [0.7, 3.0] | 2.4 [1.2, 4.9] | 1.2 [0.3, 3.5] | 3.5 [2.3, 5.4] | 0.10 | 3.2 [2.4, 4.4] | 6.9 [5.4, 8.8] | 5.9 [4.5, 7.6] | 0.27 |
|  |  | **Poorest (Q1)** |  | 2.2 [1.4, 3.3] | 2.2 [1.6, 3.2] | 2.9 [2.0, 4.2] | 0.9 [0.5, 1.8] | -0.27 | 7.4 [5.3, 10.2] | 7.0 [4.9, 9.9] | -0.08 | 12.9 [9.2, 17.8] | 5.9 [4.4, 7.8] | 8.9 [7.0, 11.4] | -0.34 | 4.2 [2.8, 6.2] | 3.3 [2.3, 4.7] | 5.6 [4.1, 7.7] | 0.12 | 2.1 [1.2, 3.7] | 1.7 [0.5, 5.6] | 0.3 [0.0, 1.6] | 3.2 [1.9, 5.1] | 0.03 | 4.7 [3.6, 6.1] | 5.8 [4.5, 7.4] | 5.0 [3.6, 6.8] | 0.03 |
|  |  | **Survey year** |  | 2005 | 2010 | 2014 | 2019 |  | 2005 | 2010 |  | 2008 | 2013 | 2019 |  | 2004 | 2010 | 2015 |  | 2000 | 2006 | 2011 | 2016 |  | 2005 | 2010 | 2015 |  |
|  |  | **Country** |  | Rwanda |  |  |  | AARC (%-points): | Senegal |  | AARC (%-points): | Sierra Leone |  |  | AARC (%-points): | Tanzania |  |  | AARC (%-points): | Uganda |  |  |  | AARC (%-points): | Zimbabwe |  |  | AARC (%-points): |

| **Supplemental Table 5.** (continued)^1^ | **Household wealth quintiles** | **p-value** | **EASTERN MEDITERRANEAN REGION** | 0.0470 | 0.0000 | 0.4980 |  | 0.3220 | 0.0050 | 0.0400 | 0.1070 |  | **EUROPEAN REGION** | 0.0700 | 0.9280 |  | 0.1420 | 0.3950 | 0.8520 |  | **AMERICAS REGION** | 0.0030 | 0.1340 |  | 0.0000 | 0.0000 | 0.0000 | 0.0000 |  |
| --- | --- | --- | --- | --- | --- | --- | --- | --- | --- | --- | --- | --- | --- | --- | --- | --- | --- | --- | --- | --- | --- | --- | --- | --- | --- | --- | --- | --- | --- |
|  |  | **SII^2^** |  | 3.4 | 11.9 | -1.8 | -0.43 | -4.0 | 6.5 | 4.2 | -5.1 | -0.10 |  | -2.2 | -0.1 | 0.56 | -2.5 | 1.3 | -0.3 | 0.18 |  | -5.5 | -3.8 | 0.36 | 29.7 | 17.3 | 19.9 | 21.8 | -0.36 |
|  |  | **Richest (Q5)** |  | 19.8 [16.9, 23.0] | 36.5 [33.7, 39.4] | 23.0 [20.1, 26.3] | 0.02 | 16.9 [9.5, 28.2] | 25.8 [21.8, 30.1] | 24.4 [18.7, 31.2] | 31.2 [27.3, 35.4] | 0.83 |  | 6.7 [5.4, 8.4] | 11.5 [9.5, 13.9] | 0.53 | 5.4 [3.8, 7.6] | 11.2 [8.7, 14.4] | 4.7 [3.2, 7.1] | -0.13 |  | 10.2 [8.6, 12.1] | 14.5 [12.3, 16.9] | 0.86 | 26.2 [21.7, 31.2] | 16.9 [13.8, 20.4] | 20.8 [18.1, 23.8] | 24.4 [22.0, 27.0] | -0.03 |
|  |  | **Richer (Q4)** |  | 22.2 [19.8, 24.8] | 36.0 [33.3, 39.0] | 18.9 [16.4, 21.8] | -0.43 | 16.4 [11.1, 23.4] | 25.7 [24.4, 27.1] | 23.0 [19.2, 27.2] | 29.4 [25.1, 34.0] | 0.73 |  | 7.6 [6.2, 9.2] | 13.2 [11.3, 15.4] | 0.62 | 4.3 [2.9, 6.5] | 10.8 [8.0, 14.4] | 6.3 [4.6, 8.5] | 0.05 |  | 16.8 [14.5, 19.4] | 19.4 [16.2, 23.1] | 0.52 | 20.0 [17.0, 23.4] | 10.4 [8.5, 12.6] | 13.9 [11.8, 16.2] | 19.1 [16.7, 21.8] | 0.01 |
|  |  | **Middle (Q3)** |  | 22.4 [20.4, 24.7] | 29.6 [29.6, 29.6] | 20.2[17.7, 23.0] | -0.26 | 15.8 [11.2, 21.6] | 25.8 [25.7, 26.0] | 27.4 [23.7, 31.5] | 27.1 [23.4, 31.0] | 0.71 |  | 10.0 [8.2, 12.1] | 13.3 [11.8, 15.0] | 0.37 | 6.0 [4.1, 8.6] | 11.9 [10.2, 14.0] | 6.3 [4.6, 8.6] | -0.06 |  | 20.0 [16.9, 23.5] | 22.8 [19.4, 26.7] | 0.56 | 14.2 [8.3, 23.3] | 7.2 [5.6, 9.1] | 12.4 [10.5, 14.5] | 14.2 [12.1, 16.5] | 0.10 |
|  |  | **Poorer (Q2)** |  | 19.6 [17.6, 21.9] | 30.6 [30.5, 30.7] | 21.8 [18.8, 25.1] | 0.02 | 22.6 [17.3, 29.0] | 21.2 [20.8, 21.5] | 28.9 [25.0, 33.2] | 32.4 [28.4, 36.7] | 0.74 |  | 11.7 [9.8, 13.9] | 13.6 [12.2, 15.2] | 0.21 | 8.2 [6.5, 10.2] | 10.7 [8.9, 12.8] | 6.3 [4.6, 8.5] | -0.17 |  | 15.3 [13.4, 17.5] | 19.6 [16.1, 23.6] | 0.86 | 3.0 [1.3, 6.8] | 5.4 [3.6, 7.8] | 7.9 [6.0, 10.2] | 9.6 [7.8, 11.8] | 0.40 |
|  |  | **Poorest (Q1)** |  | 17.7 [15.7, 19.8] | 27.0 [26.6, 27.4] | 23.5 [20.6, 26.7] | 0.32 | 21.0 [16.3, 26.6] | 20.9 [20.6, 21.2] | 24.7 [21.0, 28.8] | 34.9 [31.4, 38.5] | 0.91 |  | 9.6 [7.9, 11.8] | 11.5 [10.2, 13.0] | 0.21 | 7.9 [5.8, 10.6] | 8.1 [6.7, 9.9] | 4.8 [3.6, 6.4] | -0.22 |  | 14.4 [12.1, 17.1] | 16.3 [13.0, 20.3] | 0.38 | 5.3 [2.9, 9.6] | 2.2 [1.2, 4.0] | 3.9 [2.9, 5.3] | 8.4 [6.6, 10.7] | 0.19 |
|  |  | **Survey year** |  | 2000 | 2005 | 2014 |  | 2002 | 2007 | 2012 | 2017 |  |  | 2008 | 2017 |  | 2000 | 2005 | 2015 |  |  | 2003 | 2008 |  | 2000 | 2005 | 2012 | 2016 |  |
|  |  | **Country** |  | Egypt |  |  | AARC (%-points): | Jordan |  |  |  | AARC (%-points): |  | Albania |  | AARC (%-points): | Armenia |  |  | AARC (%-points): |  | Bolivia |  | AARC (%-points): | Haiti |  |  |  | AARC (%-points): |

| **Supplemental Table 5.** (continued)^1^ | **Household wealth quintiles** | **p-value** | **AMERICAS REGION** | 0.0000 | 0.0000 |  | 0.1510 | 0.0000 | 0.0000 | 0.0000 | 0.0000 | 0.7360 |  | **SOUTHEAST ASIAN REGION** | 0.0000 | 0.0000 | 0.0000 |  | 0.0000 | 0.0000 | 0.0000 |  | 0.0020 | 0.0000 |  | **WESTERN PACIFIC REGION** | 0.0000 | 0.0000 | 0.0000 | 0.0010 |  | ^1^Values are percentages and 95% CIs; estimates account for survey design. A positive AARC value depicts an increase in %-points in malnutrition over time; whereas a negative value means that the prevalence is decreasing. Abbreviations:  AARC, average annual rate of change; DRC, Democratic Republic of the Congo.  ^2^The SII represents the absolute difference in the fitted value of co-occurrent overweight/obesity and anemia between the highest and lowest wealth values. A positive value depicts that the DBM burden is  concentrated among the richest groups; whereas a negative value represents the opposite. Statistical significance= p-value <0.05. |
| --- | --- | --- | --- | --- | --- | --- | --- | --- | --- | --- | --- | --- | --- | --- | --- | --- | --- | --- | --- | --- | --- | --- | --- | --- | --- | --- | --- | --- | --- | --- | --- | --- |
|  |  | **SII^2^** |  | 7.6 | 9.4 | 0.33 | 3.7 | 3.4 | 3.9 | 3.2 | 2.9 | 0.4 | -0.18 |  | 20.3 | 20.6 | 18.8 | 0.03 | 9.2 | 11.9 | 14.4 | 0.64 | 3.3 | 7.4 | 0.31 |  | 6.9 | 6.4 | 3.5 | 3.7 | -0.30 |  |
|  |  | **Richest (Q5)** |  | 10.4 [9.1, 12.0] | 12.0 [10.5, 13.5] | 0.27 | 17.8 [14.7, 21.5] | 13.2 [11.9, 14.7] | 12.0 [10.5, 13.8] | 10.3 [8.8, 12.0] | 7.6 [6.4, 9.0] | 9.6 [8.1, 11.2] | -0.78 |  | 14.4 [14.0, 14.8] | 18.9 [18.4, 19.3] | 21.3 [20.9, 21.8] | 0.48 | 5.8 [4.9, 6.8] | 8.2 [6.4, 10.4] | 15.2 [12.7, 18.0] | 0.94 | 3.7 [2.0, 6.7] | 4.9 [3.2, 7.4] | 0.17 |  | 7.0 [5.1, 9.6] | 5.3 [4.2, 6.7] | 5.4 [4.1, 7.1] | 8.6 [7.4, 10.1] | 0.09 |  |
|  |  | **Richer (Q4)** |  | 11.3 [9.9, 12.9] | 11.2 [10.0, 12.5] | -0.02 | 15.8 [13.3, 18.7] | 15.7 [13.7, 17.9] | 12.2 [10.7, 13.8] | 12.0 [10.5, 13.7] | 10.1 [8.7, 11.7] | 9.5 [8.4, 10.7] | -0.53 |  | 7.9 [7.6, 8.3] | 15.4 [15.0, 15.8] | 17.9 [17.5, 18.2] | 0.72 | 2.7 [1.8, 4.2] | 3.8 [2.6, 5.5] | 8.6 [6.5, 11.2] | 0.59 | 1.4 [0.6, 3.5] | 4.6 [2.6, 7.8] | 0.46 |  | 6.7 [4.7, 9.3] | 5.3 [4.1, 6.9] | 5.1 [3.4, 7.5] | 8.6 [7.1, 10.4] | 0.10 |  |
|  |  | **Middle (Q3)** |  | 9.5 [8.3, 10.8] | 9.0 [7.7, 10.4] | -0.08 | 18.7 [16.5, 21.0] | 13.4 [12.0, 14.9] | 11.8 [10.5, 13.3] | 11.2 [9.9, 12.7] | 10.2 [8.9, 11.6] | 9.2 [8.1, 10.4] | -0.78 |  | 3.9 [3.6, 4.2] | 10.5 [10.2, 10.8] | 14.4 [14.1, 14.7] | 0.73 | 0.9 [0.5, 1.7] | 1.9 [1.0, 3.5] | 6.0 [4.4, 8.1] | 0.51 | 1.1 [0.5, 2.7] | 1.0 [0.4, 2.5] | -0.01 |  | 1.6 [0.8, 3.3] | 2.6 [1.8, 3.8] | 4.0 [2.9, 5.4] | 7.6 [6.2, 9.4] | 0.40 |  |
|  |  | **Poorer (Q2)** |  | 7.2 [6.3, 8.2] | 6.5 [5.5, 7.6] | -0.12 | 16.5 [14.1, 19.3] | 10.4 [9.2, 11.7] | 9.8 [8.6, 11.1] | 8.9 [7.8, 10.2] | 9.0 [7.8, 10.4] | 9.5 [8.4, 10.8] | -0.64 |  | 2.3 [2.0, 2.6] | 6.4 [6.2, 6.7] | 10.5 [10.2, 10.8] | 0.55 | 0.3 [0.1, 0.8] | 1.3 [0.7, 2.5] | 5.3 [4.0, 6.9] | 0.50 | 0.1 [0.0, 0.9] | 0.9 [0.3, 2.7] | 0.11 |  | 3.2 [2.3, 4.5] | 2.3 [1.5, 3.6] | 4.7 [3.3, 6.5] | 7.9 [6.3, 9.9] | 0.34 |  |
|  |  | **Poorest (Q1)** |  | 4.5 [3.8, 5.3] | 4.7 [4.0, 5.6] | 0.03 | 13.0 [11.4, 14.8] | 10.1 [8.4, 12.2] | 7.0 [5.9, 8.2] | 8.8 [7.8, 10.0] | 6.5 [5.4, 7.7] | 9.1 [8.0, 10.4] | -0.45 |  | 1.1 [0.9, 1.4] | 3.3 [3.2, 3.5] | 6.8 [6.5, 7.0] | 0.37 | 0.5 [0.2, 1.4] | 0.5 [0.2, 1.3] | 3.2 [2.1, 4.9] | 0.27 | 0.3 [0.0, 2.0] | 0.2 [0.0, 1.1] | -0.01 |  | 1.6 [0.7, 3.7] | 2.0 [1.3, 3.1] | 2.7 [1.8, 4.1] | 5.2 [4.0, 6.7] | 0.24 |  |
|  |  | **Survey year** |  | 2005 | 2011 |  | 2000 | 2007 | 2009 | 2010 | 2011 | 2012 |  |  | 2005 | 2015 | 2019 |  | 2006 | 2011 | 2016 |  | 2009 | 2016 |  |  | 2000 | 2005 | 2010 | 2014 |  |  |
|  |  | **Country** |  | Honduras |  | AARC (%-points): | Peru |  |  |  |  |  | AARC (%-points): |  | India |  |  | AARC (%-points): | Nepal |  |  | AARC (%-points): | Timor-Leste |  | AARC (%-points): |  | Cambodia |  |  |  | AARC (%-points): |  |

| **Supplemental Table 6.** Trends in the prevalence of co-occurrent overweight/obesity and anemia among adult women (20-49 years old) by education level^1^ | **Education level** | **p-value** | **AFRICAN REGION** | 0.0000 | 0.0000 | 0.0000 |  | 0.0000 | 0.0000 |  | 0.0300 | 0.0000 |  | 0.0000 | 0.0000 | 0.0000 |  | 0.1340 | 0.0000 |  | 0.0000 | 0.0000 |  | 0.0030 | 0.0000 | 0.0000 |  | 0.7230 | 0.0000 |  |
| --- | --- | --- | --- | --- | --- | --- | --- | --- | --- | --- | --- | --- | --- | --- | --- | --- | --- | --- | --- | --- | --- | --- | --- | --- | --- | --- | --- | --- | --- | --- |
|  |  | **SII^2^** |  | 21.6 | 7.9 | 8.5 | -0.93 | 12.5 | 8.6 | -0.37 | 2.1 | 3.0 | 0.28 | 19.4 | 17.6 | 14.3 | -0.20 | 4.9 | 15.5 | 2.23 | 10.0 | 9.6 | -0.12 | 2.3 | 1.8 | 2.4 | 0.11 | 0.9 | -10.5 | -1.45 |
|  |  | **Secondary+ (E3)** |  | 22.8 [18.5, 27.7] | 13.7 [10.8, 17.3] | 16.0 [13.8, 18.5] | -0.47 | 10.8 [6.7, 16.2] | 11.3 [9.0, 14.0] | 0.07 | 2.3 [1.3, 4.3] | 4.0 [2.9, 5.6] | 0.28 | 19.0 [17.2, 21.0] | 18.7 [17.0, 20.6] | 19.9 [17.8, 22.3] | 0.06 | 15.4 [13.0, 18.2] | 17.7 [15.0, 20.8] | 0.38 | 9.3 [7.7, 11.1] | 8.5 [7.1, 10.2] | -0.13 | 2.9 [1.7, 5.0] | 1.5 [0.9, 2.6] | 3.4 [2.2, 5.2] | 0.04 | 13.7 [10.9, 17.1] | 14.2 [12.1, 16.7] | 0.08 |
|  |  | **Primary (E2)** |  | 20.6 [17.3, 24.4] | 17.6 [14.3, 21.4] | 22.2 [19.2, 25.5] | 0.06 | 11.5 [8.0, 15.9] | 7.8 [5.9, 10.2] | -0.53 | 1.7 [0.9, 3.0] | 2.2 [1.6, 3.0] | 0.08 | 15.2 [13.0, 17.6] | 11.4 [10.0, 12.9] | 16.6 [14.1, 19.5] | 0.10 | 14.7 [11.1, 19.1] | 12.3 [9.6, 15.6] | -0.40 | 4.7 [3.5, 6.1] | 4.1 [3.2, 5.2] | -0.10 | 1.0 [0.6, 1.7] | 0.7 [0.4, 1.3] | 2.1 [1.3, 3.3] | 0.10 | 13.3 [9.3, 18.6] | 16.6 [12.9, 21.1] | 0.55 |
|  |  | **None (E1)** |  | 9.3 [7.8, 11.2] | 11.2 [9.9, 12.7] | 13.8 [12.5, 15.3] | 0.27 | 3.1 [2.4, 3.9] | 4.0 [3.4, 4.7] | 0.13 | 0.8 [0.5, 1.4] | 1.3 [0.9, 18.4] | 0.08 | 3.3 [2.3, 4.7] | 5.8 [4.3, 7.8] | 6.4 [4.7, 8.7] | 0.22 | 11.7 [6.5, 19.9] | 10.4 [5.4, 19.0] | -0.22 | 3.2 [2.0, 4.8] | 2.6 [1.5, 4.4] | -0.10 | 0.7 [0.4, 1.3] | 0.6 [0.4, 0.9] | 1.0 [0.7, 1.4] | 0.03 | 13.7 [11.7, 16.0] | 19.0 [17.0, 21.3] | 0.88 |
|  |  | **Survey year** |  | 2001 | 2011 | 2017 |  | 2003 | 2010 |  | 2010 | 2016 |  | 2004 | 2011 | 2018 |  | 2005 | 2011 |  | 2007 | 2013 |  | 2005 | 2011 | 2016 |  | 2013 | 2019 |  |
|  |  | **Country** |  | Benin |  |  | AARC (%-points): | Burkina Faso |  | AARC (%-points): | Burundi |  | AARC (%-points): | Cameroon |  |  | AARC (%-points): | Congo |  | AARC (%-points): | DRC |  | AARC (%-points): | Ethiopia |  |  | AARC (%-points): | Gambia |  | AARC (%-points): |

| **Supplemental Table 6.** (continued)^1^ | **Education level** | **p-value** | **AFRICAN REGION** | 0.0000 | 0.0000 | 0.0000 |  | 0.0000 | 0.0010 | 0.0860 |  | 0.0720 | 0.0220 | 0.0190 |  | 0.6850 | 0.0000 |  | 0.5570 | 0.0320 | 0.0000 |  | 0.0000 | 0.0030 | 0.0030 | 0.9950 |  | 0.0000 | 0.0000 |  |
| --- | --- | --- | --- | --- | --- | --- | --- | --- | --- | --- | --- | --- | --- | --- | --- | --- | --- | --- | --- | --- | --- | --- | --- | --- | --- | --- | --- | --- | --- | --- |
|  |  | **SII^2^** |  | 11.4 | 18.2 | 11.3 | 0.33 | 11.1 | 7.4 | 4.3 | -0.61 | 5.5 | 5.5 | 11.5 | 0.37 | 0.7 | 4.0 | 0.60 | 1.2 | 2.4 | 5.4 | -0.08 | 10.9 | 7.7 | 6.5 | 0.0 | -0.41 | 9.5 | 10.3 | 0.22 |
|  |  | **Secondary+ (E3)** |  | 12.4 [10.8, 14.3] | 20.9 [18.7, 23.2] | 19.3 [16.9, 22.0] | 0.60 | 15.7 [11.0, 21.9] | 13.6 [10.9, 16.8] | 14.6 [11.7, 18.0] | -0.09 | 15.5 [12.7, 18.7] | 13.1 [10.8, 15.8] | 14.4 [12.1, 17.0] | -0.11 | 2.1 [1.2, 3.8] | 3.6 [2.8, 4.8] | 0.30 | 6.3 [4.2, 9.5] | 6.1 [4.4, 8.6] | 8.6 [6.9, 10.7] | 0.20 | 13.0 [9.9, 16.9] | 14.2 [10.7, 18.6] | 9.2 [6.8, 12.4] | 15.2 [11.7, 19.5] | 0.03 | 11.0 [7.6, 15.6] | 12.8 [9.2, 17.6] | 0.30 |
|  |  | **Primary (E2)** |  | 11.1 [9.0, 13.8] | 20.6 [17.5, 24.2] | 16.2 [12.9, 20.1] | 0.42 | 11.9 [8.2, 17.0] | 9.4 [6.6, 13.3] | 14.3 [10.3, 19.5] | 0.17 | 12.3 [10.4, 14.5] | 8.9 [7.3, 10.7] | 9.7 [7.8, 12.0] | -0.26 | 2.0 [1.2, 3.3] | 1.6 [1.1, 2.3] | -0.08 | 5.7 [4.3, 7.4] | 4.5 [3.7, 5.5] | 5.7 [4.8, 6.8] | -0.01 | 15.3 [12.3, 18.8] | 11.5 [9.2, 14.4] | 12.4 [9.0, 16.9] | 18.6 [14.6, 23.3] | 0.20 | 7.5 [5.5, 10.3] | 12.0 [8.5, 16.6] | 0.75 |
|  |  | **None (E1)** |  | 6.5 [5.1, 8.3] | 11.2 [9.1, 13.7] | 11.3 [9.0, 14.0] | 0.42 | 6.4 [5.2, 7.8] | 7.7 [6.4, 9.2] | 12.1 [10.6, 13.7] | 0.43 | 16.2 [15.1, 17.2] | 17.3 [9.6, 29.4] | 17.2 [4.1, 50.2] | 0.10 | 2.2 [1.0, 4.6] | 1.7 [1.0, 2.9] | -0.10 | 3.8 [2.5, 5.7] | 2.9 [1.6, 5.2] | 7.4 [5.3, 10.1] | 0.31 | 7.4 [5.9, 9.4] | 10.8 [7.8, 14.9] | 7.7 [6.6, 8.9] | 15.5 [13.8, 17.3] | 0.37 | 4.0 [3.1, 5.2] | 6.9 [5.7, 8.2] | 0.48 |
|  |  | **Survey year** |  | 2003 | 2008 | 2014 |  | 2005 | 2012 | 2018 |  | 2004 | 2009 | 2014 |  | 2003 | 2008 |  | 2004 | 2010 | 2015 |  | 2001 | 2006 | 2012 | 2018 |  | 2006 | 2012 |  |
|  |  | **Country** |  | Ghana |  |  | AARC (%-points): | Guinea |  |  | AARC (%-points): | Lesotho |  |  | AARC (%-points): | Madagascar |  | AARC (%-points): | Malawi |  |  | AARC (%-points): | Mali |  |  |  | AARC (%-points): | Niger |  | AARC (%-points): |

| **Supplemental Table 6.** (continued)^1^ | **Education level** | **p-value** | **AFRICAN REGION** | 0.2850 | 0.1130 | 0.0020 | 0.0000 |  | 0.0060 | 0.0090 |  | 0.0300 | 0.1630 | 0.5220 |  | 0.0000 | 0.0000 | 0.0000 |  | 0.0000 | 0.0040 | 0.0600 | 0.0050 |  | 0.0230 | 0.0530 | 0.0040 |  |
| --- | --- | --- | --- | --- | --- | --- | --- | --- | --- | --- | --- | --- | --- | --- | --- | --- | --- | --- | --- | --- | --- | --- | --- | --- | --- | --- | --- | --- |
|  |  | **SII^2^** |  | 1.4 | 1.8 | 3.7 | 3.9 | 0.23 | 7.3 | 5.5 | -0.42 | 6.5 | 2.3 | 1.2 | 0.52 | 10.3 | 13.6 | 11.8 | 0.45 | 5.1 | 5.8 | 3.3 | 4.4 | 0.14 | 3.6 | 3.2 | 5.3 | 0.22 |
|  |  | **Secondary+ (E3)** |  | 5.2 [3.6, 7.4] | 4.3 [3.1, 5.8] | 4.6 [3.4, 6.1] | 4.4 [3.1, 6.1] | -0.58 | 18.3 [12.5, 25.9] | 14.0 [10.4, 18.5] | -0.86 | 17.5 [13.5, 22.4] | 10.0 [7.4, 13.4] | 13.1 [11.2, 15.3] | -0.37 | 17.0 [14.3, 20.1] | 16.2 [12.8, 20.3] | 17.3 [15.4, 19.3] | 0.02 | 5.5 [3.9, 7.6] | 7.6 [5.0, 11.3] | 4.7 [3.0, 6.9] | 9.0 [7.1, 11.4] | 0.14 | 9.6 [8.5, 10.8] | 9.2 [8.2, 10.4] | 10.6 [9.5, 11.8] | 0.10 |
|  |  | **Primary (E2)** |  | 2.3 [1.7, 3.0] | 2.3 [1.8, 2.9] | 3.4 [2.8, 4.1] | 2.4 [1.9, 3.1] | -0.59 | 17.6 [13.9, 21.9] | 16.1 [12.1, 21.1] | -0.30 | 18.0 [13.5, 23.6] | 10.2 [7.7, 13.4] | 10.3 [7.9, 13.2] | -0.68 | 7.6 [6.5, 8.7] | 8.3 [7.2, 9.5] | 11.1 [10.0, 12.3] | 0.31 | 3.0 [2.3, 4.0] | 6.7 [5.2, 8.6] | 3.4 [2.3, 4.8] | 5.1 [4.1, 6.3] | 0.06 | 8.0 [6.9, 9.3] | 7.6 [6.4, 9.1] | 8.4 [7.2, 9.9] | 0.04 |
|  |  | **None (E1)** |  | 3.2 [2.2, 4.7] | 3.0 [2.1, 4.4] | 2.2 [1.4, 3.6] | 1.5 [0.8, 2.8] | -0.53 | 13.2 [11.3, 15.4] | 11.3 [9.7, 13.1] | -0.38 | 12.8 [10.7, 15.3] | 7.6 [6.5, 8.9] | 13.5 [12.0, 15.0] | 0.09 | 7.9 [5.9, 10.4] | 5.3 [3.6, 7.7] | 8.8 [7.2, 10.7] | 0.06 | 2.9 [2.0, 4.1] | 3.6 [2.0, 6.6] | 2.1 [0.8, 4.5] | 5.6 [3.5, 8.8] | 0.12 | 7.4 [5.6, 9.8] | 10.3 [6.0, 17.0] | 2.3 [0.6, 8.3] | -0.51 |
|  |  | **Survey year** |  | 2005 | 2010 | 2014 | 2019 |  | 2005 | 2010 |  | 2008 | 2013 | 2019 |  | 2004 | 2010 | 2015 |  | 2000 | 2006 | 2011 | 2016 |  | 2005 | 2010 | 2015 |  |
|  |  | **Country** |  | Rwanda |  |  |  | AARC (%-points): | Senegal |  | AARC (%-points): | Sierra Leone |  |  | AARC (%-points): | Tanzania |  |  | AARC (%-points): | Uganda |  |  |  | AARC (%-points): | Zimbabwe |  |  | AARC (%-points): |

| **Supplemental Table 6.** (continued)^1^ | **Education level** | **p-value** | **EASTERN MEDITERRANEAN REGION** | 0.0330 | 0.2500 | 0.9280 |  | 0.0210 | 0.0000 | 0.8820 | 0.3110 |  | **EUROPEAN REGION** | - | 0.0000 |  | - | - | - |  | **AMERICAS REGION** | 0.0210 | 0.0000 |  | 0.0000 | 0.0000 | 0.0000 | 0.0000 |  |
| --- | --- | --- | --- | --- | --- | --- | --- | --- | --- | --- | --- | --- | --- | --- | --- | --- | --- | --- | --- | --- | --- | --- | --- | --- | --- | --- | --- | --- | --- |
|  |  | **SII^2^** |  | 4.0 | 2.8 | -0.2 | 0.01 | -10.4 | -12.8 | -0.5 | -3.8 | 0.59 |  | - | -4.5 | - | - | - | - | - |  | -4.9 | -9.6 | -1.00 | 9.0 | 6.6 | 10.7 | 7.6 | 0.14 |
|  |  | **Secondary+ (E3)** |  | 20.9 [18.7, 23.2] | 33.0 [31.1, 34.8] | 21.8 [20.2, 23.6] | -0.09 | 17.5 [14.9, 20.5] | 23.4 [23.3, 23.6] | 25.5 [23.7, 27.5] | 30.5 [28.6, 32.5] | 0.82 |  | 8.0 [6.8, 9.4] | 11.5 [10.4, 12.6] | - | 5.1 [4.4, 5.9] | 10.6 [9.7, 11.5] | 5.7 [4.7, 6.8] | - |  | 13.4 [11.7, 15.3] | 15.6 [13.7, 17.7] | 0.44 | 15.4 [11.5, 20.5] | 11.6 [9.7, 13.8] | 15.8 [14.1, 17.7] | 17.7 [16.1, 19.5] | 0.21 |
|  |  | **Primary (E2)** |  | 21.8 [19.5, 24.3] | 33.2 [30.1, 36.4] | 21.5 [17.8, 25.7] | -0.17 | 28.2 [21.9, 35.5] | 31.1 [29.8, 32.5] | 29.4 [21.9, 38.3] | 36.4 [29.5, 43.9] | 0.46 |  | 9.9 [8.7, 11.3] | 14.0 [12.8, 15.3] | - | - | - | 6.2 [3.4, 11.1] | - |  | 16.8 [14.9, 19.0] | 21.2 [19.0, 23.6] | 0.88 | 12.2 [8.7, 16.8] | 9.6 [7.6, 12.1] | 11.4 [9.9, 13.2] | 16.8 [14.6, 19.1] | 0.27 |
|  |  | **None (E1)** |  | 19.4 [17.6, 21.3] | 30.8 [30.6, 31.1] | 20.2 [17.7, 23.0] | -0.09 | 23.9 [17.6, 31.6] | 26.8 [26.3, 27.2] | 22.4 [14.1, 33.5] | 31.6 [22.4, 42.6] | 0.37 |  | - | 14.3 [7.4, 25.6] | - | - | - | - | - |  | 15.4 [13.3, 17.8] | 21.4 [14.8, 29.9] | 1.20 | 11.4 [9.7, 13.4] | 7.1 [5.6, 8.9] | 9.2 [7.4, 11.5] | 12.5 [10.3, 15.1] | 0.09 |
|  |  | **Survey year** |  | 2000 | 2005 | 2014 |  | 2002 | 2007 | 2012 | 2017 |  |  | 2008 | 2017 |  | 2000 | 2005 | 2015 |  |  | 2003 | 2008 |  | 2000 | 2005 | 2012 | 2016 |  |
|  |  | **Country** |  | Egypt |  |  | AARC (%-points): | Jordan |  |  |  | AARC (%-points): |  | Albania |  | AARC (%-points): | Armenia |  |  | AARC (%-points): |  | Bolivia |  | AARC (%-points): | Haiti |  |  |  | AARC (%-points): |

| **Supplemental Table 6.** (continued)^1^ | **Education level** | **p-value** | **AMERICAS REGION** | 0.3130 | 0.0030 |  | 0.1240 | 0.0580 | 0.8400 | 0.5460 | 0.2190 | 0.4530 |  | **SOUTHEAST ASIAN REGION** | 0.0000 | 0.0000 | 0.0000 |  | 0.0000 | 0.0000 | 0.0000 |  | 0.7190 | 0.0260 |  | **WESTERN PACIFIC REGION** | 0.8410 | 0.9160 | 0.1340 | 0.0020 |  | ^1^Values are percentages and 95% CIs; estimates account for survey design. Estimates for certain categories are missing due to sample size <25. . A positive AARC value depicts an increase in %-points in malnutrition over time; whereas a negative value  means that the prevalence is decreasing. Abbreviations: AARC, average annual rate of change; DRC, Democratic Republic of the Congo.  ^2^The SII represents the absolute difference in the fitted value of co-occurrent overweight/obesity and anemia between the highest and lowest education values. A positive value depicts that the DBM burden is  concentrated among the richest groups; whereas a negative value represents the opposite. Statistical significance= p-value <0.05. |
| --- | --- | --- | --- | --- | --- | --- | --- | --- | --- | --- | --- | --- | --- | --- | --- | --- | --- | --- | --- | --- | --- | --- | --- | --- | --- | --- | --- | --- | --- | --- | --- | --- |
|  |  | **SII^2^** |  | 1.0 | 2.7 | 0.15 | -3.1 | -1.9 | 0.2 | -0.6 | -1.1 | -0.7 | 0.27 |  | 10.8 | 7.1 | 4.4 | -0.19 | 3.1 | 5.0 | 6.0 | 0.52 | -0.3 | 2.6 | 0.29 |  | -0.3 | -0.0 | -1.4 | -3.2 | -0.24 |  |
|  |  | **Secondary+ (E3)** |  | 8.6 [7.6, 9.7] | 9.2 [8.2, 10.1] | 0.10 | 14.5 [13.1, 16.0] | 12.8 [11.9, 13.8] | 11.0 [10.2, 12.0] | 10.3 [9.4, 11.1] | 8.4 [7.8, 9.2] | 9.2 [8.5, 10.0] | -0.49 |  | 10.3 [10.0, 10.6] | 13.2 [13.0, 13.5] | 15.7 [15.5, 15.9] | 0.37 | 3.4 [2.6, 4.3] | 5.1 [3.8, 6.7] | 10.3 [8.7, 12.1] | 0.69 | 1.8 [0.9, 3.7] | 2.9 [2.2, 4.0] | 0.16 |  | 2.2 [1.1, 4.4] | 3.1 [2.2, 4.3] | 3.9 [3.0, 5.1] | 5.6 [4.7, 6.6] | 0.23 |  |
|  |  | **Primary (E2)** |  | 9.3 [8.5, 10.2] | 9.0 [8.3, 9.9] | -0.05 | 18.7 [16.8, 20.7] | 13.7 [12.6, 15.0] | 10.3 [9.2, 11.4] | 10.9 [9.8, 12.1] | 10.0 [9.0, 11.1] | 9.8 [8.7, 10.9] | -0.78 |  | 7.2 [6.7, 7.6] | 11.4 [11.0, 11.8] | 14.8 [14.4, 15.2] | 0.52 | 2.3 [1.1, 4.7] | 3.3 [2.1, 5.3] | 8.0 [5.9, 10.8] | 0.57 | 1.1 [0.5, 2.5] | 3.2 [1.7, 5.8] | 0.30 |  | 3.7 [2.8, 4.9] | 3.9 [3.3, 4.6] | 4.6 [3.8, 5.6] | 8.7 [7.6, 9.9] | 0.32 |  |
|  |  | **None (E1)** |  | 8.0 [6.7, 9.5] | 8.6 [6.6, 11.0] | 0.10 | 11.6 [9.4, 14.2] | 13.1 [10.1, 16.8] | 11.4 [8.9, 14.5] | 9.5 [6.7, 13.3] | 7.4 [5.1, 10.7] | 10.3 [7.9, 13.4] | -0.24 |  | 4.5 [4.2, 4.7] | 8.4 [8.2, 8.6] | 11.5 [11.2, 11.7] | 0.48 | 1.8 [1.3, 2.4] | 2.3 [1.7, 3.3] | 5.8 [4.6, 7.2] | 0.40 | 1.3 [0.7, 2.4] | 1.2 [0.5, 2.6] | -0.01 |  | 3.9 [2.7, 5.6] | 3.4 [2.6, 4.5] | 4.8 [3.5, 6.5] | 8.9 [7.2, 10.9] | 0.34 |  |
|  |  | **Survey year** |  | 2005 | 2011 |  | 2000 | 2007 | 2009 | 2010 | 2011 | 2012 |  |  | 2005 | 2015 | 2019 |  | 2006 | 2011 | 2016 |  | 2009 | 2016 |  |  | 2000 | 2005 | 2010 | 2014 |  |  |
|  |  | **Country** |  | Honduras |  | AARC (%-points): | Peru |  |  |  |  |  | AARC (%-points): |  | India |  |  | AARC (%-points): | Nepal |  |  | AARC (%-points): | Timor-Leste |  | AARC (%-points): |  | Cambodia |  |  |  | AARC (%-points): |  |

| **Supplemental Table 7.** Trends in the prevalence of co-occurrent overweight/obesity and anemia among adult women (20-49 years old) by area of residence^1^ | **Area of residence** | **p-value** | **AFRICAN REGION** | 0.0000 | 0.0000 | 0.0000 |  | 0.0000 | 0.0000 |  | 0.0000 | 0.0027 |  | 0.0000 | 0.0000 | 0.0000 |  | 0.0785 | 0.0000 |  | 0.0000 | 0.0000 |  | 0.0002 | 0.0031 | 0.0000 |  | 0.0145 | 0.5069 |  |
| --- | --- | --- | --- | --- | --- | --- | --- | --- | --- | --- | --- | --- | --- | --- | --- | --- | --- | --- | --- | --- | --- | --- | --- | --- | --- | --- | --- | --- | --- | --- |
|  |  | **Gap^2^** |  | 12.0 | 5.8 | 9.6 | -0.20 | 12.5 | 8.5 | -0.57 | 3.8 | 2.2 | -0.27 | 12.7 | 8.7 | 12.6 | -0.01 | 4.1 | 11.4 | 1.22 | 7.1 | 7.2 | 0.02 | 1.7 | 0.9 | 2.9 | 0.10 | 3.7 | 0.9 | -0.47 |
|  |  | **Rural** |  | 8.3 [6.7, 10.1] | 10.1 [8.8, 11.6] | 11.6 [10.3, 13.1] | 0.20 | 1.8 [1.3, 2.5] | 3.0 [2.5, 3.7] | 0.17 | 0.9 [0.5, 1.5] | 1.9 [1.5, 2.3] | 0.17 | 7.0 [5.8, 8.5] | 8.5 [7.3, 10.0] | 8.9 [7.6, 10.5] | 0.14 | 12.6 [9.4, 16.6] | 8.2 [7.1, 9.5] | -0.73 | 2.8 [2.1, 3.8] | 3.1 [2.3, 4.1] | 0.05 | 0.7 [0.4, 1.3] | 0.5 [0.3, 0.9] | 1.0 [0.7, 1.4] | 0.03 | 11.6 [9.8, 13.6] | 15.8 [13.8, 17.9] | 0.70 |
|  |  | **Total urban** |  | 20.3 [17.5, 23.5] | 15.9 [13.7, 18.5] | 21.2 [19.3, 23.2] | 0.01 | 14.3 [11.5, 17.3] | 11.5 [10.0, 13.2] | -0.40 | 4.7 [3.1, 7.0] | 4.1 [2.5, 6.6] | -0.10 | 19.7 [17.8, 21.6] | 17.2 [15.7, 18.8] | 21.5 [19.3, 23.8] | 0.13 | 16.7 [14.2, 19.6] | 19.6 [16.7, 22.9] | 0.48 | 9.9 [8.4, 11.6] | 10.3 [8.7, 12.2] | 0.07 | 2.4 [1.6, 3.5] | 1.4 [1.0, 2.1] | 3.9 [2.9, 5.2] | 0.13 | 15.3 [13.1, 17.8] | 16.7 [14.8, 18.9] | 0.23 |
|  |  | **Other urban** |  | 16.2 [12.6, 20.7] | 15.0 [12.5, 18.0] | 19.7 [17.7, 21.9] | 0.18 | 13.9 [10.8, 17.3] | 9.7 [8.0, 11.8] | -0.60 | 4.0 [1.6, 9.6] | 3.9 [2.4, 6.2] | -0.02 | 16.0 [14.2, 18.0] | 14.5 [13.0, 16.2] | 16.1 [14.2, 18.2] | 0.01 | 16.4 [13.4, 20.0] | 21.2 [17.7, 25.3] | 0.80 | 6.9 [5.3, 8.7] | 6.6 [5.2, 8.4] | -0.05 | 2.8 [1.8, 4.4] | 1.1 [0.6, 2.0] | 3.5 [2.4, 5.2] | 0.05 | 15.0 [12.7, 17.6] | 16.6 [14.6, 18.8] | 0.27 |
|  |  | **Capital** |  | 27.2 [22.9, 32.1] | 17.9 [13.6, 23.2] | 29.5 [24.4, 35.1] | 0.03 | 15.7 [9.9, 23.3] | 13.4 [10.8, 16.5] | -0.33 | 5.0 [3.1, 8.0] | 4.3 [2.0, 8.8] | -0.12 | 32.9 [28.0, 38.2] | 26.0 [21.9, 30.1] | 38.6 [33.9, 43.5] | 0.41 | 16.9 [13.2, 21.3] | 17.9 [13.4, 23.6] | 0.17 | 15.9 [12.7, 19.5] | 17.8 [14.7, 21.3] | 0.32 | 1.3 [0.5, 3.3] | 2.4 [1.6, 3.6] | 5.0 [3.6, 6.8] | 0.33 | 22.7 [17.3, 29.2] | 24.2 [18.1, 31.6] | 0.25 |
|  |  | **Survey year** |  | 2001 | 2011 | 2017 |  | 2003 | 2010 |  | 2010 | 2016 |  | 2004 | 2011 | 2018 |  | 2005 | 2011 |  | 2007 | 2013 |  | 2005 | 2011 | 2016 |  | 2013 | 2019 |  |
|  |  | **Country *(capital)*** |  | Benin | *(Cotonou)* |  | AARC (%-points): | Burkina Faso | *(Ouagadougou)* | AARC (%-points): | Burundi | *(Bujumbura)* | AARC (%-points): | Cameroon | *(Douala)* |  | AARC (%-points): | Congo | *(Brazzaville)* | AARC (%-points): | DRC | *(Kinshasa)* | AARC (%-points): | Ethiopia | *(Addis Ababa)* |  | AARC (%-points): | Gambia | *(Banjul)* | AARC (%-points): |

| **Supplemental Table 7.** (continued)^1^ | **Area of residence** | **p-value** | **AFRICAN REGION** | 0.0000 | 0.0000 | 0.0000 |  | 0.0000 | 0.0000 | 0.0000 |  | 0.1984 | 0.2505 | 0.1307 |  | 0.0011 | 0.0000 |  | 0.2900 | 0.0105 | 0.0000 |  | 0.0000 | 0.0000 | 0.0000 | 0.0018 |  | 0.0000 | 0.0000 |  |
| --- | --- | --- | --- | --- | --- | --- | --- | --- | --- | --- | --- | --- | --- | --- | --- | --- | --- | --- | --- | --- | --- | --- | --- | --- | --- | --- | --- | --- | --- | --- |
|  |  | **Gap^2^** |  | 6.9 | 11.2 | 7.9 | 0.07 | 10.7 | 8.6 | 6.8 | -0.30 | 2.6 | 2.0 | 2.8 | 0.02 | 2.2 | 2.8 | 0.12 | 2.1 | 2.5 | 5.8 | 0.33 | 10.2 | 12.4 | 8.9 | 5.9 | -0.30 | 9.8 | 11.9 | 0.35 |
|  |  | **Rural** |  | 7.1 [5.9, 8.5] | 13.0 [11.5, 14.7] | 12.6 [10.6, 15.0] | 0.48 | 4.5 [3.7, 5.6] | 5.9 [4.7, 7.4] | 10.3 [8.8, 11.9] | 0.44 | 13.0 [11.2, 15.1] | 10.4 [8.7, 12.4] | 11.4 [9.8, 13.2] | -0.16 | 1.6 [1.0, 2.4] | 1.7 [1.3, 2.3] | 0.02 | 4.9 [3.8, 6.3] | 4.0 [3.3, 4.8] | 5.7 [4.9, 6.6] | 0.07 | 5.9 [4.5, 7.8] | 6.9 [5.5, 8.7] | 6.2 [5.2, 7.3] | 14.3 [12.7, 16.1] | 0.44 | 2.8 [2.0, 4.0] | 5.5 [4.4, 6.8] | 0.45 |
|  |  | **Total urban** |  | 14.0 [12.0, 16.2] | 24.2 [21.7, 27.0] | 20.5 [17.8, 23.5] | 0.55 | 15.2 [12.2, 18.9] | 14.5 [12.5, 16.9] | 17.1 [14.6, 19.9] | 0.14 | 15.6 [12.3, 19.6] | 12.4 [9.7, 15.6] | 14.2 [11.1, 17.8] | -0.14 | 3.8 [2.7, 5.2] | 4.5 [3.3, 6.0] | 0.14 | 7.0 [3.8, 12.5] | 6.5 [4.7, 8.8] | 11.5 [9.4, 13.9] | 0.39 | 16.1 [12.6, 20.5] | 19.3 [13.9, 26.2] | 15.1 [13.1, 17.5] | 20.2 [16.9, 24.0] | 0.14 | 12.6 [10.2, 15.4] | 17.4 [14.7, 20.5] | 0.80 |
|  |  | **Other urban** |  | 11.0 [8.9, 13.5] | 24.0 [20.8, 27.2] | 16.7 [14.1, 19.7] | 0.46 | 10.2 [6.4, 15.7] | 14.0 [10.8, 17.9] | 15.7 [12.4, 19.6] | 0.43 | 17.8 [14.6, 21.6] | 14.5 [10.7, 19.4] | 12.9 [10.5, 15.9] | -0.49 | 4.2 [2.7, 6.4] | 5.6 [4.1, 7.7] | 0.28 | 5.5 [2.9, 10.4] | 5.4 [3.5, 8.3] | 11.7 [9.2, 14.8] | 0.54 | 16.0 [9.7, 25.4] | 20.7 [12.7, 31.7] | 11.1 [8.9, 13.9] | 17.7 [13.2, 23.3] | -0.09 | 10.5 [7.5, 14.6] | 14.0 [10.5, 18.4] | 0.58 |
|  |  | **Capital** |  | 20.4 [16.5, 25.1] | 25.0 [20.3, 30.3] | 27.3 [21.6, 33.9] | 0.62 | 21.7 [16.6, 27.7] | 14.9 [12.4, 18.0] | 18.6 [15.1,22.9] | -0.26 | 13.3 [7.6, 22.1] | 9.6 [6.4, 14.4] | 15.6 [9.8, 23.8] | 0.23 | 3.1 [1.9, 5.1] | 2.2 [1.1, 4.7] | -0.18 | 11.0 [2.9, 33.9] | 7.7 [4.8, 12.2] | 11.2 [7.9, 15.5] | -0.00 | 16.2 [13.0, 20.0] | 17.2 [14.1, 20.7] | 19.2 [15.7, 23.3] | 21.6 [17.2, 26.8] | 0.32 | 15.0 [11.2, 19.8] | 21.9 [18.1, 26.3] | 1.15 |
|  |  | **Survey year** |  | 2003 | 2008 | 2014 |  | 2005 | 2012 | 2018 |  | 2004 | 2009 | 2014 |  | 2003 | 2008 |  | 2004 | 2010 | 2015 |  | 2001 | 2006 | 2012 | 2018 |  | 2006 | 2012 |  |
|  |  | **Country *(capital)*** |  | Ghana | *(Accra)* |  | AARC (%-points): | Guinea | *(Conakry)* |  | AARC (%-points): | Lesotho | *(Maseru)* |  | AARC (%-points): | Madagascar | *(Antananarivo)* | AARC (%-points): | Malawi | *(Lilongwe)* |  | AARC (%-points): | Mali | *(Bamako)* |  |  | AARC (%-points): | Niger | *(Niamey)* | AARC (%-points): |

| **Supplemental Table 7.** (continued)^1^ | **Area of residence** | **p-value** | **AFRICAN REGION** | 0.0042 | 0.0024 | 0.0000 | 0.0001 |  | 0.0000 | 0.0000 |  | 0.0003 | 0.0001 | 0.0004 |  | 0.0000 | 0.0000 | 0.0000 |  | 0.0000 | 0.3203 | 0.0020 | 0.0018 |  | 0.0000 | 0.0000 | 0.0000 |  |
| --- | --- | --- | --- | --- | --- | --- | --- | --- | --- | --- | --- | --- | --- | --- | --- | --- | --- | --- | --- | --- | --- | --- | --- | --- | --- | --- | --- | --- |
|  |  | **Gap^2^** |  | 2.3 | 2.0 | 2.7 | 2.9 | 0.05 | 10.0 | 9.7 | -0.06 | 7.5 | 4.7 | 4.1 | -0.30 | 9.8 | 9.2 | 9.8 | -0.00 | 3.8 | 1.7 | 3.0 | 3.5 | 0.00 | 7.7 | 4.3 | 5.6 | -0.21 |
|  |  | **Rural** |  | 2.5 [1.9, 3.2] | 2.4 [1.9, 3.0] | 2.9 [2.4, 3.5] | 2.2 [1.8, 2.8] | -0.31 | 10.0 [8.4, 11.9] | 7.9 [6.6, 9.5] | -0.42 | 11.7 [9.5, 14.2] | 6.8 [5.8, 8.0] | 11.1 [9.8, 12.6] | -0.03 | 5.6 [4.6, 6.7] | 5.9 [5.0, 7.0] | 8.4 [7.5, 9.5] | 0.25 | 2.8 [2.2, 3.6] | 5.8 [4.6, 7.4] | 2.6 [1.8, 3.7] | 5.5 [4.4, 6.7] | 0.10 | 6.0 [5.1, 7.2] | 7.2 [6.3, 8.2] | 7.7 [6.6, 8.9] | 0.17 |
|  |  | **Total urban** |  | 4.8 [3.3, 6.9] | 4.4 [3.4, 5.6] | 5.6 [4.5, 7.1] | 5.1 [3.6, 7.1] | -0.58 | 20.0 [17.1, 23.3] | 17.6 [14.7, 21.0] | -0.48 | 19.2 [16.0, 23.0] | 11.5 [9.4, 14.1] | 15.2 [13.4, 17.1] | -0.33 | 15.4 [13.3, 17.8] | 15.1 [12.9, 17.7] | 18.2 [16.4, 20.2] | 0.24 | 6.6 [4.8, 8.9] | 7.5 [4.7, 11.8] | 5.6 [3.8, 7.9] | 9.0 [7.1, 11.4] | 0.10 | 13.7 [12.2, 15.2] | 11.5 [9.7, 13.5] | 13.3 [11.7, 15.1] | -0.04 |
|  |  | **Other urban** |  | 3.8 [1.9, 7.1] | 3.7 [3.7, 3.8] | 6.1 [4.3, 8.5] | 3.5 [2.3, 5.4] | -0.57 | 19.0 [16.1, 22.2] | 13.2 [11.4, 15.2] | -1.16 | 17.7 [13.6, 22.7] | 13.0 [10.7, 15.7] | 13.5 [11.1, 16.3] | -0.37 | 12.8 [10.5, 15.5] | 11.5 [9.3, 14.2] | 15.0 [13.1, 17.0] | 0.19 | 6.2 [4.2, 9.0] | 4.8 [2.0, 11.1] | 5.1 [3.0, 8.2] | 8.1 [6.0, 10.9] | 0.11 | 14.8 [13.0, 16.9] | 12.8 [10.7, 15.2] | 12.9 [11.3, 14.7] | -0.19 |
|  |  | **Capital** |  | 6.1 [3.8, 9.6] | 4.7 [3.3, 6.8] | 5.3 [3.8, 7.3] | 6.3 [4.0, 9.8] | -0.61 | 20.8 [16.1, 26.3] | 21.2 [16.3, 27.2] | 0.08 | 20.4 [15.8, 26.0] | 10.2 [7.0, 14.5] | 16.7 [14.3, 19.4] | -0.29 | 21.2 [16.3, 27.1] | 24.8 [19.6, 30.8] | 25.1 [21.4, 29.2] | 0.36 | 6.8 [4.4, 10.3] | 10.2 [5.8, 17.1] | 6.3 [3.5, 10.4] | 12.2 [8.3, 17.6] | 0.23 | 12.1 [9.9, 14.8] | 10.1 [7.5, 13.5] | 13.8 [10.9, 17.3] | 0.17 |
|  |  | **Survey year** |  | 2005 | 2010 | 2014 | 2019 |  | 2005 | 2010 |  | 2008 | 2013 | 2019 |  | 2004 | 2010 | 2015 |  | 2000 | 2006 | 2011 | 2016 |  | 2005 | 2010 | 2015 |  |
|  |  | **Country *(capital)*** |  | Rwanda | *(Kigali)* |  |  | AARC (%-points): | Senegal | *(Dakar)* | AARC (%-points): | Sierra Leone | *(Freetown)* |  | AARC (%-points): | Tanzania | *(Dar es Salaam)* |  | AARC (%-points): | Uganda | *(Kampala)* |  |  | AARC (%-points): | Zimbabwe | *(Harare)* |  | AARC (%-points): |

| **Supplemental Table 7.** (continued)^1^ | **Area of residence** | **p-value** | **EASTERN MEDITERRANEAN REGION** | 0.0273 | 0.0000 | 0.5735 |  | 0.6617 | 0.0034 | 0.5412 | 0.2102 |  | **EUROPEAN REGION** | 0.0000 | 0.1685 |  | 0.0000 | 0.1294 | 0.5192 |  | **AMERICAS REGION** | 0.6785 | 0.4470 |  | 0.0000 | 0.0000 | 0.0000 | 0.0000 |  |
| --- | --- | --- | --- | --- | --- | --- | --- | --- | --- | --- | --- | --- | --- | --- | --- | --- | --- | --- | --- | --- | --- | --- | --- | --- | --- | --- | --- | --- | --- |
|  |  | **Gap^2^** |  | 3.1 | 5.7 | 0.8 | -0.21 | 1.1 | 1.5 | -1.1 | -2.7 | -0.28 |  | -4.4 | 1.1 | 0.61 | -4.2 | 1.7 | 0.6 | 0.26 |  | 0.6 | 1.3 | 0.14 | 10.3 | 8.6 | 7.4 | 7.5 | -0.18 |
|  |  | **Rural** |  | 19.0 [17.5, 20.5] | 29.9 [28.2, 31.5] | 21.1 [19.3, 22.9] | 0.02 | 18.4 [14.9, 22.6] | 23.0 [21.8, 24.2] | 26.7 [23.8, 29.9] | 33.3 [29.6, 37.2] | 0.97 |  | 11.1 [9.8, 12.6] | 12.0 [10.9, 13.1] | 0.10 | 7.8 [6.5, 9.2] | 9.5 [8.0, 11.3] | 5.3 [4.2, 6.8] | -0.20 |  | 14.8 [12.6, 17.3] | 17.6 [15.1, 20.4] | 0.56 | 8.0 [6.4, 10.0] | 5.5 [4.4, 6.9] | 9.5 [8.0, 11.3] | 13.0 [11.5, 14.5] | 0.34 |
|  |  | **Total urban** |  | 22.1 [19.9, 24.5] | 35.6 [33.7, 37.6] | 21.9 [19.7, 24.3] | -0.19 | 19.5 [16.5, 22.9] | 24.5 [24.3, 24.6] | 25.6 [23.5, 27.8] | 30.6 [28.6, 32.7] | 0.69 |  | 6.7 [5.6, 8.1] | 13.1 [11.9, 14.4] | 0.71 | 3.6 [2.8, 4.5] | 11.2 [9.8, 12.9] | 5.9 [4.7, 7.5] | 0.06 |  | 15.4 [13.5, 17.6] | 18.9 [17.1, 20.9] | 0.70 | 18.3 [14.8, 22.5] | 14.1 [11.8, 16.7] | 16.9 [15.1, 18.9] | 20.5 [18.6, 22.6] | 0.17 |
|  |  | **Other urban** |  | 19.8 [17.7, 22.1] | 36.4 [33.8, 39.0] | 23.7 [21.2, 26.4] | 0.08 | 22.9 [18.4, 28.2] | 23.6 [23.2, 24.1] | 29.5 [26.8, 32.3] | 30.7 [28.8, 32.8] | 0.59 |  | 8.2 [6.6, 10.1] | 12.6 [11.5, 13.8] | 0.49 | 5.4 [4.3, 6.7] | 11.2 [9.5, 13.3] | 5.4 [4.4, 6.6] | -0.08 |  | 13.2 [11.4, 15.2] | 16.0 [14.0, 18.3] | 0.56 | 15.0 [12.1, 18.3] | 11.6 [9.3, 14.3] | 16.0 [14.0, 18.2] | 19.5 [17.4, 21.9] | 0.33 |
|  |  | **Capital** |  | 24.9 [20.8, 29.5] | 34.6 [31.7, 37.7] | 18.6 [14.5, 23.5] | -0.61 | 18.2 [14.6, 22.5] | 22.9 [20.5, 25.3] | 23.8 [21.0, 26.9] | 30.5 [26.8, 34.3] | 0.76 |  | 4.2 [2.9, 6.0] | 13.8 [11.5, 16.6] | 1.07 | 2.2 [1.2, 3.7] | 11.2 [9.0, 13.9] | 6.4 [4.3, 9.4] | 0.17 |  | 21.5 [16.4, 27.7] | 25.0 [21.3, 29.1] | 0.70 | 19.3 [15.0, 24.6] | 15.6 [12.3, 19.6] | 17.7 [14.9, 20.8] | 21.3 [18.3, 24.5] | 0.14 |
|  |  | **Survey year** |  | 2000 | 2005 | 2014 |  | 2002 | 2007 | 2012 | 2017 |  |  | 2008 | 2017 |  | 2000 | 2005 | 2015 |  |  | 2003 | 2008 |  | 2000 | 2005 | 2012 | 2016 |  |
|  |  | **Country *(capital)*** |  | Egypt | *(Cairo)* |  | AARC (%-points): | Jordan | *(Amman)* |  |  | AARC (%-points): |  | Albania | *(Tirana)* | AARC (%-points): | Armenia | *(Yerevan)* |  | AARC (%-points): |  | Bolivia | *(La Paz)* | AARC (%-points): | Haiti | *(Port-au-Prince)* |  |  | AARC (%-points): |

| **Supplemental Table 7.** (continued)^1^ | **Area of residence** | **p-value** | **AMERICAS REGION** | 0.0005 | 0.0000 |  | 0.0915 | 0.0000 | 0.0000 | 0.0172 | 0.0264 | 0.4676 |  | **SOUTHEAST ASIAN REGION** | 0.0000 | 0.0000 | 0.0000 |  | 0.0000 | 0.0000 | 0.0031 |  | 0.0004 | 0.0000 |  | **WESTERN PACIFIC REGION** | 0.9827 | 0.2684 | 0.3142 | 0.1435 |  | ^1^Values are percentages and 95% CIs; estimates account for survey design. The estimate for women living in the capital and other urban areas is missing for Nepal 2011, as the survey does not identify whether urban citizens are living in the  capital city or elsewhere. A positive AARC value depicts an increase in %-points in malnutrition over time; whereas a negative value means that the prevalence is decreasing Abbreviations: AARC, average annual rate of change; DRC,  Democratic Republic of the Congo.  **^2^**Gaps are expressed in percentage points and indicate the difference between urban and rural areas (urban-rural). Statistical significance= p-value <0.05. |
| --- | --- | --- | --- | --- | --- | --- | --- | --- | --- | --- | --- | --- | --- | --- | --- | --- | --- | --- | --- | --- | --- | --- | --- | --- | --- | --- | --- | --- | --- | --- | --- | --- |
|  |  | **Gap^2^** |  | 2.4 | 3.1 | 0.12 | 2.3 | 3.2 | 3.1 | 1.6 | 1.5 | -0.4 | -0.15 |  | 7.7 | 8.5 | 6.9 | 0.08 | 2.5 | 4.3 | 3.2 | 0.07 | 2.5 | 3.7 | 0.17 |  | -0.1 | 0.7 | 0.8 | 1.2 | 0.09 |  |
|  |  | **Rural** |  | 7.7 [7.0, 8.6] | 7.4 [6.7, 8.1] | -0.05 | 13.9 [11.9, 16.1] | 10.8 [9.8, 11.8] | 8.5 [7.5, 9.6] | 9.2 [8.3, 10.2] | 7.7 [6.8, 8.8] | 9.7 [8.8, 10.8] | -0.46 |  | 4.2 [4.0, 4.4] | 8.4 [8.3, 8.5] | 12.2 [12.0, 12.4] | 0.54 | 1.8 [1.3, 2.5] | 2.9 [2.2, 3.7] | 6.0 [4.8, 7.6] | 0.42 | 0.8 [0.5, 1.5] | 1.3 [0.8, 1.9] | 0.07 |  | 3.6 [2.8, 4.6] | 3.5 [3.0, 4.1] | 4.3 [3.5, 5.2] | 7.4 [6.6, 8.4] | 0.25 |  |
|  |  | **Total urban** |  | 10.1 [9.1, 11.3] | 10.5 [9.7, 11.4] | 0.07 | 16.2 [14.7, 17.9] | 14.0 [13.0, 14.9] | 11.6 [10.8, 12.6] | 10.8 [10.0, 11.7] | 9.2 [8.5, 9.9] | 9.3 [8.6, 10.0] | -0.61 |  | 11.9 [11.6, 12.2] | 16.9 [16.5, 17.3] | 19.1 [18.7, 19.5] | 0.51 | 4.3 [3.4, 5.3] | 7.2 [5.7, 9.0] | 9.2 [7.8, 10.8] | 0.49 | 3.3 [1.8, 6.2] | 5.0 [3.5, 7.2] | 0.24 |  | 3.5 [2.0, 6.3] | 4.2 [3.2, 5.6] | 5.1 [3.9, 6.7] | 8.6 [7.4, 9.9] | 0.34 |  |
|  |  | **Other urban** |  | 11.7 [10.4, 13.1] | 10.9 [9.9, 12.0] | -0.13 | 14.2 [12.5, 16.1] | 13.1 [12.2, 14.0] | 10.7 [9.9, 11.6] | 10.5 [9.7, 11.4] | 9.1 [8.4, 9.9] | 10.0 [9.3, 10.7] | -0.41 |  | 11.9 [11.6, 12.2] | 16.8 [16.4, 17.2] | 19.1 [18.7, 19.5] | 0.51 | 4.8 [3.8, 6.1] | - | 8.4 [7.0, 10.1] | 0.36 | 1.7 [0.7, 3.7] | 3.6 [2.2, 5.8] | 0.27 |  | 4.8 [2.6, 8.7] | 5.3 [4.2, 6.7] | 6.1 [4.7, 7.8] | 9.3 [8.1, 10.7] | 0.30 |  |
|  |  | **Capital** |  | 6.6 [5.1, 8.5] | 9.4 [7.8, 11.3] | 0.47 | 18.5 [15.9, 21.5] | 15.4 [13.4, 17.6] | 15.4 [13.4, 17.6] | 11.3 [9.6, 13.1] | 9.2 [8.0, 10.7] | 8.3 [7.0, 9.7] | -0.84 |  | 12.0 [11.7, 12.4] | 19.6 [17.2, 22.2] | 22.8 [21.5, 24.2] | 0.67 | 3.7 [2.4, 5.5] | - | 11.2 [8.3, 15.0] | 0.75 | 4.2 [2.0, 8.6] | 5.6 [3.5, 8.6] | 0.20 |  | 1.1 [0.1, 10.3] | 2.3 [0.7, 7.5] | 4.3 [2.5, 7.2] | 7.8 [6.0, 10.2] | 0.46 |  |
|  |  | **Survey year** |  | 2005 | 2011 |  | 2000 | 2007 | 2009 | 2010 | 2011 | 2012 |  |  | 2005 | 2015 | 2019 |  | 2006 | 2011 | 2016 |  | 2009 | 2016 |  |  | 2000 | 2005 | 2010 | 2014 |  |  |
|  |  | **Country *(capital)*** |  | Honduras | *(Tegucigalpa)* | AARC (%-points): | Peru | *(Lima)* |  |  |  |  | AARC (%-points): |  | India | *(New Delhi)* |  | AARC (%-points): | Nepal | *(Kathmandu)* |  | AARC (%-points): | Timor-Leste | *(Dili)* | AARC (%-points): |  | Cambodia | *(Phnom Penh)* |  |  | AARC (%-points): |  |

**Supplemental Table 8.** Average annual rate of change (AARC) in the prevalence of the double burden of overweight/ obesity and anemia among adult women (20-49 years old) living in the Western and Central African (WCA) and Eastern and Southern African (ESA) subregions.

|  | **WCA^2^**  (n=31 surveys from 12 countries; 110,440 women) | **ESA^3^**  (n=27 surveys from 9 countries; 128,361 women) |
| --- | --- | --- |
| **Co-occurrent overweight/obesity and anemia** | | |
| **Total** | 0.24*(0.08, 0.40) | 0.09*(0.01, 0.18) |
| **Wealth** | | |
| Poorest (Q1) | 0.13 (-0.01, 0.28) | 0.01 (-0.06, 0.08) |
| Poorer (Q2) | 0.35**(0.18, 0.52) | 0.04 (-0.04, 0.13) |
| Middle (Q3) | 0.32**(0.15, 0.50) | 0.11*(0.02, 0.21) |
| Richer (Q4) | 0.35*(0.12, 0.57) | 0.09 (-0.05, 0.23) |
| Richest (Q5) | 0.06 (-0.18, 0.29) | 0.13 (-0.12, 0.27) |
| **Education** | | |
| None (E1) | 0.30**(0.16, 0.45) | 0.01 (-0.14, 0.16) |
| Primary (E2) | 0.06 (-0.17, 0.29) | 0.05 (-0.05, 0.14) |
| Secondary+ (E3) | -0.05 (-0.25, 0.15) | 0.07 (-0.02, 0.16) |
| **Residence** | | |
| Capital | 0.19 (-0.08, 0.47) | 0.17*(0.02, 0.32) |
| Other urban area | 0.06 (-0.18, 0.30) | 0.06 (-0.09, 0.21) |
| Total urban | 0.08 (-0.11, 0.28) | 0.10 (-0.01, 0.21) |
| Rural area | 0.28**(0.12, 0.43) | 0.06 (-0.02, 0.15) |
| **Overweight/obesity** | | |
| **Total** | 0.82**(0.54, 1.11) | 0.84**(0.67, 1.02) |
| **Anemia** | | |
| **Total** | -0.46*(-0.87, -0.06) | -0.67*(-1.16, -0.20) |

^1^Values are average annual rates of change (AARC) (95% CIs) expressed in %-points unless otherwise indicated. A positive AARC value depicts an increase in %-points in malnutrition over time; whereas a negative value means that the prevalence is decreasing. ^*,**^Significance^: *^*P*<0.05, ^**^*P*<0.001.

^2^WCA includes: Benin, Burkina Faso, Cameroon, Congo, DRC, Gambia, Ghana, Guinea, Mali, Niger, Senegal, Sierra Leone. ^3^ESA includes: Burundi, Ethiopia, Lesotho, Madagascar, Malawi, Rwanda, Tanzania, Uganda, Zimbabwe.
